# Heterogeneous effects of climatic conditions on Andean bean landraces and cowpeas highlight alternatives for crop management and conservation

**DOI:** 10.1038/s41598-022-10277-x

**Published:** 2022-04-21

**Authors:** Pablo G. Acosta-Quezada, Edin H. Valladolid-Salinas, Janina M. Murquincho-Chuncho, Eudaldo Jadán-Veriñas, Mario X. Ruiz-González

**Affiliations:** 1 Departamento de CC. Biológicas y Agropecuarias, Universidad Técnica Particular de Loja-UTPL, San Cayetano Alto, Calle Marcelino Champagnat s/n, Apartado Postal 11-01-608, Loja, Ecuador; 2 Facultad de CC. Agropecuarias, Universidad Técnica de Machala-UTMACH, Machala, El Oro Ecuador; 3 Present Address: Instituto Universitario de Conservación y Mejora de la Agrodiversidad Valenciana, Universitat Politècnica de València, Camino de Vera s/n, Valencia, Spain

**Keywords:** Agroecology, Climate-change ecology

## Abstract

The use and conservation of agrobiodiversity have become critical to face the actual and future challenges imposed by climate change. Collecting phytogenetic resources is a first step for their conservation; however, the genetic material must be analysed to understand their potential to improve agricultural resilience and adaptation to the new climatic conditions. We have selected nine *Phaseolus vulgaris*, one *P. lunatus* and two *Vigna unguiculata* landraces from two different climatic backgrounds of the Andean region of South Ecuador and one *P. vulgaris* commercial cultivar, and we grew them under two different conditions of temperature and humidity (open field and greenhouse). Then, we recorded data for 32 characters of plant architecture, flower and fruit characteristics and yield, and 17 events in the phenology of the plants. We analysed the impact of treatment on species, climatic background, and each of the landraces, and identified both characters and landraces that are mostly affected by changes in their environmental conditions. Overall, higher temperatures were benign for all materials except for two *P. vulgaris* landraces from cold background, which performed better or developed faster under cold conditions. Finally, we calculated a climate resilience landrace index, which allowed us to classify the landraces by their plasticity to new environmental conditions, and found heterogeneous landrace susceptibility to warmer conditions. Two *P. vulgaris* landraces were highlighted as critical targets for conservation.

## Introduction

Improving agricultural resilience and adaptation to climate change effects is a priority to ensure crop production and food security in the upcoming years^1^. Climate change modifies the trends in temperature and precipitation, thus affecting the response to environmental factors of many species at different geographical levels, with drastic effects and, often, a negative impact on crops^2,3^. However, there is a consensus among different projections that indicate negative impacts of the increase in temperatures on the main crops in many agricultural regions^4^. Moreover, the more updated models with improved and diverse scenarios produce more pessimistic projections for yield responses for maize, rice, and soy bean, although wheat could benefit from higher CO_2_ concentrations^5^. Supporting the latter predictions, the analysis of combined published results of different analytical methods highlights the vulnerability of agriculture to climate change, suggesting a yield reduction of around 3.0 to 6.0% for each degree increase in temperature^6^. Besides the direct effects of temperature and rainfall on crops and plant diseases^7^, climate change has important economic consequences on agriculture^8^. However, the impact of climate change effects on crops depends on both the crop identity and its geographic location^9^.

Plants exhibit the ability to cope with changes in their environment via phenotypic plasticity^10^. Therefore, prior to developing crop improvement or conservation programmes, either to face climate change challenges or to choose appropriate crop varieties that might perform well under certain local conditions, it is indispensable to gain knowledge on the plant plasticity and its potential adaptability to abiotic factors^11^. Moreover, the study of crop phenotypic plasticity must include the analysis of phenology, in addition to other critical traits related to plant morphology and reproduction, because phenology is highly susceptible to changes in the environmental factors^12^. Thus, the information gathered in this way might allow: (1) to select those varieties suitable for a particular range of environmental conditions, with important consequences for local community development, sustainable agriculture and food security; (2) it might help to identify those varieties more prone to suffer negative effects, and thus, allowing their conservation before their effective loss due to the abandoning of low yield landraces^13^; (3) the overall information might provide useful to develop specific indexes aimed at quantifying the resilience potential of either one species or landrace or a particular character.

Beans are a major food resource grown worldwide and represent the main source of protein for many societies, thus playing a vital role in the human diet of developing societies^14^. Beans (*Phaseolus* spp) originated in the New World. Common bean (*P. vulgaris*) had two main domestication centres at Middle and Andean South America, with four major genetic groups in Mesoamerica, Colombia, Northern Andes of Ecuador and north Peru, and the southern Andes; and exhibits both a wide morphological variability (> 40,000 varieties), and adaptation to a broad array of environments^15–18^. Lima beans (*P. lunatus*), distributed from northern Mexico to northern Argentina, have three major genetic groups, two in Mesoamerica and one in the Andes (southern Ecuador and northern Peru), which is the most likely origin of the species^19–21^. Moreover, there exists great diversity of wild bean species with potential to improve the resistance to environmental factors in common bean crops^22^. In addition, Latin America represents about the 50% of the bean world production, followed by Africa; per capita consumption of beans in these regions can oscillate between 12 to 60 kg per year and represent a significant source of protein^14,23^. In Ecuador, beans, which are commonly named *fréjol*, *fríjol* and *poroto* (*P. vulgaris*) *and torta* (*P. lunatus*), belong to the genus *Phaseolus* spp., while the cowpeas, named *vaina,* are *Vigna* spp., originated in Africa^24^. Both species represent the main leguminous crops with a soil surface around 32,817 ha devoted for their culture, with an overall production of 27,492 t^25^. Moreover, as in many other countries, most producers are smallholder farmers that grow beans mainly for self-consume, whether as crop rotation or associated to maize, and thus, beans are an important contribution to Ecuador food sovereignty^26^.

There are robust projections predicting a generalized decline in crops yield due to the impact of climate change, and highlighting the urgency of further research on the effects of high temperatures and other factors on crops to gain a better understanding on the uncertainties of production impacts^5,9,27^. We lack specific predictions; however, for the impact of climate change effects on the production of legumes for the Andean region, where a negative impact on the production of cereals is expected^9^. The effects of climate change on the acceleration of phenological aspects of crops can be counteracted by shifting existing varieties into different regions^28^. Thus, it is pivotal to promote the investigation of the impact of abiotic stresses on landraces; defined by Casañas et al*.*^29^ as those “cultivated varieties that have evolved and may continue evolving, using conventional or modern breeding techniques, in traditional or new agricultural environments within a defined ecogeographical area and under the influence of the local human culture”.

Several studies have focused on the effects that different aspects of climate change and abiotic stress factors have on the common bean. For example, drought, the most extensively studied factor, has drastic effects on legumes because it accelerates plant maturation in *Phaseolus* spp. and *Vigna* spp., among other effects, and reduces yield components and biomass^14,30,31^; elevated CO_2_ concentrations have direct positive effects on stem mass, and a strong genotype × CO_2_ interaction for pod number, seed mass and yield on *P. vulgaris*^32^; and high temperatures negatively affect reproduction, fertilization, and post-fertilization^33^; Lima beans (*P. lunatus*); however, are more tolerant to heat than *P. vulgaris*^14^.

In the present work, we have investigated the effects of two different environmental conditions on the architecture, reproduction, yield, and phenology by using standard agromorphologic and phenological descriptors on 12 landraces of *P. vulgaris*, *P. lunatus*, and *Vigna unguiculata* sampled from different localities at the Andes of south Ecuador, and a commercial *P. vulgaris* cultivar (Supporting Tables S1 and S2; Supporting Fig. S1). Moreover, to test for potential adaptation or conditioning to local environmental factors, four *P. vulgaris* and the *P. lunatus* landraces came from cold background locations, and five *P. vulgaris* and the two *V. unguiculata* landraces, came from warm background locations. Then, to understand the implications of the results better, we calculated an index, the climate resilience landrace index, with potential application in decision-making.

## Results

A summary describing all plant architecture, flower, fruit, and yield, and phenological traits for each of the thirteen *Phaseolus* sp. and *Vigna* sp. landraces in the open field and the greenhouse conditions is provided in Supporting Tables S3, S4 and S5. Main effects Kruskal–Wallis tests are summarised in Table 1, and the interactions between treatment conditions (open field and greenhouse) and species, and landrace and climatic background are summarised in Table 2.

**Table 1:** Main effects Kruskal–Wallis *H* tests for treatment (open field vs greenhouse conditions), species, landrace, and climatic background of the landraces.

Trait	N	Treatment			Species			Landrace			Climatic background		
		*χ*^*2*^	d.f	*p*-value	*χ*^*2*^	d.f	*p*-value	*χ*^*2*^	d.f	*p*-value	*χ*^*2*^	d.f	*p*-value
Q1	430	0.016	1	0.899	37.969	2	** < 0.001**	124.526	12	** < 0.001**	48.404	2	** < 0.001**
Q2	430	10.747	1	**0.001**	171.228	2	** < 0.001**	261.879	12	** < 0.001**	21.548	2	** < 0.001**
Q4	406	4.701	1	**0.030**	86.423	2	** < 0.001**	115.854	12	** < 0.001**	10.485	2	**0.005**
Q6	403	5.411	1	**0.020**	23.312	2	** < 0.001**	130.821	12	** < 0.001**	14.061	2	**0.001**
Q7	403	11.109	1	**0.001**	18.561	2	** < 0.001**	103.558	12	** < 0.001**	19.46	2	** < 0.001**
Q8	403	0.695	1	0.405	90.5	2	** < 0.001**	188.058	12	** < 0.001**	95.678	2	** < 0.001**
Q9	403	6.074	1	**0.014**	59.541	2	** < 0.001**	128.84	12	** < 0.001**	2.604	2	0.272
Q10	403	1.763	1	0.184	38.088	2	** < 0.001**	154.093	12	** < 0.001**	62.888	2	** < 0.001**
Q11	403	4.127	1	**0.042**	192.539	2	** < 0.001**	258.348	12	** < 0.001**	144.881	2	** < 0.001**
Q12	473	90.657	1	** < 0.001**	55.568	2	** < 0.001**	193.255	12	** < 0.001**	11.161	2	**0.004**
Q15	225	1.688	1	0.194	73.326	1	** < 0.001**	111.065	9	** < 0.001**	5.253	2	0.072
Q16	280	0.072	1	0.788	146.016	2	** < 0.001**	223.617	11	** < 0.001**	31.152	2	** < 0.001**
Q17	280	0.153	1	0.695	153.876	2	** < 0.001**	227.185	11	** < 0.001**	33.93	2	** < 0.001**
Q18	280	0.279	1	0.597	177.567	2	** < 0.001**	243.794	11	** < 0.001**	120.701	2	** < 0.001**
Q19	280	0.002	1	0.960	167.381	2	** < 0.001**	231.315	11	** < 0.001**	71.624	2	** < 0.001**
Q20	280	0.634	1	0.426	139.151	2	** < 0.001**	235.354	11	** < 0.001**	31.825	2	** < 0.001**
Q21	529	116.512	1	** < 0.001**	38.277	2	** < 0.001**	156.051	12	** < 0.001**	5.409	2	0.067
Q22	495	21.212	1	** < 0.001**	74.518	2	** < 0.001**	146.631	11	** < 0.001**	3.115	2	0.211
Q23	328	0.189	1	0.664	175.076	2	** < 0.001**	235.516	11	** < 0.001**	8.165	2	**0.017**
Q24	328	5.577	1	**0.018**	155.627	2	** < 0.001**	274.06	11	** < 0.001**	97.224	2	** < 0.001**
Q25	328	0.156	1	0.693	84.384	2	** < 0.001**	241.088	11	** < 0.001**	50.495	2	** < 0.001**
Q26	328	1.354	1	0.245	204.36	2	** < 0.001**	268.468	11	** < 0.001**	58.995	2	** < 0.001**
Q27	530	78	1	** < 0.001**	28.938	2	** < 0.001**	184.174	12	** < 0.001**	21.574	2	** < 0.001**
Q28	328	0.068	1	0.795	193.033	2	** < 0.001**	277.302	11	** < 0.001**	120.085	2	** < 0.001**
Q29	328	2.703	1	0.100	203.039	2	** < 0.001**	305.138	11	** < 0.001**	140.068	2	** < 0.001**
Q30	328	1.987	1	0.159	170.058	2	** < 0.001**	259.778	11	** < 0.001**	8.046	2	**0.018**
Q31	328	0.052	1	0.819	108.646	2	** < 0.001**	315.705	11	** < 0.001**	71.385	2	** < 0.001**
Q32	480	55.431	1	** < 0.001**	96.047	2	** < 0.001**	196.245	12	** < 0.001**	25.666	2	** < 0.001**
Q33	480	109.226	1	** < 0.001**	68.255	2	** < 0.001**	164.348	12	** < 0.001**	14.125	2	**0.001**
Q34	480	115.624	1	** < 0.001**	67.901	2	** < 0.001**	163.759	12	** < 0.001**	13.223	2	**0.001**
Q35	480	135.761	1	** < 0.001**	35.137	2	** < 0.001**	150.532	12	** < 0.001**	9.608	2	**0.008**
P8	530	8.556	1	**0.003**	48.544	2	** < 0.001**	152.832	12	** < 0.001**	44.777	2	** < 0.001**
P9	530	2.981	1	0.084	101.894	2	** < 0.001**	240.239	12	** < 0.001**	74.591	2	** < 0.001**
P10	530	6.551	1	**0.010**	45.869	2	** < 0.001**	199.644	12	** < 0.001**	63.261	2	** < 0.001**
P12	530	6.805	1	**0.009**	31.488	2	** < 0.001**	203.986	12	** < 0.001**	38.753	2	** < 0.001**
P13	530	53.204	1	** < 0.001**	24.695	2	** < 0.001**	174.214	12	** < 0.001**	71.835	2	** < 0.001**
P19	522	114.376	1	** < 0.001**	136.205	2	** < 0.001**	205.614	12	** < 0.001**	10.007	2	**0.007**
P21	522	187.807	1	** < 0.001**	1.474	2	0.478	30.469	12	**0.002**	1.163	2	0.559
P51	486	24.991	1	** < 0.001**	212.581	2	** < 0.001**	311.948	12	** < 0.001**	19.569	2	** < 0.001**
P55	485	34.197	1	** < 0.001**	219.337	2	** < 0.001**	307.497	12	** < 0.001**	18.317	2	** < 0.001**
P59	481	42.641	1	** < 0.001**	216.806	2	** < 0.001**	299.874	12	** < 0.001**	16.137	2	** < 0.001**
P61	472	5.588	1	**0.018**	217.761	2	** < 0.001**	316.394	12	** < 0.001**	10.797	2	**0.005**
P65	472	3.417	1	0.065	173.585	2	** < 0.001**	291.242	12	** < 0.001**	2.741	2	0.254
P67	472	13.14	1	** < 0.001**	109.081	2	** < 0.001**	232.21	12	** < 0.001**	2.276	2	0.321
P69	378	49.603	1	** < 0.001**	157.805	2	** < 0.001**	213.855	12	** < 0.001**	14.589	2	**0.001**
P81	378	9.686	1	**0.002**	154.054	2	** < 0.001**	231.249	12	** < 0.001**	20.619	2	** < 0.001**
P85	378	0.73	1	0.393	121.996	2	** < 0.001**	204.211	12	** < 0.001**	18.978	2	** < 0.001**
P89	378	23.849	1	** < 0.001**	62.341	2	** < 0.001**	141.686	12	** < 0.001**	28.413	2	** < 0.001**

Plant architecture: Q1 to Q11; flower, fruit, and yield: Q12 to Q35; and phenology: P8 to P89. Bold numbers denote significant *p*-values.

**Table 2:** Kruskal–Wallis *H* tests for the interactions between treatment (open field and greenhouse) and species, landrace, or the climatic background.

Trait	N	T × spp			T × landrace			T × climatic background		
		*H*	d.f	*p*-value	*H*	d.f	*p*-value	*H*	d.f	*p*-value
Q1	430	38.932	5	** < 0.001**	133.031	24	** < 0.001**	50.600	5	** < 0.001**
Q2	430	181.601	5	** < 0.001**	267.972	24	** < 0.001**	29.337	5	** < 0.001**
Q4	406	102.337	4	** < 0.001**	136.708	22	** < 0.001**	23.827	5	** < 0.001**
Q6	403	43.483	5	** < 0.001**	163.548	22	** < 0.001**	52.618	5	** < 0.001**
Q7	403	56.589	5	** < 0.001**	165.06	22	** < 0.001**	55.271	5	** < 0.001**
Q8	403	100.955	5	** < 0.001**	203.247	22	** < 0.001**	107.458	5	** < 0.001**
Q9	403	94.719	5	** < 0.001**	182.826	22	** < 0.001**	44.544	5	** < 0.001**
Q10	403	45.586	5	** < 0.001**	184.274	22	** < 0.001**	106.757	5	** < 0.001**
Q11	403	198.524	5	** < 0.001**	280.26	22	** < 0.001**	153.362	5	** < 0.001**
Q12	473	148.684	5	** < 0.001**	367.742	24	** < 0.001**	129.496	5	** < 0.001**
Q15	225	75.731	3	** < 0.001**	127.491	17	** < 0.001**	18.072	5	**0.003**
Q16	280	147.241	5	** < 0.001**	232.325	20	** < 0.001**	32.914	5	** < 0.001**
Q17	280	156.473	5	** < 0.001**	232.984	20	** < 0.001**	35.651	5	** < 0.001**
Q18	280	177.719	5	** < 0.001**	246.991	20	** < 0.001**	123.577	5	** < 0.001**
Q19	280	168.768	5	** < 0.001**	239.138	20	** < 0.001**	80.645	5	** < 0.001**
Q20	280	143.358	5	** < 0.001**	237.256	20	** < 0.001**	43.121	5	** < 0.001**
Q21	529	162.734	5	** < 0.001**	340.922	24	** < 0.001**	134.117	5	** < 0.001**
Q22	457	105.248	5	** < 0.001**	198.427	21	** < 0.001**	40.954	5	** < 0.001**
Q23	328	175.081	5	** < 0.001**	238.495	19	** < 0.001**	10.290	5	0.067
Q24	328	169.425	5	** < 0.001**	282.454	19	** < 0.001**	102.933	5	** < 0.001**
Q25	328	88.077	5	** < 0.001**	246.941	19	** < 0.001**	54.114	5	** < 0.001**
Q26	328	204.7	5	** < 0.001**	273.904	19	** < 0.001**	62.947	5	** < 0.001**
Q27	530	119.336	5	** < 0.001**	329.128	24	** < 0.001**	139.674	5	** < 0.001**
Q28	328	194.009	5	** < 0.001**	278.569	19	** < 0.001**	127.166	5	** < 0.001**
Q29	328	204.833	5	** < 0.001**	306.412	19	** < 0.001**	142.024	5	** < 0.001**
Q30	328	182.736	5	** < 0.001**	275.05	19	** < 0.001**	13.363	5	**0.020**
Q31	328	109.329	5	** < 0.001**	316.88	19	** < 0.001**	76.337	5	** < 0.001**
Q32	528	154.954	5	** < 0.001**	336.062	24	** < 0.001**	110.762	5	** < 0.001**
Q33	528	183.188	5	** < 0.001**	342.292	24	** < 0.001**	147.065	5	** < 0.001**
Q34	528	189.336	5	** < 0.001**	348.467	24	** < 0.001**	153.192	5	** < 0.001**
Q35	528	183.57	5	** < 0.001**	354.885	24	** < 0.001**	171.424	5	** < 0.001**
P8	530	60.815	5	** < 0.001**	192.665	24	** < 0.001**	53.910	5	** < 0.001**
P9	530	108.034	5	** < 0.001**	269.959	24	** < 0.001**	78.202	5	** < 0.001**
P10	530	56.265	5	** < 0.001**	240.29	24	** < 0.001**	70.998	5	** < 0.001**
P12	530	41.546	5	** < 0.001**	256.043	24	** < 0.001**	47.527	5	** < 0.001**
P13	530	74.248	5	** < 0.001**	231.139	24	** < 0.001**	121.487	5	** < 0.001**
P19	522	256.817	5	** < 0.001**	340.29	24	** < 0.001**	134.174	5	** < 0.001**
P21	522	197.299	5	** < 0.001**	243.985	24	** < 0.001**	190.502	5	** < 0.001**
P51	486	257.635	5	** < 0.001**	377.68	24	** < 0.001**	51.606	5	** < 0.001**
P55	485	272.531	5	** < 0.001**	380.144	24	** < 0.001**	58.656	5	** < 0.001**
P59	481	277.242	5	** < 0.001**	376.407	24	** < 0.001**	64.704	5	** < 0.001**
P61	472	229.464	5	** < 0.001**	349.702	24	** < 0.001**	20.228	5	**0.001**
P65	472	177.119	5	** < 0.001**	331.746	24	** < 0.001**	16.090	5	**0.007**
P67	472	121.977	5	** < 0.001**	297.847	24	** < 0.001**	23.375	5	** < 0.001**
P69	378	223.418	5	** < 0.001**	295.365	23	** < 0.001**	68.309	5	** < 0.001**
P81	378	171.43	5	** < 0.001**	262.997	23	** < 0.001**	33.643	5	** < 0.001**
P85	378	126.518	5	** < 0.001**	229.667	23	** < 0.001**	22.890	5	** < 0.001**
P89	378	88.751	5	** < 0.001**	201.079	23	** < 0.001**	56.909	5	** < 0.001**

### I. Plant architecture

Plants under high temperatures and low humidity in the greenhouse exhibited significant higher overall mean rank values than field plants for stem diameter, the degree of branch orientation, composite sheet length and width, and the terminal leaflet length. The size of the angle of the base of the terminal leaflet, however, was bigger in the field (Supporting Tables S3 and Table 1). There were overall significant differences for species and landrace for all studied characters (Table 1). The Kruskal–Wallis analyses of the interactions between treatment (open field vs greenhouse conditions) and species, climatic background, and landrace were significant for all the traits (*p*-value < 0.001; Table 2).

Post hoc pairwise comparisons for treatment × species interaction (Table 3), found that *P. vulgaris* plants produced significant higher mean rank values for branch orientation angle in the greenhouse than in the field (median values: 140.00° vs 133.33°). Similarly, *P. lunatus* plants exhibited significant higher values in the greenhouse for composite sheet length and width and terminal leaflet width (median values: 238.28, 209.95 and 115.26 mm, respectively) than in the field (median values: 208.34, 169.27 and 93.76 mm, respectively); but the terminal leaflet length performed better in the field compared to greenhouse (medians: 62.36 and 52.02 mm). Post hoc pairwise comparisons for treatment × climatic background highlighted that cold background landraces had higher values for branch orientation angle, composite leaf length and width, and terminal leaflet length in the greenhouse than in the field. Cold background landraces produced wider terminal leaflet widths in the greenhouse while warm background landraces did it in the field (Table 3). Post hoc analysis for the treatment × landrace (Table 4) found that *P. lunatus* had higher mean rank values in the greenhouse than in the open field for composite sheet length (238.28 vs 208.34 mm) and width (209.95 vs 169.27 mm), and terminal leaflet length (115.26 vs 93.76 mm). *P. vulgaris* landrace 8 failed to grow in the field. Plant architectural traits were only affected in one *P. vulgaris* (8) and the *P. lunatus* (13) landraces. *P. vulgaris* landrace 1, from cold background, performed better in the field than in the greenhouse. Landrace 8, from cold background as well; however, performed better in the greenhouse than in the field, suggesting a wrong identification of its real origin.

**Table 3:**
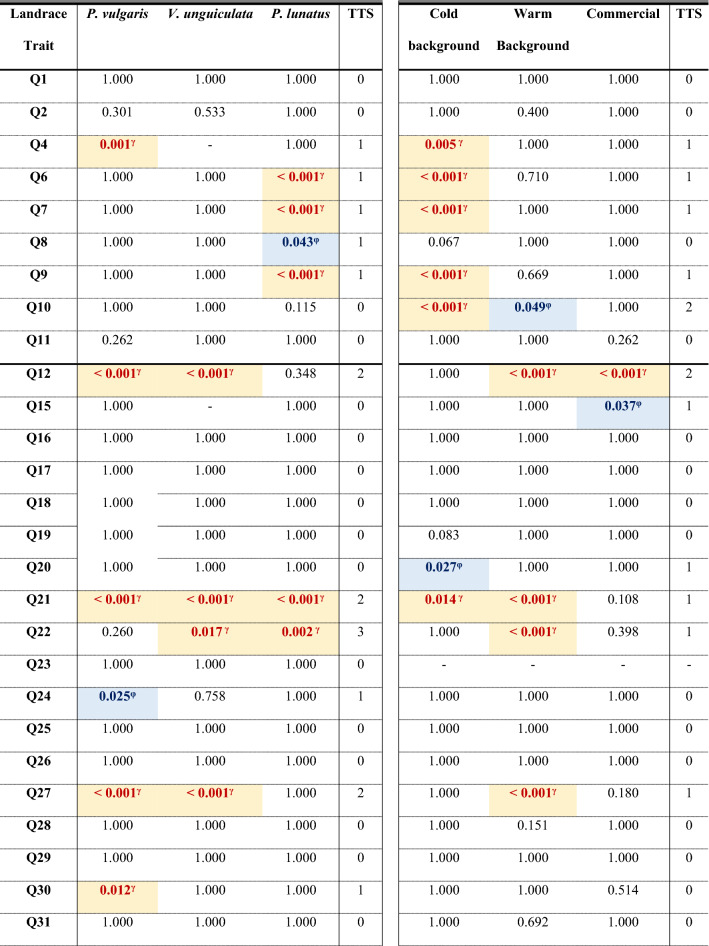

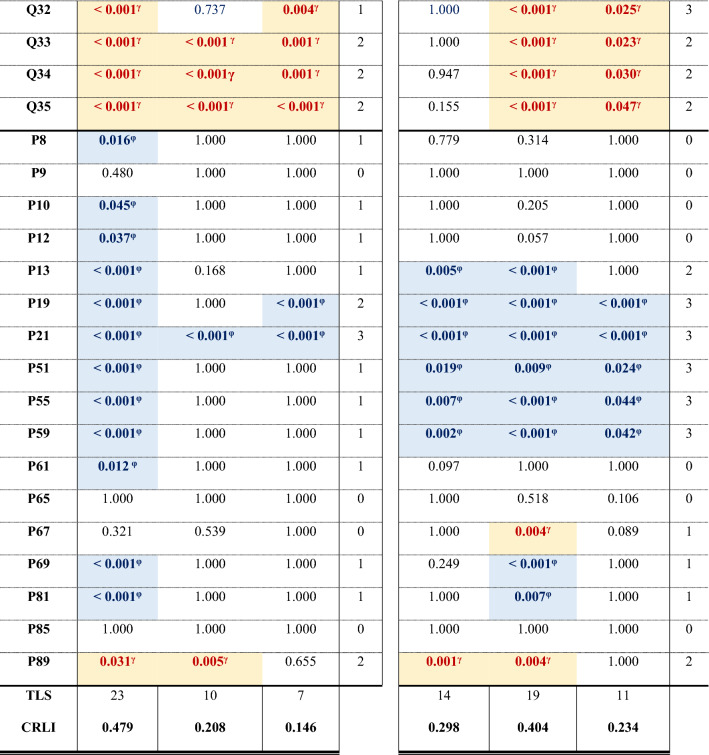
Effects of treatment on each trait for the three species and the climatic background of the landraces.

Values are the *p*-values corrected after Bonferroni from the post hoc tests to analyse pairwise comparisons. TTS, total trait significance, is the number of significant pairwise comparisons in each row; considering that lack of data sometimes implies that the landrace failed to exhibit the character. TLS, total landrace significance, is the number of significant pairwise comparisons per column; and CRLI, climate resilience landrace index, is TLS/(# of traits). Values in bold denote significant *p*-values. In addition, we use φ as super index and a pale blue cell filling to designate either when the median of the character was statistically higher in the open field than in the greenhouse or when the plants did not produce the trait in the greenhouse treatment. Then, we used γ and a pale-yellow cell filling to designate either when the value is statistically higher in the greenhouse treatment than in the open field or when the plants did not exhibit the trait in the field. – denotes missing values. Character Q23 in climate background columns has no values because the Kruskal–Wallis *H* test was not significant.

**Table 4:**
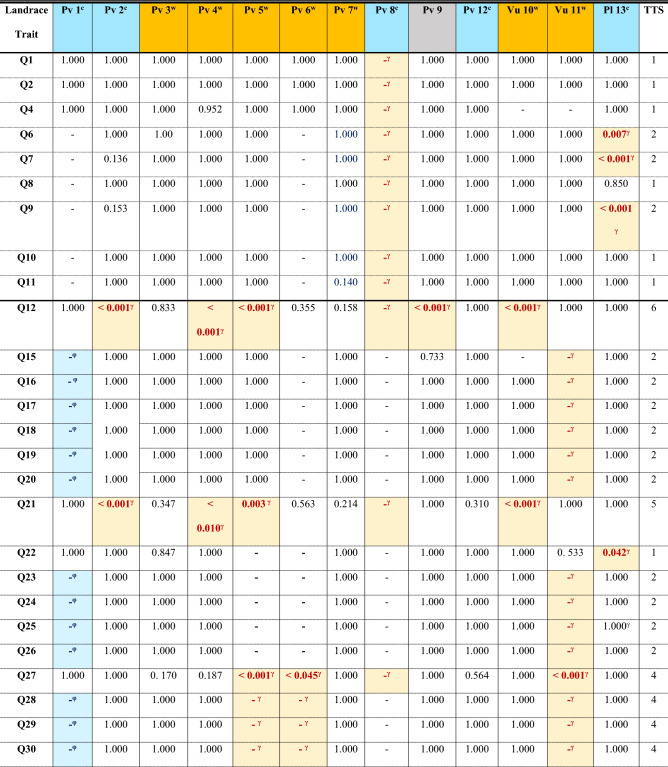

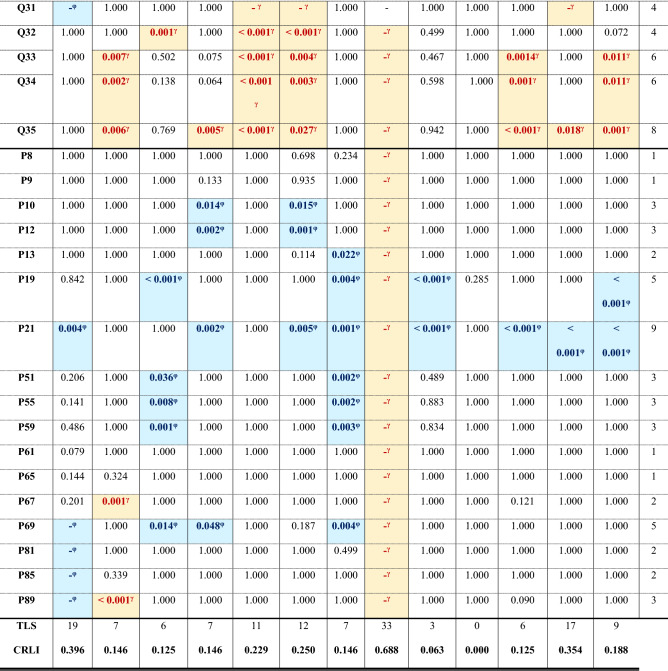
Differences within the same landrace grown in the open field and the greenhouse for each trait.

Values are the *p*-values corrected after Bonferroni from the post hoc tests to analyse pairwise comparisons. Pv: *P. vulgaris*; Vu: *V. unguiculata*; Pl: *P. lunatus*. ^c^ designates cold background (blue cell filling) and ^w^ warm background (orange cell filling) landraces; cultivar 9 was of commercial origin (grey filling). TTS, total trait significance, is the number of significant pairwise comparisons in each row; considering that lack of data sometimes implies that the landrace failed to exhibit the character. TLS, total landrace significance, is the number of significant pairwise comparisons per column; and CRLI, climate resilience landrace index, is TLS/(# of traits). Values in bold denote significant *p*-values. *φ* and a pale blue cell filling designate either when the median of the character was statistically higher in the open field than in the greenhouse or when the plants did not exhibit the trait in the greenhouse treatment. *γ* and a pale-yellow cell filling designates either when the value is statistically higher in the greenhouse treatment than in the open field or when the plants did not exhibit the trait in the field. – denotes lack of data.

### II. Flower and fruit characteristics and yield

Greenhouse plants exhibited significant higher overall mean rank values than field plants for the total number of flowers per plant (medians: 57 vs 11 flowers), the number of pods per plant (medians: 34.5 vs 2.0 pods), the number of pods per infructescence (medians: 1.6 vs 1.4 pods), the number of grains per pod (medians: 4.2 vs 0.0 grains), the dry weight of 100 seeds (medians: 37.46 vs 0.0 g), the gross weight of seeds per plant (medians: 24.92 vs 0.0 g), the net weight of seeds per plant (medians: 24.01 vs 0.0 g), and the number of seeds (medians: 60.0 vs 0.0). On the contrary, the sheath width was larger in the field (12.48 mm) than in the greenhouse (11.01 mm; see Supporting Table S4 and Table 1). There were overall significant differences for species and landrace for all characters (Supporting Table S4 and Table 1). For the climatic background of the landraces, all characters exhibited significant differences except for the peduncle length, the number of pods per plant and the number of pods per infructescence.

The Kruskal–Wallis analyses of the treatment × species, treatment × climatic background (except for sheath length) and treatment × landrace interactions were significant for all the traits (Table 2). Post hoc pairwise comparisons for treatment × species interaction found that *P. vulgaris*, *V. unguiculata* and *P. lunatus* produced higher mean rank values in the greenhouse than in the field (Table 3) for: the number of pods per plant (median values: 31.0, 20.5 and 96.0, respectively vs 3.5, 0.0 and 47.5 pods), the gross weight of seeds per plant (medians: 24.04, 17.11 and 251.90 vs 0.00, 0.00 and 112.12 g, respectively), the net weight of seeds per plant (medians: 22.32, 15.25 and 238.78 vs 0.00, 0.00 and 104.80 g, respectively), and the total number of seeds (medians: 36.5,93.5 and 205 vs 0.0, 0.0 and 93.5 seeds, respectively). The sheath width was significantly higher in the field than in the greenhouse (medians: 12.8 and 11.4 mm, respectively), and the scar length mean ranks were higher in the greenhouse than in the field only for *P. vulgaris*. Then, *P. vulgaris* and *V. unguiculata*, produced higher mean rank values for the following characters in the greenhouse than in the field (Table 3) for the number of flowers per plant (medians: 48.0 and 28.0 vs 11 and 7.5 flowers, respectively) and the number of grains per pod (medians: 4.2 and 10.0 vs 0.0 grains, respectively). Similarly, *P. vulgaris* and *P. lunatus* exhibited higher mean rank values in the greenhouse than in the field for the weight of 100 seeds (medians: 40.51 and 116.39 vs 0.00 and 109.43 g, respectively).

In the treatment × climatic background, post hoc pairwise comparisons (Table 3) found significant higher mean rank values in landraces from cold background growing in the field than in the greenhouse for chalice length. Warm background landraces produced higher mean rank values in the greenhouse for the number of flowers per plant, the number of pods per plant and infructescence, the number of grains per pod, the 100 seeds weight, the gross and net weight of seeds per plant and the number of seeds. The commercial cultivar exhibited higher median peduncle length in the field than in the greenhouse, and higher median values in the greenhouse than in the field for the number of flowers per plant, the 100 seeds weight, the gross and net weight of seeds per plant and the number of seeds.

In the treatment × landrace, post hoc analysis (Table 4) found that the number of flowers per plant was significantly higher in *P. vulgaris* landraces 2, 4, 5, 8 and 9, and in *V. unguiculata* 10. Analogously, more pods per plant were produced in the greenhouse for *P. vulgaris* landraces 2, 4, 5 and 8, and in *V. unguiculata* 10. The total number of seeds produced in the greenhouse was higher than in the field for *P. vulgaris* landraces 2, 4, 5, 6 and 8, both *V. unguiculata* 10 and 11, and *P. lunatus* 13. *P. vulgaris* 3 produced higher mean rank values for the 100 seeds weight in the greenhouse than in the field. *P. vulgaris* landraces 5 and 6 (both from warm background), however, failed to produce enough flowers, pods, or seeds. Landrace 1, from cold background, failed to prosper in the greenhouse, and landrace 8 did not grow in the field. Otherwise, all other significant values highlighted the positive effects of warmer conditions compared to the field acting on flower, fruit, and yield characteristics (Table 4).

### III. Phenology

**Figure 1:**
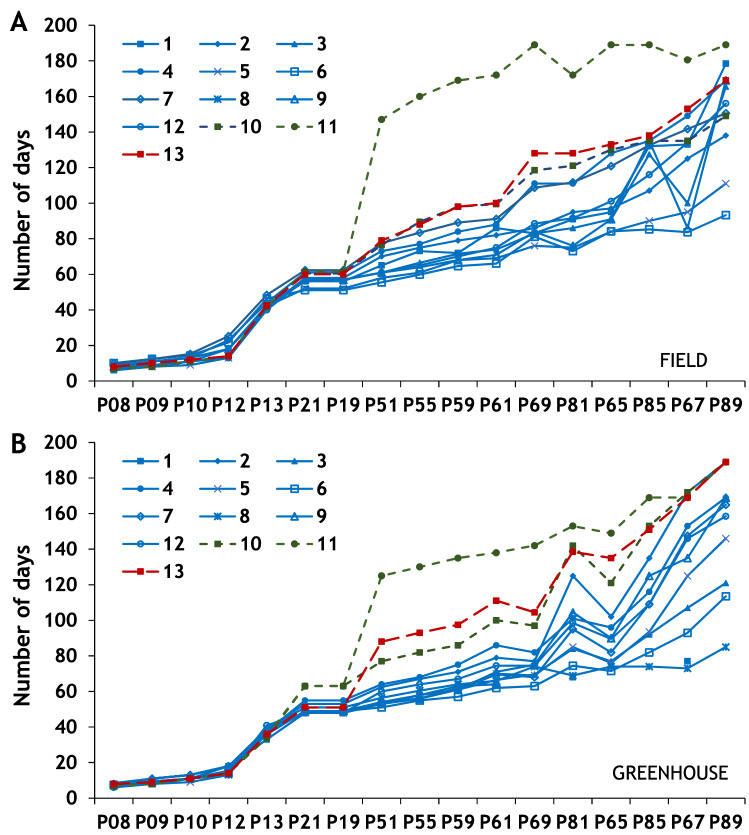
Phenological stages in chronological order. **(A)** Development of the 13 landraces in the open field treatment; **(B)** development of the 13 landraces in the greenhouse.

The treatment had significant overall effects for all the studied characters except for the emergence of hypocotyl, the full flowering when the 50% of the flowers are open, and the 50% of pods ripe (Supporting Tables S5 and Table 1; Fig. 1). Moreover, growing in the open field under colder and more humid conditions than in the greenhouse, delayed the development of the characters except for the finishing of the flowering, which was delayed in the greenhouse (medians: 144 and 123 days, respectively), and the time when pods are fully ripe (medians: 169 and 146 days, respectively). All the three species showed significant differences in mean ranks for all their phenological characters except for the first side shoot visible, while for landrace, there were significant differences for all the characters (Table 1). However, the climatic background of the landrace had no effects on the first side shoot visible, the full flowering and the end of flowering.

The Kruskal–Wallis analyses of the treatment × species, treatment × climatic background and treatment × landrace were significant for all traits (Table 2). Post hoc analysis for treatment × species (Table 3) found that *V. unguiculata* developed the first phenological traits (P08–P12) faster than *P. vulgaris* and *P. lunatus*. However, *V. unguiculata* was the slowest species producing the flowering period (median: 121–169 days; *P. vulgaris* 71–116 days; *P. lunatus* 107–169 days), and produced more seeds per pod (9 seeds) than *P. vulgaris* (3.6 seeds) or *P. lunatus* (2.1 seeds), for other yield related traits produced lower values than the *Phaseolus sp.* The presence of the first shoot visible had higher mean rank values (appearance of the character delayed in time) in the field than in the greenhouse for all the species. In *P. vulgaris*, all significant comparisons confirmed that field conditions delayed the development of such traits, except for the fully ripe pods, which was delayed in the greenhouse for *P. vulgaris* and *V. unguiculata* (medians: 156.0 and 189.0 vs 136.5 and 149.0 days, respectively). In addition, *P. lunatus* produced nine or more leaves unfolded later in the field than in the greenhouse (medians: 60 vs 51 days).

Post hoc analysis for the treatment × climatic background (Table 3), found significant differences in mean rank values for the development of nine or more leaves unfolded, the first side shoot visible, the first flower buds visible and enlarged, and the first petals visible independently of the background origin of the landraces, which happened later in the field than in the greenhouse. In landraces from cold or warm background, the third true leaf developed later in the field compared to greenhouse but the fully ripe pods developed later in the greenhouse (Table 3). In warm background landraces, the flowering finishing was delayed in the greenhouse but the presence of first pods visible and the occurrence of 10% of pods ripe was delayed in the field.

Post hoc analysis for the treatment × landrace (Table 4) found that the number of pods per plant exhibited significant higher values in the field than in the greenhouse for *P. vulgaris* landraces 1, 4, 6, 7 and 9, *P. lunatus* 13 and both *V. unguiculatus* 10 and 11. *P. vulgaris* landrace 2 finished the flowering and the fully ripe pods later in the greenhouse than in the field; and landrace 8 failed to grow under field conditions. The development of nine or more leaves for *P. vulgaris* landraces 3, 7 and 9 and *P. lunatus*, and the end of flowering for *P. vulgaris* landraces 1, 3, 4 and 7 were delayed in the field. Other significant differences in character expression had higher values in the field (Table 4).

### IV. Climate resilience landrace index and clustering

Across all landraces, the following morphological and reproductive characters were the strongly affected by changing the environmental conditions of the plants (when three or more landraces exhibited significant changes in the expression of the character): the number of flowers per plant, the number of pods per plant, the number grains per pod, the grain length, width and thickness, the scar length, the 100 seeds weight, the gross and net weight of seeds/plant and the number of seeds. Phenologically, the most affected traits were: cotyledons completely unfolded, two full leaves unfolded, the unfolding of nine or more leaves, the emergence of the first shoot, first flower buds visible and enlarged, first petals visible, the end of flowering and the pods fully ripe.

The climate resilience landrace index (CRLI, Table 4) found that *P. vulgaris* landrace 8 (0.688) was highly susceptible, and landraces 1 (0.396) and 6 (0.250) and *V. unguiculata* landrace 11 (0.354) were very susceptible to changes in their environmental conditions. *P. vulgaris* landrace 12 and the commercial variety 9 were the most resilient to environmental conditions (0.000 and 0.063, respectively). When using this index for species, *P. vulgaris* was the most susceptible (0.479) and *P. lunatus* the most resilient (0.146) to treatment. Warm background landraces were more prone to accumulate significant differences in their traits (0.404) compared to cold background (0.298) or the commercial cultivar (0.234).

**Figure 2:**
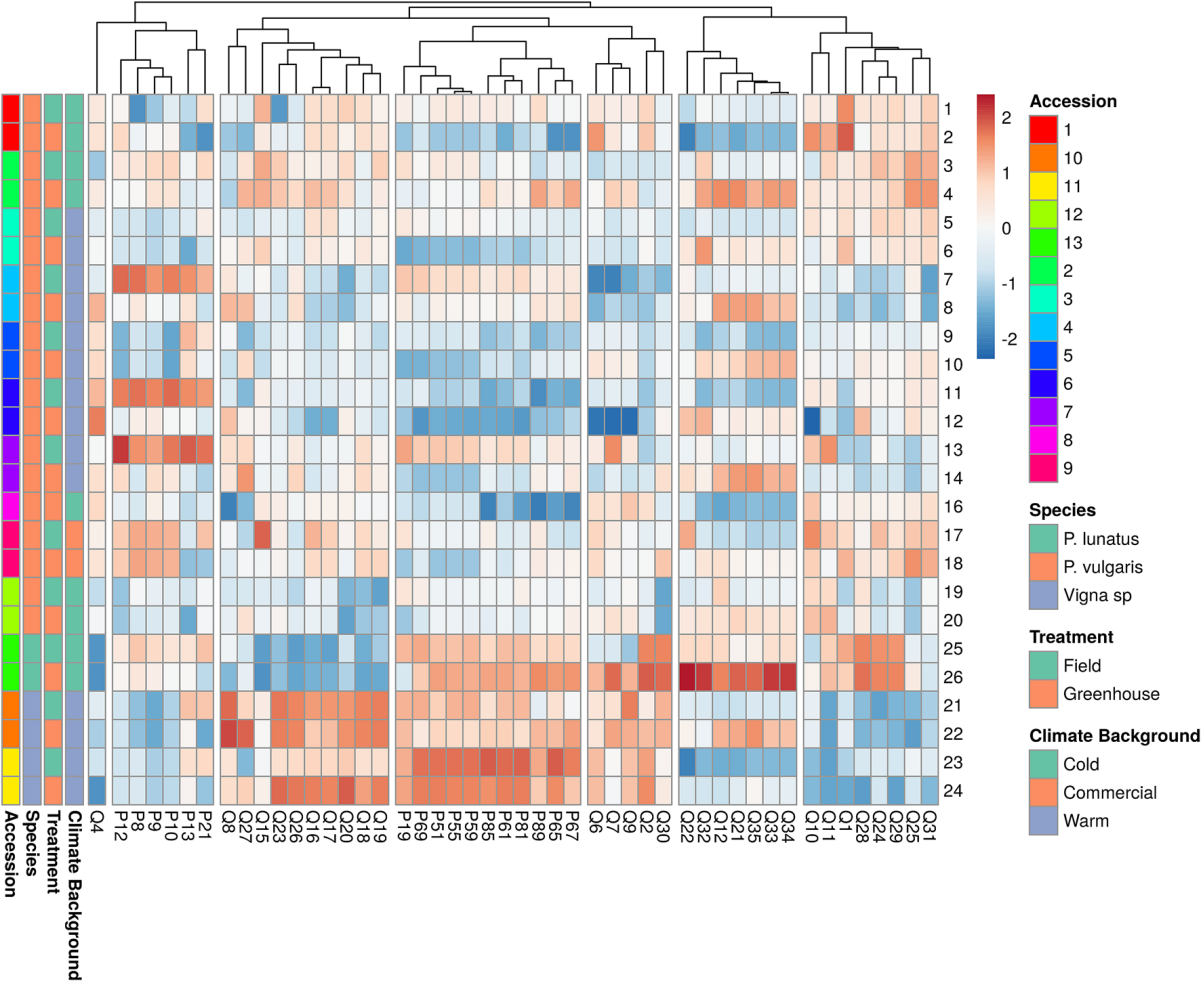
Heatmap of the studied landraces based on the mean rank values for all morphological and phenological characters. Hierarchical clustering of the heatmap for all the studied characters (columns) in the 13 landraces (rows). Columns are clustered using Euclidean distance and complete linkage. Then, we stablished seven groups of characters after the results of the PCA and the parallel analysis that suggested seven factors.

The clustering of the mean ranks for the 48 characters expressed by the 12 landraces in both treatments produced a heatmap (Fig. 2) that identified the groups of morphological and phenological characters based on the components of the PCA, thus highlighting the differences induced by the treatment. All phenological characters were grouped in two clusters.

## Discussion

Coping with climate change impact on crops might strongly benefit from those landraces that had been bred locally^34^. The analysis of the phenotypic plasticity of the intra- and interspecific diversity of local bean landraces from the Andes of South Ecuador found that climate change effects might accelerate the loss of certain landraces while having benign or none effects on others.

Changing environmental conditions had a direct impact on more than half (60.4%) of the 48 analysed characters. The most informative results; however, are those related to the interaction of treatment with species, climatic background, and landraces. The treatment affected the three species differently. Moreover, some characters exhibited higher levels of variation across species and landraces than others in response to the treatment: the number of flowers and seeds per plant, the gross and net weight of seeds and the emergence of the first side shoot visible were the most plastic traits across species. In addition, we found three *P. vulgaris* landraces from cold background with very different behaviours: landrace 1 was very susceptible to warmer conditions; landrace 8 performed very poorly in general, although better under warmer conditions than in the field; and landrace 12 was the most resilient material to any environmental change. Thus, we identified the former two landraces as critical targets for conservation and the latter as a potential landrace to replace locally those beans crops more affected by environmental conditions. Similar results can be found in maize landraces from Chiapas, where climate change may have strong impact on farmers that depend on locally adapted landraces^35^. Moreover, we identified suitable plant material for greenhouse cultivation at high altitudes in Andean communities. The later have three nonexclusive advantages that promote sustainable agriculture: (1) boosting natural soil conservation (maintenance of covered soils) and fertilisation with legume crops during fallow or crop rotation periods, either in the field or in the greenhouse; (2) in situ conservation of landraces; and (3) extra benefits for small farmers (production of fodder, food surplus production, and extra income in markets).

*P. lunatus* was the more tolerant species and *P. vulgaris* the more susceptible to changes in environmental conditions, confirming previous findings^14^. While warm conditions affected one architectural trait in *P. vulgaris* and four traits in *P. lunatus*, it had not effects on *V. unguiculata*.

The major effects of the treatment on characters related to reproduction and yield happened in *P. vulgaris* and *V. unguiculata*, and most of the phenological differences were found in *P. vulgaris*. Cold background landraces produced larger chalice lengths, thus suggesting either a potential adaption of cold background landraces to cold environmental conditions, related to the protection of the reproductive organs or facilitating the accumulation of heat trapped within the flower structure with micro greenhouse-like effects^36^. Warm conditions boosted seed and yield traits in the warm background and the commercial cultivars, supporting that warm background landraces are better adapted to temperatures higher than in the field and that the commercial cultivar performs better in warmer conditions. All the phenological characters involving flower development were delayed in the field, independently of the climatic background of the landrace. The fully ripe pods happened later in the greenhouse than in the field for both cold and warm background landraces, suggesting that warmer conditions might impose higher demands of water and storage of products in the grains, thus, prolonging the time for maturation.

We identified, through using the CRLI, three *P. vulgaris* landraces that are sensible (1, 6 and 8) and one landrace more resilient (12) to environmental changes. This could lead to developing specific in situ and ex situ conservation strategies. While many landraces are grown locally by the farmers, others might be present as the result of seed exchange activities (e.g., seed fairs) or migration of people. Flower and fruit characteristics and yield were improved in the greenhouse conditions, except for *P. vulgaris* landrace 1. Colder conditions delayed the emergence of many phenological traits except for the end of flowering of *P. vulgaris* landrace 2, from cold background, supporting its cold climatic background origin.

The first side shoot visible was the most widely affected trait by the environmental changes across landraces, highlighting the overall delay in vegetative growth in the field, and suggesting potential trade-offs in resource allocation. Warmer conditions increased stem diameter, the composite sheet length and width, and the terminal leaflet length. The latter might highlight both that some landraces had higher optimal temperatures of growth that the ones faced in the field, and that the temperatures reached in the greenhouse (day, night, their difference, and soil temperatures) are not high enough to promote plant respiration over photosynthesis. The former hypothesis seems plausible when interpreting the results after analysing the interaction between treatment and climatic background of the landraces. Moreover, that some cold background landraces performed better at higher temperatures than warm background landraces suggests that the later have reached an adaptive plateau that might not be surpassed. A potential explanation is that cold background materials could have a warm background origin, thus suggesting the later migration of the landraces by farmers through seed exchange and commercialization.

As expected, we found differences in performance and phenology among species supporting previous findings^37^. The values found for *P. vulgaris* traits, are within the expected variation among previously studied landraces and cultivars^38^. However, differences in the timing of flowering traits among *P. vulgaris* landraces might alter gene flow and promote reproductive isolation; thus, affecting their evolutionary dynamics^39^. *P. lunatus* was the most productive species; moreover, *P. lunatus* might be considered as a potential species to grow in greenhouses and replace other crops, economically less attractive, thus, adding a value to food security and soil fertilisation.

The greenhouse conditions had a positive effect on the production of flowers and on many important components related to pods and yield. We did not expect these positive effects after previous findings in *P. vulgaris*, where an increase of temperature affected negatively the number of seeds per pod, seed size and yield^40,41^. Moreover, the higher production of flowers and, thus, seeds under greenhouse conditions might be partially explained by the protection against winds that affect flower survival in the open field. Previous work; however, found that very high temperatures reduced the values for many reproductive traits^33^; but the latter was tested in a climate chamber a range of temperatures much higher than the ones reached in our greenhouse. The lower median values for the dry weight of the seeds in the open field; however, might be due to trade-offs between investment in reproduction and resistance against harsh abiotic or biotic conditions. Furthermore, the positive relation between temperature and seed weight was previously observed in *V. unguiculata*^42^. It is noteworthy to mention, that our results highlighted the importance of the climatic background from where the landraces come from as a fixed factor because it allowed us to better interpret the actual impact of temperature on crops and their landraces. Thus, we found that cold background plants produced the highest number of flowers and seeds and that warm background landraces performed better in the greenhouse than in the field. The behaviour of the commercial cultivar, like cold background landraces, suggests its potential cold background origin.

Warmer conditions accelerated the phenological development of plants for many of the analysed traits, although the day when the pods are fully ripped, was delayed in the greenhouse. This trend has been reported in other crop species but exhibiting a negative effect on crop yield^43,44^. Thus, global warming might be critical in several Andean crops, such as potato, which is highly sensible to high temperature^45^. Moreover, in addition to a negative impact on yield and quality, the rise of global temperatures will reduce the areas suitable for other crop species, such as coffee (*Coffea arabica*)^46^. In *P. vulgaris*, the advance of the flowering stage in the field due to warmer conditions highlighted the critical problem of flower-pollinator coupling^47,48^. Early flowering might respond to different environmental pressures and, for example, in oat Mediterranean landraces, it represents a potential mechanism to scape terminal drought^44^. However, we found that warm background landraces developed faster in the greenhouse than in the field for some characters, which might represent important adaptive characteristics (e.g., faster acquisition of energy) suggesting a fine-tuned genotype × environment interaction. Notwithstanding, most of the phenological traits developed later in the field for both the cold and warm background landraces. These results make us think of colder conditions imposing more costs to the plant, and then, architectural and reproductive traits develop slower in colder than in warmer conditions, where the latter facilitates fruit and seed maturation.

The ecological background from where the genetic material comes from (Table 3) plays a key role in the potential tolerance to high temperatures. Moreover, the hereby-proposed climate resilience landrace index (CRLI) might work as a proxy to identify landraces that are more prone to suffer the effects of climate change, such as *P. vulgaris* landrace 1, which seems to be very sensible to heat, or *P. vulgaris* landraces 6 and 8, more sensible to colder conditions than the other landraces. Moreover, this CRLI index highlighted *P. vulgaris* landrace 12 as the most resilient against environmental changes. The CRLI index, then, might be a powerful preliminary approach before identifying potential QTLs or traits for new breeds or genetic improvement, as well as a help for decision-making institutions or management tools^23^.

The hierarchical clustering of the heatmap for the 13 landraces in both treatments pointed out both that the level of expression of some characters are landrace or species specific, and that many plant morphological characters are very plastic, depending on the environmental conditions in which those traits are measured (Fig. 2). This highlights the relevance of the in situ morphological characterization of any plant population, landrace, or species. In addition, these plant genetic resources might be opportune as tools for generating resilience against climate change effects, and boost sustainable agriculture practices, strengthening food security. Moreover, the use of landraces to tackle climate challenges can fulfil specific climatic needs and might represent a source of germplasm for plant breeders^49^. Our work further emphasizes the importance of identifying the ecological antecedents of the landraces or crop populations because these data, being part of the characterization and later analysis, might become a robust tool for their conservation. Furthermore, the identification of appropriate standardised agromorphologic descriptors and phenological scales for each species might improve the use of plant passport data^50–53^. Finally, the characterization of the agromorphological and physiological profiles of a collection of local plant materials can generate resilience against other disasters in addition to climate change, such as the COVID-19 pandemic; since it has caused the shortage of seeds of commercial varieties in developing communities^54^.

## Material and methods

### Plant material, location, and cultivation

We selected nine *Phaseolus vulgaris*, one *P. lunatus* and two *V. unguiculata* landraces collected from small farmers from the Andean region of South Ecuador during 2017 and conserved in the UTPL germplasm bank, and one commercial *P. vulgaris* cultivar. All UTPL landraces were collected from sites with different climatic background conditions from Loja province (Ecuador) except the UTPL-PGR-0798 landrace, collected from El Oro province (Supporting Table S1, Supporting Fig. S1). We selected these landraces to test whether their climatic background had an effect on their performance against two different environmental conditions. Thus, depending on the altitude at which the material was found, which has a direct correlation in Ecuador with climatic conditions, we assigned each landrace to cold (above 2000 m.a.s.l.), warm (between 900 and 2000 m.a.s.l), or commercial (unknown) background. *P. vulgaris* landraces are numbered as 1–9 and 12 (UTPL germplasm bank codes: UTPL-PGR-0156, UTPL-PGR-0168, UTPL-PGR-0311, UTPL-PGR-0314, UTPL-PGR-0316, UTPL-PGR-0318, UTPL-PGR-0344, UTPL-PGR-0345 and UTPL-PGR-0798, respectively), *V. unguiculata* as 10 and 11 (UTPL-PGR-0313 and UTPL-PGR-0317), and *P. lunatus* as 13 (UTPL-PGR-0230). Cold background landraces are 1, 2, 8, 12and 13; the warm background landraces are 3–7, 10 and 11. The number 9 is the commercial cultivar.

We started the experiments on July 19th 2018, and lasted until the end of March 2019 at a field located in the Universidad Técnica Particular de Loja (UTPL, Loja, Ecuador. Coordinates: 4°0′1.59" S and 79°10′48.46" W). The site has an altitude of 2160 m.a.s.l., an average minimum/maximum temperature across the year of 12.9 °C and 22.6 °C (annual mean around 16.7 °C), an average annual precipitation of 780 mm and a relative humidity of 81.07%. In the same location, we settled a greenhouse with a monthly average temperature of 23.5 °C (21 °C minimum and 26 °C maximum) and relative humidity of 57.62%. Loja corresponds to the low dry montane forest (bs-MB) ecological formation^55^. Then, we first seeded an excess of seed for each landrace in a tunnel nursery at 25 °C and 45% relative humidity for 18 days. For each landrace we transplanted 72 healthy seedlings in each of the two environments (open field and greenhouse), at 50 cm between plants and one meter between rows, and drip irrigation in the greenhouse (up to twice a week to avoid hydric stress). Each environment represented one climatic condition. According to soil analysis performed in the Agrocalidad laboratory (Agencia de Regulación y Control Fito y Zoosanitario), a fertilization program was applied with mineral fertilization based on 12:36:12 (N-P_2_O_5_-K_2_O) as basic fertilization on both environments.

### Morphoagronomic characterisation

We quantified the development and production of each plant by using 49 descriptors based on Bioversity International (http://www.bioversityinternational.org/publications), while integrating the particularity of each studied species: *P. vulgaris*^50^, *P. lunatus*^51^ and *V. unguiculata*^52^, and evaluated 32 morphological characters of plant architecture, inflorescence and fruit characters, and yield. Then, we used the BBCH codifications^53^ to register 17 phenological stages (Supporting Table S2). Plant architecture characters: stem length (Q1), stem diameter (Q2), number of main branches (Q3), branch orientation (Q4), composite sheet length (Q6), composite sheet width (Q7), apex angle of terminal leaflet (Q8), terminal leaflet length (Q9), terminal leaflet width (Q10) and angle of the base of the terminal leaflet (Q11). Flower and fruit characteristics and yield characters: number of flowers per plant (Q12), peduncle length (Q15), left wing length (Q16), right wing length (Q17), banner length (Q18), style length (Q19), chalice length (Q20), number of pods per plant (Q21), number of pods per infructescence (Q22), sheath length (Q23), sheath width (Q24), sheath thickness (Q25), number of loculi per pod (Q26), number of grains per pod (Q27), grain length (Q28), grain width (Q29), scar length (Q30), grain thickness (Q31), 100 seed weight (Q32), gross weight of seeds/plant (Q33), net weight of seed/plant (Q34) and number of seeds (Q35). Phenological characters: Hypocotyl reaches the soil surface (P08), hypocotyl with cotyledons break through soil surface (P09), cotyledons completely unfolded (P10), 2 full leaves (P12), 3rd true leaf (P13), 9 or more leaves unfolded (P19), first side shoot visible (P21), first flower buds visible (P51), first flower buds enlarged (P55), first petals visible, flowers still closed (P59), beginning of flowering (P61), full flowering: 50% of flowers open (P65), flowering finishing (P67), end of flowering (P69), 10% of pods ripe (P81), 50% of pods ripe (P85) and fully ripe pods (P89).For most descriptors, we gathered up measures directly in the field, while other characters were measured in the laboratory by using the image-processing tool ImageJ^56^ (Supporting Table S2).

### Data analysis

We calculated an explorative correlation matrix between all pairs of the 49 variables and found that Q3 was uncorrelated with any other factor (all values below 0.3). All other 48 variables accomplished the assumptions for the PCA analysis. The Kaiser–Meyer–Olkin Measure of Sampling Adequacy (KMO) produced a meritorious value of 0.897, after Kaiser’s evaluation^57^. Moreover, the Bartlett's Test of Sphericity strongly supported that our data is suitable for a factor reduction technique (*χ*^2^ = 15,722.84, df = 1128; *p* < 0.001). Overall, the extracted communalities represented well our variables, with values between 0.500 and 0.966, except for Q1 (0.401), Q4 (0.431) and Q8 (0.336). Eight components had eigenvalue values above 1.000 accounting for a cumulative 80.230% of the variability of our original 48 variables. To know how many factors retain we conducted a parallel analysis^58^ that suggested seven factors (77.648%).

We analysed for differences in plant performance between both climatic conditions (open field vs greenhouse), among species *(P. vulgaris*, *P. lunatus* and *V. unguiculata*), the 13 landraces, the climatic background (cold, warm, and commercial), and for the interactions between treatment × species, treatment × landrace, and treatment × climatic background using the nonparametric independent samples Kruskal–Wallis test, because the data did not meet the assumptions of the ANOVA. We further conducted post-hoc pairwise comparisons adjusted after Bonferroni. All statistical analyses were conducted in IBM® SPSS® Statistics v. 24.

The information gathered up in the post-hoc pairwise comparisons about each landrace performance in both climatic conditions allowed us to create and calculate the climate resilience landrace index (CRLI). First, we quantified how many characters exhibited significant post hoc *p*-values for each of the 13 landraces (total landrace significance, TLS), and divide each value by the number of traits investigated (48 morphological and phenological traits). This index highlights which landraces are more likely to be affected by different aspects of the climate change effects and, thus, will provide important information about both conservation and agricultural strategies to build up resilience against climate change effects. The closer the index is to zero the lower the impact on the landrace, and the maximum impact is one. Thus, we suggest three categories to interpret these indexes: (1) weak or none effects (strong resilience) for indexes values between 0.00 and 0.33; (2) moderate effects for values between 0.33 and 0.66; and (3) strong or drastic effects (e.g., highly susceptible landraces) for values between 0.66 and 1.00.

We conducted a Principal Component Analysis (PCA) and clustering of the 13 landraces based on the mean values of the agromorphological characters using the ClustVis webtool^59^. During the pre-processing of the data, we applied unit variance scaling as variance normalization method because we are analysing variables of different units and intensity ranges. Afterward, we followed the Nipals (Nonlinear Iterative Partial Least Squares) PCA method. In order to understanding the effects of the treatment (open field vs greenhouse) on agromorphological and phenological variation better, we further constructed a clustered heatmap with the 13 landraces in both environmental conditions. We produced the heatmap using Euclidean distance and complete linkage methods.

### Ethics declaration

Experimental research and the field study on plants complied with Ecuadorian research normative (Ley Orgánica de Agrobiodiversidad, Semillas y Fomento de la Agricultura Sustentable, 2017).

## Supplementary Information

Supplementary Information.
