# Supplementary material for: Heterogeneous effects of climatic conditions on Andean bean landraces and cowpeas highlight alternatives for crop management and conservation
Source: Sci Rep. 2022 Apr 21;12:6586. doi: 10.1038/s41598-022-10277-x (PMC9022739; doi:10.1038/s41598-022-10277-x)
Supplement: Supplementary file 1 — Supplementary Information. [file 41598_2022_10277_MOESM1_ESM.docx]

**Heterogeneous effects of climatic conditions on Andean bean landraces and cowpeas highlight alternatives for crop management and conservation**

*Pablo G. Acosta-Quezada^1^, Edin H. Valladolid-Salinas^1^, Janina M. Murquincho-Chuncho^1^, Eudaldo Jadán-Veriñas^2^ & Mario X. Ruiz-González^1,3,*^.*

^1^ Universidad Técnica Particular de Loja - UTPL, Departamento de CC. Biológicas y Agropecuarias, San Cayetano Alto, Calle Marcelino Champagnat s/n. Apartado Postal 11-01-608, Loja, Ecuador.

^2^ Universidad Técnica de Machala – UTMACH, Facultad de CC. Agropecuarias, Machala, El Oro, Ecuador.

^3^ Current address: Instituto Universitario de Conservación y Mejora de la Agrodiversidad Valenciana, Universitat Politècnica de València, Camino de Vera s/n, Valencia 46022, Spain

**Supporting Table S1. Phytogenetic material**.

| **UTPL Bank ID** | **Code** | **Species** | **Variety** | **Province** | **Canton** | **Altitude (m)** |
| --- | --- | --- | --- | --- | --- | --- |
| UTPL-PGR-0156 | 1 | *P. vulgaris* | Bola amarillo | Loja | Saraguro | 2500 |
| UTPL-PGR-0168 | 2 | *P. vulgaris* | Bola rojo kusi | Loja | Saraguro | 2500 |
| UTPL-PGR-0311 | 3 | *P. vulgaris* | Bola trepador | Loja | Paltas | 1825 |
| UTPL-PGR-0314 | 4 | *P. vulgaris* | Bola negro | Loja | Paltas | 1086 |
| UTPL-PGR-0316 | 5 | *P. vulgaris* | Chacra | Loja | Paltas | 1326 |
| UTPL-PGR-0318 | 6 | *P. vulgaris* | - | Loja | Paltas | 1800 |
| UTPL-PGR-0344 | 7 | *P. vulgaris* | Panamito | Loja | Paltas | 1849 |
| UTPL-PGR-0345 | 8 | *P. vulgaris* | Bola grande | Loja | Paltas | 2534 |
| Commercial | 9 | *P. vulgaris* | Canario | - | - |  |
| UTPL-PGR-0798 | 12 | *P. vulgaris* | - | El Oro | Zaruma | 2644 |
| UTPL-PGR-0313 | 10 | Vigna sp. | Boca negra | Loja | Paltas | 1108 |
| UTPL-PGR-0317 | 11 | Vigna sp. | - | Loja | Paltas | 903 |
| UTPL-PGR-0230 | 13 | *P. lunatus* | Torta | Loja | Saraguro | 2520 |

**Supporting Figure S1. Morphological characteristics of the landraces**. Spp: species; L: landrace; CB: climatic background of the landrace (snowflake, cold; thermometer, warm; market trolley, commercial). Landrace 8 lacks some features because did not express those characters.


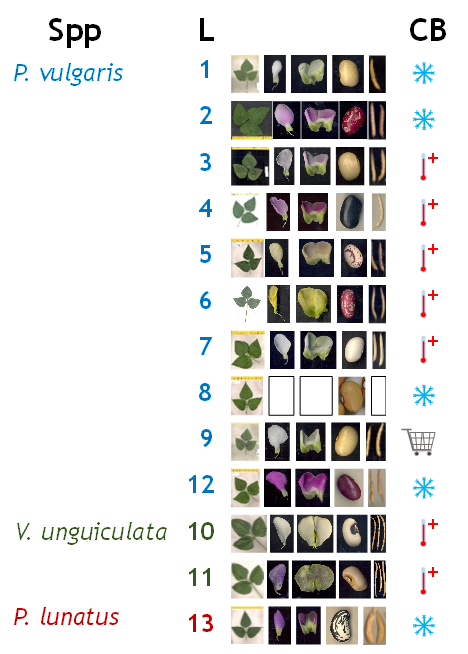


**Supporting Table S2. Agronomic quantitative (Q#) and phenological (P#) traits.**

| **Evaluated trait** | **Code** | **Instructions for measurement and description** |
| --- | --- | --- |
| **I. Plant architecture** |  |  |
| Stem length (cm) | Q1 | Distance from the stem base to the apical shoot |
| Stem diameter (mm) | Q2 | Measured at the middle point of the stem |
| Number of main branches | Q3 | Count of main branches |
| Branch orientation (°) | Q4 | Mean of 5 measures per plant with a goniometer |
| Composite sheet length (mm) | Q6 | Mean of 5 measures per plant with ImageJ software |
| Composite sheet width (mm) | Q7 | Mean of 5 measures per plant with ImageJ software |
| Apex angle of terminal leaflet (°) | Q8 | Mean of 5 measures per plant with ImageJ software |
| Terminal leaflet length (mm) | Q9 | Mean of 5 measures per plant with ImageJ software |
| Terminal leaflet width (mm) | Q10 | Mean of 5 measures per plant with ImageJ software |
| Angle of the base of the terminal leaflet (°) | Q11 | Mean of 5 measures per plant with ImageJ software |
| **II. Flower and fruit characteristics and yield** | | |
| Number of flowers per plant | Q12 | Hand counting |
| Peduncle length (mm) | Q15 | Mean of 5 measures per plant with ImageJ software |
| Left wing length (mm) | Q16 | Mean of 5 measures per plant with ImageJ software |
| Right wing length (mm) | Q17 | Mean of 5 measures per plant with ImageJ software |
| Banner length (mm) | Q18 | Mean of 5 measures per plant with ImageJ software |
| Style length (mm) | Q19 | Mean of 5 measures per plant with ImageJ software |
| Chalice length (mm) | Q20 | Mean of 5 measures per plant with ImageJ software |
| Number of pods per plant | Q21 | Hand counting |
| Number of pods per infructescence | Q22 | Mean of 5 measures per plant with ImageJ software |
| Sheath length (mm) | Q23 | Mean of 5 measures per plant with ImageJ software |
| Sheath width (mm) | Q24 | Mean of 5 measures per plant with ImageJ software |
| Sheath thickness (mm) | Q25 | Mean of 5 measures per plant with ImageJ software |
| Number of loculi per pod | Q26 | Mean of 5 measures per plant with ImageJ software |
| Number of grains per pod | Q27 | Mean of 5 measures per plant with ImageJ software |
| Grain length (mm) | Q28 | Mean of 20 seeds measured per plant with ImageJ software |
| Grain width (mm) | Q29 | Mean of 20 seeds measured per plant per plant with ImageJ software |
| Scar length (mm) | Q30 | Mean of 20 seeds measured per plant with a calliper |
| Grain thickness (mm) | Q31 | Mean of 20 seeds measured per plant |
| 100 seed weight (g) | Q32 | Weight of 100 seeds |
| Gross weight of seeds / plant (g) | Q33 | Total gross weight of seeds produced |
| Net weight of seed / plant (g) | Q34 | Total net weight of seeds produced |
| Number of seeds | Q35 | Total number of seeds produced |
| **III. Phenology** |  |  |
| Hypocotyl reaches the soil surface | P08 | Day when the hypocotyl arch is visible. |
| Emergence: hypocotyl with cotyledons break through soil surface (“cracking stage”) | P09 | Day when the cotyledons break through soil surface |
| Cotyledons completely unfolded | P10 | Day when the cotyledons unfold |
| 2 full leaves (first leaf pair unfolded) | P12 | Day when the first pair of leaves unfolds |
| 3rd true leaf (first trifoliate leaf) unfolded | P13 | Day when the first trifoliate leaf unfolds |
| 9 or more leaves (2 full leaves, 7 or more trifoliate) unfolded | P19 | Day when nine leaves are unfolded |
| First side shoot visible | P21 | Day when the first side shoot visible |
| First flower buds visible | P51 | Day when the first flower bud is visible |
| First flower buds enlarged | P55 | Day when the first flower buds are enlarged |
| First petals visible, flowers still closed | P59 | Day when first petals are visible |
| Beginning of flowering: 10% of flowers open. Beginning of flowering | P61 | Day when the 10% of flowers are opened |
| Full flowering: 50% of flowers open. Main flowering period | P65 | Day when the 50% of flowers are opened |
| Flowering finishing: majority of petals fallen or dry | P67 | Day when the 100% of flowers are opened |
| End of flowering: first pods visible | P69 | Day when the first pod is visible |
| 10% of pods ripe (beans hard). Seeds beginning to mature | P81 | Day when the 10% of pods are visible |
| 50% of pods ripe (beans hard). Main period of ripening | P85 | Day when the 50% of pods are visible |
| Fully ripe: pods ripe (beans hard) | P89 | Day when the 100% of pods are visible |

**Supporting Table S3. Statistical characterization of plant architecture traits.** Landraces 1-8 and 12 are *P. vulgaris* landraces; 9 is the commercial *P. vulgaris* cultivar; 10 and 11 the *V. unguiculata* landraces; and 13 the *P. lunatus* landrace. M.R.: Mean Rank after Kruskal-Wallis test; I.R.: interquartile range.

| **Treatment** | **Landrace** | **Statistic** | **Q1** | **Q2** | **Q4** | **Q6** | **Q7** | **Q8** | **Q9** | **Q10** | **Q11** |
| --- | --- | --- | --- | --- | --- | --- | --- | --- | --- | --- | --- |
| **Field** | **1** | **N** | 4 | 4 | 4 |  |  |  |  |  |  |
|  |  | **Mean** | 8.55 | 11.68 | 137.92 |  |  |  |  |  |  |
|  |  | **Median** | 8.50 | 11.67 | 138.34 |  |  |  |  |  |  |
|  |  | **M.R.** | 320.38 | 298.50 | 240.63 |  |  |  |  |  |  |
|  |  | **Variance** | 9.21 | 2.05 | 68.75 |  |  |  |  |  |  |
|  |  | **Minimum** | 5.20 | 10.15 | 127.50 |  |  |  |  |  |  |
|  |  | **Maximum** | 12.00 | 13.24 | 147.50 |  |  |  |  |  |  |
|  |  | **I.R.** | 5.85 | 2.73 | 15.83 |  |  |  |  |  |  |
|  | **2** | **N** | 20 | 20 | 20 | 19 | 19 | 19 | 19 | 19 | 19 |
|  |  | **Mean** | 6.40 | 7.46 | 122.88 | 196.70 | 168.23 | 58.16 | 96.87 | 75.04 | 149.27 |
|  |  | **Median** | 6.50 | 7.34 | 123.33 | 205.19 | 156.87 | 57.57 | 94.74 | 74.18 | 149.00 |
|  |  | **M.R.** | 236.33 | 134.65 | 133.00 | 115.18 | 145.16 | 151.79 | 138.00 | 149.47 | 284.76 |
|  |  | **Variance** | 3.83 | 5.42 | 226.47 | 718.97 | 409.70 | 122.31 | 91.18 | 92.51 | 36.03 |
|  |  | **Minimum** | 3.00 | 4.00 | 95.00 | 144.66 | 144.70 | 37.01 | 78.22 | 58.09 | 139.74 |
|  |  | **Maximum** | 11.00 | 12.81 | 160.00 | 254.34 | 211.12 | 88.61 | 114.42 | 97.17 | 159.85 |
|  |  | **I.R.** | 2.75 | 3.14 | 23.83 | 28.14 | 36.01 | 12.84 | 14.94 | 13.89 | 11.36 |
|  | **3** | **N** | 23 | 23 | 23 | 24 | 24 | 24 | 24 | 24 | 24 |
|  |  | **Mean** | 6.74 | 9.02 | 130.37 | 219.96 | 166.80 | 60.84 | 99.10 | 84.56 | 140.22 |
|  |  | **M.R.** | 254.28 | 200.80 | 181.37 | 184.52 | 131.85 | 171.60 | 161.48 | 247.02 | 217.75 |
|  |  | **Median** | 7.00 | 8.73 | 130.00 | 210.69 | 162.88 | 56.60 | 93.14 | 87.08 | 139.50 |
|  |  | **Variance** | 3.32 | 5.72 | 129.66 | 773.07 | 161.20 | 162.19 | 293.03 | 103.60 | 128.89 |
|  |  | **Minimum** | 4.00 | 4.13 | 106.67 | 186.80 | 142.36 | 44.80 | 75.50 | 69.14 | 116.59 |
|  |  | **Maximum** | 10.00 | 13.72 | 155.00 | 291.65 | 193.60 | 92.76 | 131.09 | 100.11 | 159.79 |
|  |  | **I.R.** | 3.00 | 2.60 | 16.00 | 40.11 | 18.73 | 20.53 | 30.40 | 19.38 | 18.28 |
|  | **4** | **N** | 17 | 17 | 17 | 3 | 3 | 3 | 3 | 3 | 3 |
|  |  | **Mean** | 5.82 | 6.69 | 130.62 | 138.93 | 127.30 | 75.33 | 87.11 | 79.89 | 127.05 |
|  |  | **Median** | 6.00 | 6.43 | 130.00 | 143.35 | 132.48 | 72.41 | 83.42 | 79.72 | 123.93 |
|  |  | **M.R.** | 200.76 | 98.85 | 181.24 | 31.67 | 32.00 | 253.50 | 74.33 | 201.17 | 127.67 |
|  |  | **Variance** | 1.15 | 1.24 | 107.56 | 322.15 | 80.71 | 654.61 | 119.64 | 38.34 | 40.41 |
|  |  | **Minimum** | 4.00 | 5.04 | 115.00 | 119.18 | 116.93 | 51.33 | 78.50 | 73.79 | 122.85 |
|  |  | **Maximum** | 7.00 | 8.61 | 150.00 | 154.25 | 132.50 | 102.25 | 99.42 | 86.17 | 134.36 |
|  |  | **I.R.** | 2.00 | 1.99 | 16.25 | . | . | . | . | . | . |
|  | **5** | **N** | 20 | 20 | 20 | 7 | 7 | 7 | 7 | 7 | 7 |
|  |  | **Mean** | 5.65 | 7.06 | 139.48 | 210.42 | 173.72 | 62.91 | 100.60 | 76.52 | 133.35 |
|  |  | **Median** | 6.00 | 7.27 | 140.00 | 210.50 | 177.83 | 62.71 | 102.80 | 73.23 | 138.86 |
|  |  | **M.R.** | 185.85 | 119.10 | 256.83 | 157.57 | 170.00 | 206.64 | 165.00 | 160.14 | 161.43 |
|  |  | **Variance** | 1.19 | 1.95 | 33.72 | 617.22 | 190.97 | 25.93 | 36.70 | 53.66 | 77.13 |
|  |  | **Minimum** | 4.00 | 4.35 | 130.00 | 184.09 | 156.25 | 58.06 | 90.34 | 69.30 | 118.73 |
|  |  | **Maximum** | 8.00 | 9.61 | 150.00 | 247.71 | 195.65 | 72.26 | 106.11 | 87.73 | 141.85 |
|  |  | **I.R.** | 1.00 | 2.08 | 7.25 | 48.74 | 22.15 | 7.61 | 9.01 | 12.99 | 13.67 |
|  | **6** | **N** | 4 | 4 | 4 |  |  |  |  |  |  |
|  |  | **Mean** | 4.88 | 6.48 | 142.25 |  |  |  |  |  |  |
|  |  | **Median** | 4.50 | 6.45 | 142.50 |  |  |  |  |  |  |
|  |  | **M.R.** | 130.13 | 88.75 | 288.63 |  |  |  |  |  |  |
|  |  | **Variance** | 2.40 | 0.91 | 2.75 |  |  |  |  |  |  |
|  |  | **Minimum** | 3.50 | 5.34 | 140.00 |  |  |  |  |  |  |
|  |  | **Maximum** | 7.00 | 7.68 | 144.00 |  |  |  |  |  |  |
|  |  | **I.R.** | 2.88 | 1.77 | 3.00 |  |  |  |  |  |  |
|  | **7** | **N** | 7 | 7 | 7 | 7 | 7 | 7 | 7 | 7 | 7 |
|  |  | **Mean** | 4.79 | 6.72 | 133.14 | 224.28 | 210.00 | 70.73 | 108.70 | 88.25 | 156.35 |
|  |  | **Median** | 5.50 | 7.15 | 135.00 | 237.32 | 210.30 | 68.31 | 109.23 | 88.68 | 158.38 |
|  |  | **M.R.** | 134.50 | 102.07 | 212.00 | 216.86 | 322.14 | 269.29 | 238.79 | 281.79 | 353.43 |
|  |  | **Variance** | 2.08 | 1.36 | 193.89 | 805.06 | 698.64 | 72.20 | 288.13 | 261.77 | 29.93 |
|  |  | **Minimum** | 2.00 | 4.96 | 110.00 | 186.64 | 172.69 | 59.32 | 83.68 | 58.21 | 145.58 |
|  |  | **Maximum** | 6.00 | 8.04 | 148.00 | 262.73 | 246.07 | 83.48 | 131.40 | 103.51 | 161.17 |
|  |  | **I.R.** | 2.00 | 2.29 | 22.50 | 49.77 | 45.51 | 14.22 | 31.16 | 23.95 | 7.13 |
|  | **8** | **N** |  |  |  |  |  |  |  |  |  |
|  |  | **Mean** |  |  |  |  |  |  |  |  |  |
|  |  | **Median** |  |  |  |  |  |  |  |  |  |
|  |  | **M.R.** |  |  |  |  |  |  |  |  |  |
|  |  | **Variance** |  |  |  |  |  |  |  |  |  |
|  |  | **Minimum** |  |  |  |  |  |  |  |  |  |
|  |  | **Maximum** |  |  |  |  |  |  |  |  |  |
|  |  | **I.R.** |  |  |  |  |  |  |  |  |  |
|  | **9** | **N** | 18 | 18 | 18 | 10 | 10 | 10 | 10 | 10 | 10 |
|  |  | **Mean** | 6.97 | 8.46 | 136.70 | 235.65 | 183.74 | 63.44 | 103.45 | 91.82 | 150.49 |
|  |  | **Median** | 6.74 | 7.85 | 136.25 | 234.57 | 179.43 | 65.89 | 103.55 | 93.44 | 150.01 |
|  |  | **M.R.** | 262.69 | 173.97 | 226.53 | 259.95 | 225.30 | 208.90 | 193.10 | 328.5 | 297.65 |
|  |  | **Variance** | 3.19 | 4.06 | 311.96 | 176.56 | 149.48 | 114.14 | 20.09 | 36.29 | 24.92 |
|  |  | **Minimum** | 5.00 | 6.01 | 107.50 | 215.21 | 170.60 | 43.39 | 96.08 | 80.69 | 142.67 |
|  |  | **Maximum** | 11.00 | 12.73 | 175.00 | 262.48 | 210.20 | 74.61 | 111.79 | 100.79 | 157.33 |
|  |  | **I.R.** | 2.50 | 3.20 | 27.58 | 18.45 | 18.35 | 15.52 | 6.48 | 8.81 | 8.37 |
|  | **12** | **N** | 8 | 8 | 8 | 8 | 8 | 8 | 8 | 8 | 8 |
|  |  | **Mean** | 5.00 | 8.11 | 126.35 | 213.04 | 175.19 | 58.42 | 103.59 | 86.68 | 148.35 |
|  |  | **Median** | 5.00 | 8.34 | 123.25 | 214.18 | 179.46 | 58.46 | 104.45 | 85.64 | 149.07 |
|  |  | **M.R.** | 136.31 | 171.19 | 151.5 | 151.88 | 178.88 | 157.75 | 199.25 | 268.69 | 277.19 |
|  |  | **Variance** | 1.14 | 1.63 | 136.68 | 52.97 | 92.42 | 47.67 | 103.79 | 85.83 | 56.00 |
|  |  | **Minimum** | 4.00 | 6.10 | 113.33 | 199.36 | 161.79 | 48.26 | 92.32 | 74.79 | 137.69 |
|  |  | **Maximum** | 7.00 | 9.64 | 148.00 | 221.54 | 186.83 | 66.68 | 118.77 | 102.93 | 159.68 |
|  |  | **I.R.** | 1.75 | 2.38 | 17.75 | 10.83 | 16.94 | 12.86 | 19.65 | 14.89 | 13.54 |
|  | **10** | **N** | 16 | 16 |  | 8 | 8 | 8 | 8 | 8 | 8 |
|  |  | **Mean** | 5.13 | 10.03 |  | 229.38 | 194.90 | 95.43 | 120.31 | 77.60 | 90.82 |
|  |  | **Median** | 5.00 | 9.59 |  | 232.04 | 190.65 | 91.08 | 120.24 | 79.69 | 91.95 |
|  |  | **M.R.** | 153.94 | 241.97 |  | 234.38 | 245.88 | 366.88 | 337.88 | 178.63 | 37.44 |
|  |  | **Variance** | 2.38 | 4.07 |  | 479.31 | 1379.99 | 270.85 | 70.43 | 58.27 | 42.94 |
|  |  | **Minimum** | 2.00 | 7.30 |  | 191.59 | 147.57 | 72.98 | 104.45 | 62.47 | 79.24 |
|  |  | **Maximum** | 8.00 | 14.90 |  | 257.85 | 249.47 | 127.16 | 130.62 | 85.22 | 98.65 |
|  |  | **I.R.** | 2.00 | 3.18 |  | 35.35 | 68.69 | 19.79 | 11.74 | 11.44 | 10.84 |
|  | **11** | **N** | 8 | 8 |  | 16 | 16 | 16 | 16 | 16 | 16 |
|  |  | **Mean** | 5.00 | 14.27 |  | 240.89 | 180.52 | 70.37 | 116.16 | 70.51 | 90.87 |
|  |  | **Median** | 5.00 | 13.41 |  | 251.51 | 176.91 | 70.49 | 115.52 | 68.79 | 89.62 |
|  |  | **M.R.** | 134.75 | 349.38 |  | 279.06 | 200.75 | 259.75 | 286.69 | 98.91 | 35.31 |
|  |  | **Variance** | 0.57 | 4.96 |  | 584.04 | 617.40 | 125.86 | 237.58 | 90.34 | 39.07 |
|  |  | **Minimum** | 4.00 | 11.92 |  | 199.72 | 149.78 | 50.27 | 92.85 | 59.02 | 81.86 |
|  |  | **Maximum** | 6.00 | 18.25 |  | 273.45 | 221.03 | 92.83 | 144.39 | 99.42 | 100.98 |
|  |  | **I.R.** | 1.50 | 3.53 |  | 37.86 | 46.77 | 14.50 | 27.88 | 6.55 | 11.54 |
|  | **13** | **N** | 15 | 15 | 15 | 24 | 24 | 24 | 24 | 24 | 24 |
|  |  | **Mean** | 7.33 | 17.51 | 111.14 | 210.24 | 170.20 | 63.57 | 93.39 | 73.85 | 149.90 |
|  |  | **Median** | 7.00 | 17.45 | 106.00 | 208.34 | 169.27 | 62.36 | 93.76 | 73.99 | 149.99 |
|  |  | **M.R.** | 301.00 | 372.77 | 87.87 | 143.46 | 146.35 | 200.79 | 100.77 | 129.21 | 290.50 |
|  |  | **Variance** | 1.42 | 22.61 | 395.60 | 193.88 | 101.06 | 155.43 | 23.29 | 30.65 | 30.74 |
|  |  | **Minimum** | 5.50 | 7.04 | 82.00 | 181.35 | 147.38 | 41.59 | 83.79 | 64.70 | 138.57 |
|  |  | **Maximum** | 9.00 | 24.85 | 155.00 | 235.56 | 193.25 | 95.20 | 101.04 | 85.58 | 159.93 |
|  |  | **I.R.** | 2.50 | 5.07 | 17.50 | 18.77 | 9.14 | 16.17 | 7.91 | 7.98 | 5.88 |
| **Greenhouse** | **1** | **N** | 24 | 24 | 24 | 24 | 24 | 24 | 24 | 24 | 24 |
|  |  | **Mean** | 9.56 | 12.56 | 139.93 | 247.20 | 183.99 | 54.34 | 103.10 | 92.51 | 153.45 |
|  |  | **Median** | 10.00 | 12.96 | 139.00 | 246.79 | 182.79 | 56.02 | 102.56 | 91.36 | 153.80 |
|  |  | **M.R.** | 345.46 | 311.35 | 250.50 | 311.38 | 230.54 | 115.98 | 193.00 | 327.15 | 324.77 |
|  |  | **Variance** | 9.64 | 5.42 | 178.99 | 201.70 | 219.83 | 32.76 | 82.98 | 56.88 | 28.20 |
|  |  | **Minimum** | 4.00 | 7.73 | 115.00 | 227.93 | 146.57 | 41.89 | 88.22 | 77.15 | 143.39 |
|  |  | **Maximum** | 14.00 | 16.71 | 166.67 | 278.67 | 210.18 | 62.73 | 120.30 | 109.08 | 163.15 |
|  |  | **I.R.** | 5.75 | 4.30 | 23.12 | 20.60 | 20.85 | 8.13 | 13.05 | 8.21 | 7.92 |
|  | **2** | **N** | 22 | 22 | 22 | 24 | 24 | 24 | 24 | 24 | 24 |
|  |  | **Mean** | 7.25 | 7.51 | 139.20 | 218.46 | 193.03 | 55.47 | 110.85 | 83.90 | 141.47 |
|  |  | **Median** | 7.25 | 6.73 | 140.00 | 217.41 | 194.28 | 53.29 | 112.24 | 86.02 | 144.12 |
|  |  | **M.R.** | 248.48 | 131.05 | 238.07 | 195.92 | 270.56 | 126.63 | 262.31 | 249.75 | 224.90 |
|  |  | **Variance** | 11.85 | 7.00 | 381.16 | 1471.82 | 398.85 | 93.93 | 116.51 | 119.64 | 110.41 |
|  |  | **Minimum** | 2.00 | 4.00 | 110.00 | 146.03 | 155.48 | 40.64 | 92.24 | 58.70 | 124.99 |
|  |  | **Maximum** | 15.00 | 13.97 | 170.00 | 282.67 | 238.11 | 84.36 | 139.30 | 100.63 | 157.99 |
|  |  | **I.R.** | 6.00 | 4.10 | 36.25 | 51.16 | 27.21 | 12.12 | 15.12 | 11.85 | 17.64 |
|  | **3** | **N** | 24 | 24 | 24 | 24 | 24 | 24 | 24 | 24 | 24 |
|  |  | **Mean** | 7.15 | 9.50 | 134.40 | 198.83 | 166.82 | 66.37 | 94.22 | 77.49 | 139.39 |
|  |  | **Median** | 7.25 | 9.10 | 136.34 | 184.11 | 170.02 | 68.15 | 90.95 | 75.81 | 143.73 |
|  |  | **M.R.** | 286.40 | 214.75 | 212.00 | 142.58 | 134.54 | 221.69 | 128.00 | 170.50 | 224.15 |
|  |  | **Variance** | 2.27 | 8.76 | 105.50 | 1765.57 | 208.58 | 175.54 | 206.48 | 40.94 | 298.70 |
|  |  | **Minimum** | 4.00 | 4.00 | 120.00 | 128.85 | 142.04 | 44.83 | 73.15 | 67.11 | 111.54 |
|  |  | **Maximum** | 10.00 | 14.71 | 156.67 | 288.52 | 196.66 | 92.01 | 127.00 | 90.98 | 167.50 |
|  |  | **I.R.** | 2.00 | 3.97 | 18.88 | 59.50 | 23.77 | 22.98 | 20.55 | 10.05 | 33.71 |
|  | **4** | **N** | 21 | 21 | 21 | 13 | 13 | 13 | 13 | 13 | 13 |
|  |  | **Mean** | 4.7619 | 6.5276 | 144.7381 | 156.2462 | 143.1408 | 77.1931 | 92.7362 | 75.3362 | 126.8754 |
|  |  | **Median** | 4 | 6.15 | 146 | 139.72 | 121.84 | 77.31 | 91.07 | 75.19 | 126.25 |
|  |  | **M.R.** | 119.38 | 90.69 | 294.14 | 78.00 | 117.35 | 307.96 | 102.88 | 145.54 | 138.31 |
|  |  | **Variance** | 1.215 | 3.728 | 231.959 | 2787.366 | 1886.049 | 107.01 | 61.609 | 33.525 | 140.046 |
|  |  | **Minimum** | 3.5 | 4.01 | 96 | 93.22 | 95.83 | 59.88 | 82.01 | 65.03 | 109.17 |
|  |  | **Maximum** | 7 | 12.03 | 166 | 223.18 | 219.44 | 93.28 | 105.95 | 85.15 | 150.13 |
|  |  | **I.R.** | 1 | 2.35 | 16.42 | 106.62 | 86.66 | 14.24 | 14.38 | 8.74 | 11.76 |
|  | **5** | **N** | 16 | 16 | 16 | 24 | 24 | 24 | 24 | 24 | 24 |
|  |  | **Mean** | 5.69 | 6.93 | 140.25 | 231.31 | 182.53 | 57.30 | 105.49 | 81.51 | 134.97 |
|  |  | **Median** | 6.00 | 7.16 | 140.00 | 230.30 | 182.49 | 56.33 | 105.07 | 78.09 | 138.27 |
|  |  | **M.R.** | 188.69 | 115.16 | 262.19 | 234.94 | 218.15 | 144.96 | 216.92 | 212.71 | 179.67 |
|  |  | **Variance** | 1.03 | 3.22 | 36.03 | 690.86 | 252.15 | 33.02 | 60.81 | 104.18 | 144.89 |
|  |  | **Minimum** | 4.00 | 3.80 | 128.00 | 186.83 | 159.02 | 50.11 | 87.56 | 66.14 | 114.90 |
|  |  | **Maximum** | 8.00 | 10.30 | 154.00 | 286.76 | 218.49 | 71.28 | 120.10 | 103.50 | 152.65 |
|  |  | **I.R.** | 1.00 | 2.71 | 6.00 | 50.26 | 26.66 | 8.89 | 11.75 | 18.06 | 22.84 |
|  | **6** | **N** | 13 | 13 | 13 | 16 | 16 | 16 | 16 | 16 | 16 |
|  |  | **Mean** | 4.27 | 6.65 | 150.19 | 110.39 | 88.58 | 77.35 | 57.65 | 42.98 | 121.98 |
|  |  | **Median** | 4.00 | 6.05 | 148.00 | 105.56 | 88.14 | 75.35 | 56.08 | 42.52 | 121.46 |
|  |  | **M.R.** | 120.27 | 102.15 | 324.92 | 14.13 | 9.75 | 300.94 | 9.38 | 11.56 | 114.63 |
|  |  | **Variance** | 5.94 | 4.10 | 145.98 | 443.25 | 175.74 | 165.72 | 63.84 | 11.11 | 125.59 |
|  |  | **Minimum** | 1.00 | 4.21 | 128.00 | 78.08 | 59.84 | 52.88 | 46.74 | 37.01 | 97.95 |
|  |  | **Maximum** | 9.00 | 10.21 | 170.00 | 153.19 | 111.03 | 96.94 | 73.01 | 50.13 | 142.59 |
|  |  | **I.R.** | 4.00 | 3.17 | 19.00 | 32.61 | 19.10 | 21.69 | 14.11 | 3.36 | 16.19 |
|  | **7** | **N** | 16 | 16 | 16 | 16 | 16 | 16 | 16 | 16 | 16 |
|  |  | **Mean** | 5.34 | 7.70 | 140.22 | 175.02 | 158.71 | 69.32 | 89.34 | 66.26 | 134.11 |
|  |  | **Median** | 5.75 | 7.68 | 140.00 | 157.08 | 126.05 | 69.92 | 78.56 | 54.44 | 131.62 |
|  |  | **M.R.** | 169.09 | 146.75 | 257.44 | 111.28 | 167.69 | 251.09 | 127.16 | 144.44 | 168.72 |
|  |  | **Variance** | 2.19 | 2.14 | 90.80 | 2637.92 | 2888.67 | 125.46 | 572.00 | 631.43 | 58.99 |
|  |  | **Minimum** | 2.00 | 4.90 | 128.00 | 115.37 | 104.04 | 52.11 | 61.81 | 39.78 | 121.15 |
|  |  | **Maximum** | 8.00 | 10.64 | 162.50 | 266.58 | 247.52 | 89.45 | 139.45 | 109.25 | 148.58 |
|  |  | **I.R.** | 1.88 | 1.32 | 12.25 | 94.38 | 96.43 | 16.44 | 39.18 | 42.25 | 13.52 |
|  | **8** | **N** | 15 | 15 | 15 | 16 | 16 | 16 | 16 | 16 | 16 |
|  |  | **Mean** | 6.42 | 11.80 | 142.67 | 232.06 | 187.06 | 45.76 | 113.10 | 87.81 | 137.08 |
|  |  | **Median** | 6.00 | 12.15 | 143.33 | 237.23 | 182.86 | 45.01 | 110.62 | 87.29 | 134.22 |
|  |  | **M.R.** | 227.67 | 276.13 | 267.87 | 246.94 | 240.56 | 41.91 | 282.19 | 283.81 | 193.13 |
|  |  | **Variance** | 4.28 | 12.98 | 409.57 | 479.15 | 295.12 | 39.77 | 80.63 | 62.07 | 97.54 |
|  |  | **Minimum** | 3.50 | 6.24 | 100.00 | 190.12 | 160.98 | 36.38 | 104.13 | 75.75 | 123.96 |
|  |  | **Maximum** | 11.00 | 18.00 | 180.00 | 262.50 | 226.30 | 63.02 | 132.88 | 100.09 | 150.33 |
|  |  | **I.R.** | 3.00 | 7.11 | 29.17 | 34.28 | 13.49 | 6.96 | 5.46 | 12.85 | 19.40 |
|  | **9** | **N** | 24 | 24 | 24 | 24 | 24 | 24 | 24 | 24 | 24 |
|  |  | **Mean** | 7.44 | 9.31 | 139.94 | 234.24 | 179.24 | 60.14 | 102.98 | 86.98 | 136.25 |
|  |  | **Median** | 7.25 | 9.10 | 142.50 | 236.11 | 178.74 | 59.85 | 104.28 | 86.82 | 132.53 |
|  |  | **M.R.** | 292.42 | 209.77 | 251.69 | 254.50 | 201.60 | 172.96 | 191.54 | 276.56 | 193.46 |
|  |  | **Variance** | 3.14 | 5.97 | 312.63 | 351.53 | 178.30 | 76.87 | 52.49 | 56.90 | 169.04 |
|  |  | **Minimum** | 5.00 | 4.35 | 105.00 | 196.57 | 154.29 | 44.40 | 87.80 | 73.15 | 118.49 |
|  |  | **Maximum** | 12.00 | 14.72 | 166.67 | 267.14 | 210.14 | 79.71 | 117.82 | 104.77 | 161.42 |
|  |  | **I.R.** | 2.75 | 3.53 | 29.46 | 21.72 | 17.19 | 14.20 | 10.17 | 10.65 | 20.73 |
|  | **12** | **N** | 24 | 24 | 24 | 24 | 24 | 24 | 24 | 24 | 24 |
|  |  | **Mean** | 5.52 | 8.51 | 133.90 | 224.09 | 186.74 | 61.50 | 107.00 | 88.44 | 152.74 |
|  |  | **Median** | 6.00 | 8.63 | 136.75 | 225.74 | 188.07 | 62.06 | 106.79 | 88.25 | 154.34 |
|  |  | **M.R.** | 178.56 | 181.23 | 207.96 | 209.33 | 245.25 | 188.33 | 231.75 | 289.31 | 316.08 |
|  |  | **Variance** | 2.13 | 4.06 | 123.50 | 317.82 | 206.84 | 76.83 | 54.04 | 70.65 | 65.13 |
|  |  | **Minimum** | 2.50 | 5.10 | 115.00 | 170.38 | 155.37 | 45.26 | 86.28 | 72.39 | 134.75 |
|  |  | **Maximum** | 9.02 | 11.72 | 160.00 | 252.15 | 213.99 | 76.31 | 123.41 | 106.07 | 167.87 |
|  |  | **I.R.** | 1.00 | 3.49 | 17.50 | 15.80 | 25.76 | 14.52 | 8.93 | 8.13 | 12.79 |
|  | **10** | **N** | 23 | 23 | 23 | 24 | 24 | 24 | 24 | 24 | 24 |
|  |  | **Mean** | 5.59 | 12.94 | 122.19 | 229.48 | 202.96 | 100.69 | 115.73 | 77.61 | 90.42 |
|  |  | **Median** | 5.00 | 13.31 | 128.00 | 232.68 | 196.70 | 101.98 | 115.47 | 80.17 | 88.76 |
|  |  | **M.R.** | 186.28 | 319.02 | 138.72 | 233.13 | 305.96 | 385.75 | 300.56 | 178.46 | 33.63 |
|  |  | **Variance** | 2.54 | 6.18 | 315.03 | 338.62 | 486.07 | 57.34 | 105.81 | 61.84 | 44.73 |
|  |  | **Minimum** | 1.00 | 8.08 | 90.00 | 193.81 | 162.28 | 81.22 | 97.18 | 56.65 | 78.44 |
|  |  | **Maximum** | 8.00 | 18.25 | 160.00 | 264.49 | 239.76 | 115.67 | 135.94 | 87.34 | 101.52 |
|  |  | **I.R.** | 2.00 | 3.03 | 28.00 | 22.81 | 40.16 | 8.76 | 16.93 | 9.98 | 9.26 |
|  | **11** | **N** | 24 | 24 | 24 | 24 | 24 | 24 | 24 | 24 | 24 |
|  |  | **Mean** | 3.96 | 15.77 | 111.35 | 239.79 | 177.79 | 71.97 | 114.97 | 70.29 | 92.79 |
|  |  | **Median** | 4.00 | 15.64 | 114.00 | 247.09 | 175.69 | 71.92 | 116.40 | 68.42 | 92.10 |
|  |  | **M.R.** | 97.31 | 366.58 | 85.75 | 278.81 | 189.33 | 274.29 | 286.54 | 96.02 | 40.52 |
|  |  | **Variance** | 3.17 | 10.69 | 326.53 | 361.03 | 437.58 | 104.80 | 165.43 | 61.68 | 57.57 |
|  |  | **Minimum** | 1.00 | 10.25 | 76.00 | 206.55 | 148.85 | 51.57 | 93.07 | 57.81 | 78.39 |
|  |  | **Maximum** | 6.00 | 22.36 | 146.00 | 266.01 | 219.32 | 94.57 | 143.39 | 97.52 | 109.75 |
|  |  | **I.R.** | 3.75 | 4.67 | 25.50 | 36.03 | 31.53 | 10.09 | 17.88 | 8.50 | 7.72 |
|  | **13** | **N** | 24 | 24 | 24 | 24 | 24 | 24 | 24 | 24 | 24 |
|  |  | **Mean** | 7.52 | 19.84 | 108.11 | 241.46 | 212.71 | 52.01 | 114.25 | 81.50 | 151.97 |
|  |  | **Median** | 8.00 | 18.45 | 105.34 | 238.28 | 209.95 | 52.02 | 115.26 | 81.51 | 154.77 |
|  |  | **M.R.** | 274.04 | 403.10 | 83.88 | 285.42 | 343.79 | 100.42 | 297.23 | 218.90 | 309.94 |
|  |  | **Variance** | 10.53 | 18.21 | 718.88 | 225.80 | 366.07 | 120.22 | 75.57 | 27.83 | 37.05 |
|  |  | **Minimum** | 2.00 | 13.62 | 70.00 | 220.97 | 174.67 | 36.58 | 93.71 | 70.47 | 141.98 |
|  |  | **Maximum** | 14.00 | 28.50 | 175.00 | 275.90 | 243.79 | 83.00 | 127.64 | 93.14 | 161.97 |
|  |  | **I.R.** | 5.00 | 6.50 | 25.50 | 22.04 | 33.22 | 14.96 | 11.36 | 7.43 | 11.10 |

**Supporting Table S4. Statistical characterization of plant reproductive and production traits.** Landraces 1-8 and 12 are *P. vulgaris* landraces; 9 is the commercial *P. vulgaris* cultivar; 10 and 11 the *V. unguiculata* landraces; and 13 the *P. lunatus* landrace. T: treatment; L: landrace; S: Statistic: F, open field; Gh: greenhouse. $\bar{x}$: mean; M: median; Var: variance; MR: Mean Rank after Kruskal-Wallis test; IR: interquartile range.

| **T** | **L** | **S** | **Q12** | **Q15** | **Q16** | **Q17** | **Q18** | **Q19** | **Q20** | **Q21** | **Q22** | **Q23** | **Q24** | **Q25** | **Q26** | **Q27** | **Q28** | **Q29** | **Q30** | **Q31** | **Q32** | **Q33** | **Q34** | **Q35** |
| --- | --- | --- | --- | --- | --- | --- | --- | --- | --- | --- | --- | --- | --- | --- | --- | --- | --- | --- | --- | --- | --- | --- | --- | --- |
| **F** | **1** | **N** | 7 | 4 | 4 | 4 | 4 | 4 | 4 | 8 | 8 | 4 | 4 | 4 | 4 | 8 | 4 | 4 | 4 | 4 | 8 | 8 | 8 | 8 |
|  |  | $\bar{\boldsymbol{x}}$ | 30.14 | 7.95 | 17.23 | 17.44 | 7.88 | 8.71 | 7.16 | 16.88 | 0.83 | 86.15 | 12.32 | 9.44 | 4.10 | 2.05 | 12.51 | 9.42 | 2.52 | 7.43 | 33.17 | 9.88 | 7.06 | 10.75 |
|  |  | **M** | 45.00 | 7.96 | 17.42 | 17.66 | 7.74 | 8.60 | 7.13 | 15.50 | 0.80 | 85.38 | 12.37 | 9.20 | 4.10 | 1.90 | 12.29 | 9.42 | 2.52 | 7.40 | 31.17 | 9.40 | 4.45 | 6.50 |
|  |  | **MR** | 199.93 | 175.25 | 184.75 | 190.25 | 189.63 | 148.13 | 204.13 | 212.81 | 152.81 | 10.50 | 221.75 | 199.38 | 109.88 | 212.56 | 206.50 | 239.38 | 133.13 | 256.00 | 261.25 | 209.00 | 201.06 | 188.44 |
|  |  | **Var** | 520.81 | 0.08 | 0.47 | 0.55 | 0.39 | 0.24 | 0.09 | 329.84 | 0.78 | 54.53 | 0.55 | 0.88 | 0.07 | 4.83 | 0.21 | 0.00 | 0.00 | 0.02 | 1261.16 | 112.09 | 64.58 | 154.50 |
|  |  | **Min** | 3.00 | 7.62 | 16.30 | 16.37 | 7.29 | 8.26 | 6.83 | 0.00 | 0.00 | 78.54 | 11.49 | 8.58 | 3.80 | 0.00 | 12.25 | 9.40 | 2.48 | 7.28 | 0.00 | 0.00 | 0.00 | 0.00 |
|  |  | **Max** | 52.00 | 8.28 | 17.78 | 18.05 | 8.77 | 9.38 | 7.55 | 37.00 | 1.80 | 95.28 | 13.03 | 10.77 | 4.40 | 4.40 | 13.19 | 9.43 | 2.57 | 7.62 | 68.79 | 20.77 | 18.63 | 30.00 |
|  |  | **IR** | 45.00 | 0.54 | 1.26 | 1.33 | 1.12 | 0.91 | 0.58 | 34.75 | 1.60 | 14.18 | 1.40 | 1.65 | 0.50 | 4.15 | 0.71 | 0.03 | 0.07 | 0.28 | 67.47 | 20.21 | 15.30 | 22.75 |
|  | **2** | **N** | 19 | 19 | 19 | 19 | 19 | 19 | 19 | 24 | 24 | 19 | 19 | 19 | 19 | 24 | 19 | 19 | 19 | 19 | 24 | 24 | 24 | 24 |
|  |  | $\bar{\boldsymbol{x}}$ | 24.53 | 8.40 | 16.12 | 16.57 | 6.80 | 10.30 | 7.36 | 11.25 | 1.26 | 124.01 | 13.87 | 10.43 | 5.20 | 4.07 | 12.59 | 9.58 | 2.42 | 7.79 | 53.98 | 12.04 | 8.33 | 12.21 |
|  |  | **M** | 22.00 | 8.36 | 16.89 | 16.61 | 6.84 | 10.14 | 7.02 | 7.50 | 1.50 | 128.06 | 13.53 | 10.32 | 5.00 | 4.80 | 12.65 | 9.57 | 2.43 | 7.76 | 66.73 | 11.43 | 7.88 | 11.50 |
|  |  | **MR** | 197.63 | 179.29 | 154.32 | 164.58 | 127.24 | 196.58 | 194.34 | 216.69 | 197.96 | 236.00 | 265.03 | 274.08 | 185.95 | 332.44 | 211.34 | 249.95 | 99.71 | 289.24 | 367.06 | 247.31 | 237.79 | 221.31 |
|  |  | **Var** | 133.15 | 1.26 | 2.69 | 2.91 | 0.12 | 1.69 | 0.88 | 106.20 | 0.48 | 109.31 | 1.42 | 0.25 | 0.63 | 5.00 | 0.39 | 0.07 | 0.03 | 0.06 | 807.80 | 99.07 | 48.05 | 104.26 |
|  |  | **Min** | 8.00 | 6.70 | 13.31 | 13.37 | 6.10 | 7.49 | 6.23 | 0.00 | 0.00 | 100.71 | 11.42 | 9.52 | 3.80 | 0.00 | 11.50 | 9.14 | 2.14 | 7.43 | 0.00 | 0.00 | 0.00 | 0.00 |
|  |  | **Max** | 59.00 | 10.68 | 17.92 | 21.13 | 7.42 | 12.04 | 8.96 | 37.00 | 2.00 | 137.25 | 16.03 | 11.32 | 6.60 | 6.60 | 13.59 | 10.09 | 2.71 | 8.32 | 74.28 | 36.27 | 24.89 | 38.00 |
|  |  | **IR** | 11.00 | 1.88 | 3.03 | 1.76 | 0.48 | 2.10 | 1.70 | 13.25 | 0.55 | 14.74 | 1.50 | 0.65 | 1.40 | 1.70 | 1.13 | 0.44 | 0.32 | 0.38 | 6.08 | 16.59 | 12.76 | 16.75 |
|  | **3** | **N** | 24 | 11 | 11 | 11 | 11 | 11 | 11 | 24 | 24 | 8 | 8 | 8 | 8 | 24 | 8 | 8 | 8 | 8 | 22 | 22 | 22 | 22 |
|  |  | $\bar{\boldsymbol{x}}$ | 18.13 | 6.53 | 17.05 | 17.22 | 6.69 | 8.65 | 6.13 | 9.83 | 0.89 | 105.21 | 12.90 | 9.61 | 4.13 | 1.33 | 11.97 | 9.47 | 2.43 | 7.43 | 25.17 | 9.91 | 5.55 | 8.05 |
|  |  | **M** | 8.00 | 6.82 | 16.68 | 17.42 | 6.81 | 8.64 | 6.04 | 1.00 | 1.00 | 105.02 | 12.88 | 9.42 | 4.20 | 0.00 | 11.98 | 9.42 | 2.42 | 7.42 | 0.00 | 0.00 | 0.00 | 0.00 |
|  |  | **MR** | 141.96 | 98.18 | 181.50 | 185.36 | 120.32 | 146.91 | 133.00 | 180.90 | 160.27 | 125.88 | 246.56 | 206.38 | 115.88 | 172.25 | 170.38 | 240.69 | 97.81 | 257.13 | 228.14 | 190.64 | 175.02 | 164.25 |
|  |  | **Var** | 354.64 | 0.49 | 1.63 | 1.71 | 0.19 | 0.29 | 0.14 | 185.97 | 0.67 | 15.12 | 0.33 | 0.59 | 0.25 | 3.70 | 0.36 | 0.22 | 0.01 | 0.03 | 1163.86 | 206.69 | 70.35 | 151.00 |
|  |  | **Min** | 1.00 | 4.86 | 15.22 | 14.88 | 5.89 | 7.50 | 5.50 | 0.00 | 0.00 | 100.29 | 12.09 | 8.94 | 3.40 | 0.00 | 11.27 | 8.98 | 2.31 | 7.19 | 0.00 | 0.00 | 0.00 | 0.00 |
|  |  | **Max** | 59.00 | 7.14 | 19.50 | 18.89 | 7.15 | 9.47 | 6.80 | 37.00 | 2.00 | 112.62 | 13.65 | 11.41 | 4.80 | 4.40 | 12.90 | 10.10 | 2.61 | 7.74 | 73.93 | 38.57 | 25.96 | 37.00 |
|  |  | **IR** | 33.25 | 0.81 | 1.87 | 1.52 | 0.47 | 0.62 | 0.39 | 25.75 | 1.73 | 5.43 | 1.13 | 0.53 | 0.90 | 3.75 | 1.02 | 0.86 | 0.15 | 0.20 | 68.02 | 27.71 | 11.53 | 16.75 |
|  | **4** | **N** | 17 | 9 | 9 | 9 | 9 | 9 | 9 | 18 | 18 | 9 | 9 | 9 | 9 | 18 | 9 | 9 | 9 | 9 | 18 | 18 | 18 | 18 |
|  |  | $\bar{\boldsymbol{x}}$ | 24.12 | 6.95 | 13.23 | 13.32 | 6.43 | 7.28 | 5.14 | 16.00 | 1.44 | 101.42 | 9.25 | 9.11 | 5.38 | 2.34 | 10.01 | 6.91 | 1.97 | 4.45 | 11.29 | 12.74 | 11.73 | 40.67 |
|  |  | **M** | 16.00 | 7.27 | 13.38 | 13.27 | 6.35 | 7.16 | 5.08 | 4.50 | 1.73 | 103.91 | 9.30 | 9.29 | 5.80 | 1.00 | 9.85 | 6.88 | 1.86 | 4.39 | 7.95 | 1.60 | 1.55 | 7.50 |
|  |  | **MR** | 181.68 | 120.33 | 75.67 | 74.61 | 99.22 | 69.44 | 40.17 | 234.42 | 241.64 | 90.44 | 71.61 | 183.56 | 190.61 | 233.64 | 71.56 | 86.00 | 37.78 | 8.83 | 178.00 | 214.08 | 216.47 | 230.89 |
|  |  | **Var** | 364.86 | 1.34 | 0.26 | 0.57 | 0.17 | 0.47 | 0.09 | 325.18 | 0.46 | 31.26 | 0.44 | 1.00 | 2.84 | 7.38 | 0.28 | 0.08 | 0.09 | 0.05 | 142.82 | 305.32 | 262.39 | 3655.18 |
|  |  | **Min** | 6.00 | 5.70 | 12.41 | 12.59 | 5.92 | 6.27 | 4.84 | 0.00 | 0.00 | 92.43 | 8.11 | 7.88 | 2.40 | 0.00 | 9.18 | 6.37 | 1.69 | 4.17 | 0.00 | 0.00 | 0.00 | 0.00 |
|  |  | **Max** | 63.00 | 8.62 | 13.92 | 14.90 | 7.05 | 8.41 | 5.74 | 58.00 | 2.00 | 107.43 | 10.08 | 10.52 | 7.20 | 6.60 | 10.78 | 7.44 | 2.67 | 4.79 | 30.68 | 52.68 | 50.05 | 189.00 |
|  |  | **IR** | 32.50 | 2.22 | 0.84 | 1.08 | 0.74 | 0.92 | 0.45 | 29.25 | 0.25 | 10.04 | 1.00 | 1.91 | 2.50 | 5.50 | 0.87 | 0.19 | 0.26 | 0.44 | 21.92 | 22.92 | 21.30 | 62.50 |
|  | **5** | **N** | 21 | 2 | 2 | 2 | 2 | 2 | 2 | 24 |  |  |  |  |  | 24 |  |  |  |  | 24 | 24 | 24 | 24 |
|  |  | $\bar{\boldsymbol{x}}$ | 8.24 | 6.14 | 13.12 | 13.39 | 6.63 | 7.23 | 6.17 | 4.25 |  |  |  |  |  |  |  |  |  |  |  |  |  |  |
|  |  | **M** | 8.00 | 6.14 | 13.12 | 13.39 | 6.63 | 7.23 | 6.17 | 4.00 |  |  |  |  |  |  |  |  |  |  |  |  |  |  |
|  |  | **MR** | 102.64 | 79.00 | 73.50 | 76.50 | 111.75 | 70.25 | 141.00 | 174.10 |  |  |  |  |  | 101.50 |  |  |  |  | 100.50 | 100.50 | 100.50 | 100.50 |
|  |  | **Var** | 24.69 | 2.25 | 0.00 | 0.11 | 0.09 | 0.41 | 0.02 | 12.20 |  |  |  |  |  |  |  |  |  |  |  |  |  |  |
|  |  | **Min** | 1.00 | 5.08 | 13.09 | 13.15 | 6.42 | 6.77 | 6.06 | 0.00 |  |  |  |  |  |  |  |  |  |  |  |  |  |  |
|  |  | **Max** | 21.00 | 7.20 | 13.15 | 13.62 | 6.84 | 7.68 | 6.28 | 10.00 |  |  |  |  |  |  |  |  |  |  |  |  |  |  |
|  |  | **IR** | 7.00 | . | . | . | . | . | . | 6.75 |  |  |  |  |  |  |  |  |  |  |  |  |  |  |
|  | **6** | **N** | 15 |  |  |  |  |  |  | 16 |  |  |  |  |  | 16 |  |  |  |  | 16 | 16 | 16 | 16 |
|  |  | $\bar{\boldsymbol{x}}$ | 7.60 |  |  |  |  |  |  | 2.00 |  |  |  |  |  |  |  |  |  |  |  |  |  |  |
|  |  | **M** | 7.00 |  |  |  |  |  |  | 2.00 |  |  |  |  |  |  |  |  |  |  |  |  |  |  |
|  |  | **MR** | 101.10 |  |  |  |  |  |  | 145.91 |  |  |  |  |  | 101.50 |  |  |  |  | 100.50 | 100.50 | 100.50 | 100.50 |
|  |  | **Var** | 10.11 |  |  |  |  |  |  | 3.87 |  |  |  |  |  |  |  |  |  |  |  |  |  |  |
|  |  | **Min** | 3.00 |  |  |  |  |  |  | 0.00 |  |  |  |  |  |  |  |  |  |  |  |  |  |  |
|  |  | **Max** | 14.00 |  |  |  |  |  |  | 7.00 |  |  |  |  |  |  |  |  |  |  |  |  |  |  |
|  |  | **IR** | 5.00 |  |  |  |  |  |  | 3.00 |  |  |  |  |  |  |  |  |  |  |  |  |  |  |
|  | **7** | **N** | 7 | 7 | 7 | 7 | 7 | 7 | 7 | 8 | 8 | 7 | 7 | 7 | 7 | 8 | 7 | 7 | 7 | 7 | 8 | 8 | 8 | 8 |
|  |  | $\bar{\boldsymbol{x}}$ | 15.43 | 6.99 | 14.14 | 15.12 | 8.08 | 8.62 | 6.20 | 7.88 | 1.40 | 106.25 | 11.30 | 7.49 | 5.40 | 4.65 | 9.95 | 6.99 | 2.46 | 5.58 | 28.44 | 8.88 | 7.61 | 23.50 |
|  |  | **M** | 13.00 | 6.42 | 14.75 | 16.21 | 8.38 | 8.72 | 6.57 | 8.00 | 1.50 | 105.91 | 11.15 | 7.44 | 5.60 | 5.40 | 9.93 | 6.89 | 2.47 | 5.59 | 32.13 | 10.02 | 8.48 | 26.50 |
|  |  | **MR** | 154.21 | 114.50 | 102.14 | 124.50 | 194.36 | 145.14 | 135.93 | 209.94 | 214.94 | 138.14 | 173.14 | 73.50 | 194.43 | 363.13 | 68.50 | 90.93 | 109.57 | 134.14 | 275.56 | 236.44 | 241.19 | 266.06 |
|  |  | **Var** | 47.62 | 2.44 | 3.79 | 3.65 | 0.48 | 0.18 | 0.49 | 23.55 | 0.35 | 21.19 | 0.28 | 0.24 | 0.93 | 4.27 | 0.03 | 0.08 | 0.01 | 0.02 | 132.82 | 28.00 | 23.69 | 226.00 |
|  |  | **Min** | 8.00 | 5.36 | 10.59 | 11.59 | 7.11 | 8.00 | 5.22 | 0.00 | 0.00 | 100.95 | 10.65 | 6.99 | 3.40 | 0.00 | 9.73 | 6.63 | 2.38 | 5.38 | 0.00 | 0.00 | 0.00 | 0.00 |
|  |  | **Max** | 27.00 | 8.92 | 16.56 | 16.71 | 8.81 | 9.12 | 6.89 | 16.00 | 1.80 | 114.62 | 12.09 | 8.46 | 6.40 | 6.40 | 10.21 | 7.29 | 2.54 | 5.81 | 33.84 | 15.21 | 14.12 | 42.00 |
|  |  | **IR** | 12.00 | 3.31 | 2.14 | 2.72 | 1.49 | 0.86 | 1.53 | 6.25 | 0.40 | 6.33 | 0.91 | 0.51 | 0.80 | 1.90 | 0.34 | 0.55 | 0.15 | 0.18 | 1.92 | 9.21 | 8.38 | 26.75 |
|  | **8** | **N** |  |  |  |  |  |  |  |  |  |  |  |  |  |  |  |  |  |  |  |  |  |  |
|  |  | $\bar{\boldsymbol{x}}$ |  |  |  |  |  |  |  |  |  |  |  |  |  |  |  |  |  |  |  |  |  |  |
|  |  | **M** |  |  |  |  |  |  |  |  |  |  |  |  |  |  |  |  |  |  |  |  |  |  |
|  |  | **MR** |  |  |  |  |  |  |  |  |  |  |  |  |  |  |  |  |  |  |  |  |  |  |
|  |  | **Var** |  |  |  |  |  |  |  |  |  |  |  |  |  |  |  |  |  |  |  |  |  |  |
|  |  | **Min** |  |  |  |  |  |  |  |  |  |  |  |  |  |  |  |  |  |  |  |  |  |  |
|  |  | **Max** |  |  |  |  |  |  |  |  |  |  |  |  |  |  |  |  |  |  |  |  |  |  |
|  |  | **IR** |  |  |  |  |  |  |  |  |  |  |  |  |  |  |  |  |  |  |  |  |  |  |
|  | **9** | **N** | 24 | 6 | 6 | 6 | 6 | 6 | 6 | 24 | 7 | 6 | 6 | 6 | 6 | 24 | 6 | 6 | 6 | 6 | 24 | 24 | 24 | 24 |
|  |  | $\bar{\boldsymbol{x}}$ | 15.71 | 9.23 | 18.45 | 17.81 | 8.16 | 9.29 | 6.34 | 10.42 | 1.71 | 110.22 | 13.60 | 10.05 | 3.93 | 0.96 | 11.98 | 9.12 | 2.79 | 7.58 | 17.29 | 3.28 | 2.90 | 4.13 |
|  |  | **M** | 3.50 | 9.18 | 18.54 | 17.68 | 8.21 | 9.49 | 6.31 | 0.00 | 2.00 | 111.78 | 13.69 | 10.00 | 3.90 | 0.00 | 11.93 | 8.99 | 2.72 | 7.64 | 0.00 | 0.00 | 0.00 | 0.00 |
|  |  | **MR** | 114.79 | 206.83 | 223.50 | 203.50 | 199.58 | 159.50 | 152.33 | 163.81 | 343.29 | 178.08 | 266.67 | 246.83 | 96.08 | 150.46 | 173.33 | 219.50 | 148.00 | 270.58 | 187.19 | 144.73 | 146.73 | 139.98 |
|  |  | **Var** | 438.74 | 0.55 | 0.23 | 0.79 | 0.15 | 2.73 | 0.14 | 290.95 | 0.57 | 13.00 | 0.30 | 0.24 | 0.08 | 2.88 | 0.53 | 0.18 | 0.35 | 0.13 | 938.55 | 35.02 | 27.13 | 55.59 |
|  |  | **Min** | 0.00 | 8.35 | 17.76 | 16.89 | 7.66 | 7.33 | 5.81 | 0.00 | 0.00 | 105.65 | 12.89 | 9.42 | 3.60 | 0.00 | 11.17 | 8.71 | 2.22 | 7.00 | 0.00 | 0.00 | 0.00 | 0.00 |
|  |  | **Max** | 64.00 | 10.52 | 18.99 | 19.19 | 8.78 | 11.56 | 6.82 | 46.00 | 2.00 | 113.23 | 14.20 | 10.78 | 4.40 | 4.00 | 12.89 | 9.68 | 3.55 | 7.93 | 74.88 | 15.72 | 13.46 | 20.00 |
|  |  | **IR** | 31.00 | 1.04 | 0.93 | 1.49 | 0.61 | 2.99 | 0.72 | 27.00 | 0.00 | 7.33 | 1.12 | 0.87 | 0.35 | 2.70 | 1.44 | 0.86 | 1.05 | 0.59 | 48.62 | 7.34 | 6.31 | 9.00 |
|  | **12** | **N** | 8 | 8 | 8 | 8 | 8 | 8 | 8 | 16 | 16 | 8 | 8 | 8 | 8 | 16 | 8 | 8 | 8 | 8 | 16 | 16 | 16 | 16 |
|  |  | $\bar{\boldsymbol{x}}$ | 70.38 | 6.43 | 14.40 | 14.21 | 5.58 | 5.97 | 4.91 | 16.00 | 0.89 | 103.26 | 9.56 | 7.40 | 3.63 | 1.71 | 12.50 | 7.43 | 1.86 | 5.93 | 19.20 | 15.18 | 13.93 | 36.63 |
|  |  | **M** | 65.50 | 6.79 | 0.49 | 0.45 | 0.08 | 0.05 | 0.14 | 11.50 | 0.70 | 100.88 | 9.37 | 7.49 | 3.50 | 1.50 | 12.42 | 7.50 | 1.86 | 5.96 | 18.13 | 12.38 | 11.06 | 28.00 |
|  |  | **MR** | 342.00 | 94.75 | 123.38 | 110.06 | 53.13 | 20.19 | 47.00 | 204.81 | 181.03 | 105.88 | 86.94 | 68.81 | 78.56 | 188.88 | 207.63 | 117.81 | 19.06 | 166.50 | 215.13 | 233.47 | 234.69 | 233.72 |
|  |  | **Var** | 151.98 | 1.22 | 0.70 | 0.67 | 0.28 | 0.23 | 0.37 | 335.47 | 0.86 | 22.31 | 0.17 | 0.17 | 0.18 | 3.21 | 0.19 | 0.14 | 0.01 | 0.01 | 394.40 | 253.04 | 215.12 | 1488.65 |
|  |  | **Min** | 55.00 | 4.98 | 16.02 | 15.51 | 6.13 | 6.62 | 5.83 | 0.00 | 0.00 | 99.29 | 9.05 | 6.67 | 3.00 | 0.00 | 11.98 | 6.81 | 1.76 | 5.75 | 0.00 | 0.00 | 0.00 | 0.00 |
|  |  | **Max** | 90.00 | 7.69 | 1.90 | 1.79 | 0.79 | 0.73 | 1.14 | 57.00 | 2.00 | 112.33 | 10.07 | 7.93 | 4.20 | 4.20 | 13.34 | 7.97 | 1.99 | 6.07 | 41.04 | 35.76 | 33.27 | 88.00 |
|  |  | **IR** | 21.75 | 2.12 | -0.06 | -0.84 | -0.54 | 0.98 | 0.15 | 28.00 | 1.80 | 7.20 | 0.77 | 0.64 | 0.70 | 3.35 | 0.65 | 0.59 | 0.18 | 0.22 | 38.24 | 31.29 | 28.59 | 73.75 |
|  | **10** | **N** | 16 |  | 16 | 16 | 16 | 16 | 16 | 23 | 24 | 16 | 16 | 16 | 16 | 24 | 16 | 16 | 16 | 16 | 24 | 24 | 24 | 24 |
|  |  | $\bar{\boldsymbol{x}}$ | 10.38 |  | 19.21 | 19.53 | 15.96 | 12.80 | 10.92 | 4.04 | 1.09 | 174.36 | 8.34 | 7.28 | 10.09 | 6.73 | 9.62 | 6.13 | 4.00 | 5.16 | 13.03 | 3.12 | 2.04 | 9.42 |
|  |  | **M** | 11.00 |  | 19.38 | 19.62 | 16.08 | 12.52 | 10.76 | 3.00 | 1.40 | 172.35 | 8.33 | 7.07 | 9.90 | 9.20 | 9.73 | 6.21 | 4.05 | 5.10 | 18.14 | 1.66 | 0.53 | 2.50 |
|  |  | **MR** | 123.16 |  | 241.34 | 244.22 | 258.47 | 249.75 | 242.78 | 164.04 | 191.60 | 300.19 | 22.31 | 54.63 | 298.47 | 367.58 | 49.13 | 35.31 | 261.53 | 68.34 | 190.94 | 181.75 | 180.52 | 188.13 |
|  |  | **Var** | 20.25 |  | 0.61 | 0.55 | 1.08 | 1.73 | 0.45 | 17.41 | 0.68 | 245.08 | 0.11 | 0.75 | 0.94 | 24.21 | 0.11 | 0.09 | 0.19 | 0.04 | 91.52 | 68.99 | 36.48 | 681.12 |
|  |  | **Min** | 3.00 |  | 17.72 | 18.11 | 13.87 | 11.03 | 10.15 | 0.00 | 0.00 | 148.50 | 7.88 | 6.61 | 8.80 | 0.00 | 9.02 | 5.66 | 2.47 | 4.88 | 0.00 | 0.00 | 0.00 | 0.00 |
|  |  | **Max** | 21.00 |  | 20.72 | 20.64 | 17.56 | 15.86 | 12.39 | 16.00 | 2.00 | 214.20 | 9.04 | 9.94 | 11.80 | 11.80 | 10.09 | 6.49 | 4.37 | 5.56 | 23.60 | 41.69 | 30.04 | 130.00 |
|  |  | **IR** | 5.50 |  | 1.08 | 1.32 | 1.67 | 1.64 | 1.09 | 7.00 | 1.80 | 12.92 | 0.56 | 0.49 | 1.75 | 10.20 | 0.54 | 0.59 | 0.31 | 0.27 | 19.65 | 2.32 | 1.53 | 8.25 |
|  | **11** | **N** | 8 |  |  |  |  |  |  | 24 | 24 |  |  |  |  | 24 |  |  |  |  | 24 | 24 | 24 | 24 |
|  |  | $\bar{\boldsymbol{x}}$ | 2.88 |  |  |  |  |  |  | 0.04 | 0.04 |  |  |  |  |  |  |  |  |  |  |  |  |  |
|  |  | **M** | 2.50 |  |  |  |  |  |  | 0.00 | 0.00 |  |  |  |  |  |  |  |  |  |  |  |  |  |
|  |  | **MR** | 47.94 |  |  |  |  |  |  | 78.88 | 59.48 |  |  |  |  | 101.50 |  |  |  |  | 100.50 | 100.50 | 100.50 | 100.50 |
|  |  | **Var** | 1.27 |  |  |  |  |  |  | 0.04 | 0.04 |  |  |  |  |  |  |  |  |  |  |  |  |  |
|  |  | **Min** | 2.00 |  |  |  |  |  |  | 0.00 | 0.00 |  |  |  |  |  |  |  |  |  |  |  |  |  |
|  |  | **Max** | 5.00 |  |  |  |  |  |  | 1.00 | 1.00 |  |  |  |  |  |  |  |  |  |  |  |  |  |
|  |  | **IR** | 1.75 |  |  |  |  |  |  | 0.00 | 0.00 |  |  |  |  |  |  |  |  |  |  |  |  |  |
|  | **13** | **N** | 15 | 15 | 15 | 15 | 15 | 15 | 15 | 24 | 24 | 15 | 15 | 15 | 15 | 24 | 15 | 15 | 15 | 15 | 24 | 24 | 24 | 24 |
|  |  | $\bar{\boldsymbol{x}}$ | 64.20 | 5.31 | 11.04 | 10.28 | 5.05 | 6.58 | 5.45 | 32.83 | 3.65 | 97.17 | 22.33 | 8.47 | 2.15 | 1.33 | 22.17 | 12.28 | 5.19 | 5.44 | 71.08 | 79.14 | 74.00 | 65.33 |
|  |  | **M** | 60.00 | 5.39 | 0.33 | 0.48 | 0.49 | 0.38 | 0.22 | 47.50 | 5.00 | 97.67 | 22.22 | 8.33 | 2.20 | 2.00 | 22.16 | 12.36 | 5.16 | 5.44 | 109.43 | 112.12 | 104.80 | 93.50 |
|  |  | **MR** | 326.40 | 35.53 | 26.80 | 16.60 | 36.90 | 54.73 | 93.37 | 274.67 | 295.13 | 51.07 | 303.47 | 140.10 | 18.70 | 176.17 | 301.30 | 302.37 | 300.33 | 111.10 | 353.79 | 338.81 | 339.06 | 286.96 |
|  |  | **Var** | 99.74 | 0.35 | 0.58 | 0.70 | 0.70 | 0.61 | 0.47 | 705.62 | 9.01 | 20.41 | 0.36 | 0.60 | 0.02 | 1.11 | 0.20 | 0.06 | 0.04 | 0.01 | 3178.70 | 4104.26 | 3583.80 | 2784.58 |
|  |  | **Min** | 54.00 | 4.40 | 12.27 | 12.23 | 7.17 | 7.91 | 6.48 | 0.00 | 0.00 | 90.85 | 21.29 | 7.47 | 2.00 | 0.00 | 21.39 | 11.72 | 4.95 | 5.30 | 0.00 | 0.00 | 0.00 | 0.00 |
|  |  | **Max** | 88.00 | 6.07 | 1.95 | 2.48 | 2.59 | 2.15 | 1.75 | 66.00 | 7.40 | 106.98 | 23.57 | 10.32 | 2.40 | 2.40 | 23.10 | 12.57 | 5.56 | 5.58 | 123.12 | 164.10 | 152.66 | 131.00 |
|  |  | **IR** | 16.00 | 1.21 | -0.16 | 0.73 | 1.72 | -0.07 | -0.18 | 55.75 | 6.00 | 6.13 | 0.67 | 1.02 | 0.20 | 2.20 | 0.35 | 0.31 | 0.32 | 0.14 | 115.91 | 127.67 | 121.03 | 109.50 |
| **Gh** | **1** | **N** | 24 |  |  |  |  |  |  | 24 | 24 |  |  |  |  | 24 |  |  |  |  | 24 | 24 | 24 | 24 |
|  |  | $\bar{\boldsymbol{x}}$ | 6.63 |  |  |  |  |  |  |  |  |  |  |  |  |  |  |  |  |  |  |  |  |  |
|  |  | **M** | 4.00 |  |  |  |  |  |  |  |  |  |  |  |  |  |  |  |  |  |  |  |  |  |
|  |  | **MR** | 78.50 |  |  |  |  |  |  | 75.50 | 57.00 |  |  |  |  | 101.50 |  |  |  |  | 100.50 | 100.50 | 100.50 | 100.50 |
|  |  | **Var** | 51.98 |  |  |  |  |  |  |  |  |  |  |  |  |  |  |  |  |  |  |  |  |  |
|  |  | **Min** | 1.00 |  |  |  |  |  |  |  |  |  |  |  |  |  |  |  |  |  |  |  |  |  |
|  |  | **Max** | 31.00 |  |  |  |  |  |  |  |  |  |  |  |  |  |  |  |  |  |  |  |  |  |
|  |  | **IR** | 6.75 |  |  |  |  |  |  |  |  |  |  |  |  |  |  |  |  |  |  |  |  |  |
|  | **2** | **N** | 23 | 17 | 17 | 17 | 17 | 17 | 17 | 24 | 24 | 23 | 23 | 23 | 23 | 24 | 23 | 23 | 23 | 23 | 24 | 24 | 24 | 24 |
|  |  | $\bar{\boldsymbol{x}}$ | 132.39 | 8.33 | 17.97 | 17.77 | 6.47 | 8.44 | 6.68 | 105.21 | 1.60 | 121.30 | 12.53 | 10.56 | 6.05 | 5.45 | 12.36 | 9.56 | 2.53 | 8.15 | 64.75 | 95.90 | 89.18 | 132.13 |
|  |  | **M** | 105.00 | 8.41 | 0.28 | 0.50 | 0.17 | 2.16 | 0.98 | 85.50 | 1.60 | 119.52 | 12.24 | 10.69 | 6.00 | 5.70 | 12.40 | 9.51 | 2.51 | 8.12 | 66.13 | 88.65 | 82.30 | 126.00 |
|  |  | **MR** | 419.39 | 175.74 | 218.21 | 212.03 | 117.71 | 157.47 | 185.21 | 452.67 | 253.13 | 239.57 | 224.52 | 284.57 | 228.11 | 406.44 | 197.61 | 247.37 | 132.28 | 310.87 | 413.56 | 429.02 | 428.73 | 404.00 |
|  |  | **Var** | 2496.52 | 1.39 | 0.53 | 0.71 | 0.41 | 1.47 | 0.99 | 2768.26 | 0.18 | 60.71 | 1.34 | 0.21 | 0.49 | 1.99 | 0.57 | 0.19 | 0.02 | 0.10 | 207.93 | 4038.58 | 3549.25 | 7726.90 |
|  |  | **Min** | 72.00 | 6.57 | 19.54 | 20.11 | 7.28 | 12.13 | 8.66 | 0.00 | 0.00 | 111.68 | 10.95 | 9.53 | 4.60 | 0.00 | 9.77 | 8.90 | 2.25 | 7.67 | 0.00 | 0.00 | 0.00 | 0.00 |
|  |  | **Max** | 219.00 | 10.27 | 2.16 | 3.48 | 1.41 | 4.79 | 2.78 | 196.00 | 2.20 | 139.90 | 15.53 | 11.17 | 7.40 | 7.20 | 13.47 | 10.28 | 2.76 | 8.68 | 77.67 | 220.15 | 205.10 | 311.00 |
|  |  | **IR** | 97.00 | 2.14 | 0.63 | 0.86 | -0.37 | 0.63 | 0.51 | 97.75 | 0.40 | 10.38 | 1.46 | 0.66 | 1.00 | 1.25 | 0.74 | 0.84 | 0.20 | 0.59 | 4.29 | 99.09 | 87.83 | 149.50 |
|  | **3** | **N** | 24 | 20 | 20 | 20 | 20 | 20 | 20 | 24 | 24 | 24 | 24 | 24 | 24 | 24 | 24 | 24 | 24 | 24 | 24 | 24 | 24 | 24 |
|  |  | $\bar{\boldsymbol{x}}$ | 40.96 | 7.84 | 15.98 | 15.88 | 7.31 | 8.36 | 6.11 | 29.46 | 1.70 | 106.41 | 12.04 | 9.45 | 4.22 | 4.06 | 11.71 | 9.09 | 2.42 | 7.28 | 67.42 | 22.06 | 19.59 | 28.79 |
|  |  | **M** | 40.00 | 7.59 | 1.56 | 0.98 | 0.12 | 0.38 | 0.21 | 27.00 | 1.60 | 106.64 | 11.95 | 9.56 | 4.20 | 4.00 | 11.85 | 9.06 | 2.43 | 7.33 | 67.85 | 23.93 | 22.55 | 34.00 |
|  |  | **MR** | 259.98 | 160.08 | 166.53 | 158.65 | 172.35 | 146.33 | 148.40 | 322.63 | 272.35 | 140.00 | 208.35 | 201.98 | 121.65 | 320.33 | 155.71 | 214.92 | 97.75 | 240.75 | 432.04 | 328.25 | 328.46 | 296.31 |
|  |  | **Var** | 255.96 | 1.12 | 1.25 | 0.99 | 0.35 | 0.62 | 0.46 | 194.00 | 0.04 | 17.01 | 0.80 | 0.71 | 0.12 | 0.11 | 0.53 | 0.51 | 0.01 | 0.02 | 5.82 | 35.36 | 48.36 | 98.26 |
|  |  | **Min** | 22.00 | 6.70 | 20.11 | 18.23 | 8.11 | 9.59 | 7.10 | 13.00 | 1.40 | 98.72 | 10.37 | 7.59 | 3.60 | 3.60 | 10.09 | 7.93 | 2.14 | 6.99 | 63.15 | 9.95 | 6.65 | 10.00 |
|  |  | **Max** | 76.00 | 10.61 | 5.56 | 3.37 | 1.24 | 2.80 | 1.39 | 62.00 | 2.00 | 114.28 | 13.69 | 10.93 | 4.80 | 4.60 | 12.60 | 11.13 | 2.62 | 7.54 | 71.65 | 33.21 | 30.42 | 42.00 |
|  |  | **IR** | 21.00 | 1.09 | 1.03 | 0.70 | -0.11 | -1.27 | 0.10 | 16.00 | 0.20 | 4.69 | 1.46 | 1.12 | 0.40 | 0.60 | 1.06 | 0.83 | 0.14 | 0.25 | 4.16 | 10.54 | 12.38 | 17.75 |
|  | **4** | **N** | 22 | 22 | 22 | 22 | 22 | 22 | 22 | 24 | 24 | 22 | 22 | 22 | 22 | 24 | 22 | 22 | 22 | 22 | 24 | 24 | 24 | 24 |
|  |  | $\bar{\boldsymbol{x}}$ | 111.1818 | 6.6382 | 12.3689 | 12.4016 | 6.2894 | 7.9525 | 5.1158 | 86.3333 | 1.5583 | 102.2091 | 8.8164 | 8.6659 | 5.9545 | 5.2833 | 10.2732 | 6.7809 | 2.4555 | 4.7477 | 24.9554 | 55.5442 | 54.2992 | 197.4583 |
|  |  | **M** | 97 | 6.745 | 0.653 | 0.51 | 0.1 | 0.305 | 0.203 | 84 | 1.6 | 103.42 | 8.91 | 8.205 | 6.1 | 5.8 | 10.4 | 6.805 | 2.46 | 4.775 | 26.71 | 53.495 | 52.795 | 171.5 |
|  |  | **MR** | 400.61 | 102.16 | 60.89 | 58.61 | 96.34 | 119.14 | 55.34 | 429.92 | 256.19 | 99.07 | 50.18 | 154.20 | 216.23 | 392.35 | 85.50 | 76.82 | 108.11 | 22.52 | 262.60 | 383.46 | 387.71 | 429.23 |
|  |  | **Var** | 2393.108 | 1.15 | 0.80836 | 0.71403 | 0.3161 | 0.55211 | 0.45029 | 1981.362 | 0.264 | 34.487 | 0.681 | 1.78 | 1.535 | 4.257 | 0.337 | 0.141 | 0.033 | 0.016 | 81.123 | 1340.364 | 1287.508 | 19244.607 |
|  |  | **Min** | 41 | 4.66 | 14.09 | 13.71 | 6.89 | 9.15 | 6.73 | 0 | 0 | 91.95 | 7.05 | 6.62 | 3 | 0 | 8.91 | 5.68 | 2.15 | 4.45 | 0 | 0 | 0 | 0 |
|  |  | **Max** | 271 | 8.9 | 3.16 | 2.96 | 1.22 | 1.9 | 2.04 | 215 | 2 | 110.63 | 10.49 | 11.37 | 7.4 | 7.4 | 11.12 | 7.45 | 2.83 | 4.99 | 36.62 | 132.72 | 128.69 | 547 |
|  |  | **IR** | 37 | 1.31 | -0.261 | -0.931 | -0.902 | 0.001 | 1.5 | 43 | 0.2 | 10.9 | 1.2 | 1.74 | 1.8 | 2.15 | 0.66 | 0.41 | 0.17 | 0.19 | 6.3 | 47.18 | 46.57 | 170.25 |
|  | **5** | **N** | 24 | 6 | 6 | 6 | 6 | 6 | 6 | 24 | 24 | 24 | 24 | 24 | 24 | 24 | 24 | 24 | 24 | 24 | 24 | 24 | 24 | 24 |
|  |  | $\bar{\boldsymbol{x}}$ | 56.83 | 6.22 | 11.33 | 11.93 | 6.21 | 7.27 | 5.95 | 45.88 | 1.58 | 107.55 | 11.45 | 9.48 | 4.63 | 4.50 | 12.31 | 8.31 | 2.58 | 7.09 | 50.41 | 56.01 | 52.30 | 103.25 |
|  |  | **M** | 50.50 | 6.48 | 5.71 | 3.94 | 0.06 | 0.37 | 0.04 | 39.00 | 1.60 | 107.18 | 11.52 | 9.46 | 4.60 | 4.50 | 12.23 | 8.26 | 2.61 | 7.10 | 50.48 | 60.46 | 56.36 | 114.00 |
|  |  | **MR** | 298.92 | 76.50 | 95.75 | 93.33 | 100.83 | 103.25 | 137.00 | 366.94 | 230.02 | 147.63 | 182.04 | 199.15 | 152.77 | 353.75 | 192.42 | 170.04 | 153.69 | 222.10 | 354.50 | 398.71 | 402.42 | 392.27 |
|  |  | **Var** | 867.36 | 0.41 | 2.39 | 1.98 | 0.25 | 0.61 | 0.20 | 690.55 | 0.05 | 49.86 | 0.55 | 0.13 | 0.34 | 0.32 | 0.28 | 0.08 | 0.02 | 0.02 | 3.28 | 894.91 | 732.77 | 2723.41 |
|  |  | **Min** | 21.00 | 5.38 | 17.30 | 16.75 | 6.69 | 8.63 | 6.41 | 11.00 | 1.20 | 97.56 | 9.30 | 8.71 | 3.40 | 3.40 | 11.33 | 7.90 | 2.09 | 6.59 | 46.88 | 9.28 | 9.28 | 19.00 |
|  |  | **Max** | 118.00 | 6.82 | 7.04 | 5.48 | 0.67 | 1.43 | 0.43 | 98.00 | 2.00 | 120.67 | 12.39 | 10.35 | 5.80 | 5.40 | 13.51 | 8.95 | 2.75 | 7.28 | 53.63 | 111.53 | 97.65 | 191.00 |
|  |  | **IR** | 46.50 | 1.31 | -0.08 | -0.08 | -1.41 | 0.00 | 0.48 | 43.75 | 0.40 | 13.15 | 0.85 | 0.29 | 0.80 | 1.00 | 0.84 | 0.41 | 0.12 | 0.17 | 2.43 | 47.30 | 44.88 | 87.75 |
|  | **6** | **N** | 24 | 4 | 4 | 4 | 4 | 4 | 4 | 24 | 24 | 24 | 24 | 24 | 24 | 24 | 24 | 24 | 24 | 24 | 24 | 24 | 24 | 24 |
|  |  | $\bar{\boldsymbol{x}}$ | 40.04 | 7.33 | 8.30 | 9.29 | 4.71 | 6.38 | 5.81 | 26.50 | 1.82 | 102.76 | 10.64 | 9.61 | 3.52 | 3.42 | 14.77 | 7.94 | 2.67 | 6.34 | 63.29 | 27.43 | 25.45 | 36.21 |
|  |  | **M** | 28.00 | 7.12 | 2.73 | 1.34 | 1.71 | 0.42 | 0.17 | 17.00 | 1.80 | 103.93 | 10.76 | 9.69 | 3.40 | 3.30 | 15.26 | 8.22 | 2.67 | 6.34 | 62.50 | 13.72 | 12.45 | 19.00 |
|  |  | **MR** | 246.98 | 123.38 | 26.38 | 27.38 | 118.13 | 78.13 | 159.00 | 297.52 | 321.19 | 113.25 | 139.38 | 214.81 | 81.29 | 283.56 | 254.48 | 147.98 | 170.54 | 192.15 | 398.56 | 308.54 | 311.90 | 288.00 |
|  |  | **Var** | 681.87 | 5.23 | 1.65 | 1.16 | 1.31 | 0.64 | 0.42 | 589.65 | 0.02 | 207.74 | 1.25 | 0.79 | 0.74 | 0.71 | 2.56 | 0.54 | 0.04 | 0.04 | 20.26 | 979.82 | 954.50 | 1833.56 |
|  |  | **Min** | 15.00 | 5.02 | 12.90 | 12.84 | 8.47 | 7.93 | 7.09 | 1.00 | 1.40 | 80.19 | 9.02 | 7.34 | 1.80 | 1.80 | 11.36 | 6.44 | 2.32 | 6.02 | 56.87 | 1.28 | 1.00 | 2.00 |
|  |  | **Max** | 101.00 | 10.06 | 3.57 | 2.49 | 2.93 | 1.46 | 0.84 | 88.00 | 2.00 | 128.37 | 12.75 | 10.66 | 5.20 | 5.20 | 16.83 | 8.75 | 2.97 | 6.74 | 72.48 | 107.69 | 106.82 | 151.00 |
|  |  | **IR** | 36.00 | 4.37 | 0.40 | 1.88 | 0.72 | -1.62 | 2.00 | 27.75 | 0.20 | 21.59 | 2.07 | 1.28 | 1.15 | 1.10 | 1.05 | 1.34 | 0.35 | 0.31 | 4.92 | 30.39 | 29.37 | 34.00 |
|  | **7** | **N** | 16 | 11 |  | 11 | 11 | 11 | 11 | 16 | 16 | 16 | 16 | 16 | 16 | 16 | 16 | 16 | 16 | 16 | 16 | 16 | 16 | 16 |
|  |  | $\bar{\boldsymbol{x}}$ | 97.44 | 6.84 | 12.14 | 13.34 | 7.68 | 8.43 | 6.04 | 83.81 | 1.74 | 105.42 | 9.93 | 7.40 | 6.25 | 6.18 | 9.99 | 7.13 | 2.84 | 5.50 | 28.79 | 56.00 | 52.96 | 182.69 |
|  |  | **M** | 85.50 | 6.29 | 4.20 | 4.27 | 0.40 | 0.24 | 0.36 | 69.00 | 1.80 | 107.58 | 10.07 | 7.28 | 6.40 | 6.40 | 9.95 | 7.19 | 2.82 | 5.51 | 28.00 | 44.05 | 42.92 | 159.00 |
|  |  | **MR** | 368.91 | 109.73 | 85.14 | 114.09 | 196.32 | 150.68 | 156.23 | 431.34 | 291.22 | 135.31 | 106.34 | 67.34 | 234.38 | 440.75 | 71.50 | 99.59 | 213.38 | 123.81 | 286.03 | 400.94 | 404.75 | 441.63 |
|  |  | **Var** | 3359.60 | 1.81 | 2.05 | 2.07 | 0.63 | 0.49 | 0.60 | 3424.03 | 0.05 | 32.04 | 0.85 | 0.15 | 0.56 | 0.54 | 0.09 | 0.08 | 0.01 | 0.01 | 8.62 | 1410.40 | 1226.68 | 15365.83 |
|  |  | **Min** | 35.00 | 5.55 | 16.61 | 17.92 | 9.29 | 9.84 | 7.29 | 28.00 | 1.40 | 89.17 | 8.26 | 6.99 | 4.00 | 4.00 | 9.41 | 6.67 | 2.67 | 5.37 | 24.48 | 15.89 | 15.79 | 58.00 |
|  |  | **Max** | 266.00 | 9.33 | 6.40 | 7.42 | 2.04 | 1.71 | 1.93 | 257.00 | 2.00 | 111.98 | 11.47 | 8.46 | 7.00 | 7.00 | 10.44 | 7.67 | 3.04 | 5.63 | 35.35 | 154.01 | 144.39 | 524.00 |
|  |  | **IR** | 63.75 | 2.01 | -0.14 | -0.56 | 0.57 | 0.98 | -0.37 | 69.00 | 0.50 | 7.94 | 1.14 | 0.45 | 0.55 | 0.60 | 0.45 | 0.40 | 0.15 | 0.10 | 3.77 | 43.20 | 42.98 | 140.50 |
|  | **8** | **N** | 16 |  | 11 |  |  |  | 16 | 16 |  |  |  |  |  | 16 |  |  |  |  | 16 | 16 | 16 | 16 |
|  |  | $\bar{\boldsymbol{x}}$ | 2.69 |  |  |  |  |  |  | 0.44 |  |  |  |  |  |  |  |  |  |  |  |  |  |  |
|  |  | **M** | 2.00 |  |  |  |  |  |  | 0.00 |  |  |  |  |  |  |  |  |  |  |  |  |  |  |
|  |  | **MR** | 37.56 |  |  |  |  |  |  | 89.31 |  |  |  |  |  | 101.50 |  |  |  |  | 100.50 | 100.50 | 100.50 | 100.50 |
|  |  | **Var** | 7.83 |  |  |  |  |  |  | 1.46 |  |  |  |  |  |  |  |  |  |  |  |  |  |  |
|  |  | **Min** | 1.00 |  |  |  |  |  |  | 0.00 |  |  |  |  |  |  |  |  |  |  |  |  |  |  |
|  |  | **Max** | 10.00 |  |  |  |  |  |  | 4.00 |  |  |  |  |  |  |  |  |  |  |  |  |  |  |
|  |  | **IR** | 1.75 |  |  |  |  |  |  | 0.00 |  |  |  |  |  |  |  |  |  |  |  |  |  |  |
|  | **9** | **N** | 24 | 16 | 16 | 16 | 16 | 16 |  | 24 | 24 | 16 | 16 | 16 | 16 | 24 | 16 | 16 | 16 | 16 | 24 | 24 | 24 | 24 |
|  |  | $\bar{\boldsymbol{x}}$ | 67.21 | 6.79 | 17.10 | 16.81 | 8.06 | 9.46 | 5.94 | 41.50 | 1.23 | 109.51 | 12.12 | 10.73 | 4.31 | 2.83 | 12.41 | 9.58 | 3.33 | 7.73 | 44.07 | 39.62 | 36.14 | 57.54 |
|  |  | **M** | 42.00 | 6.72 | 0.47 | 1.09 | 0.39 | 0.79 | 0.08 | 14.00 | 1.60 | 111.07 | 11.93 | 10.54 | 4.30 | 3.90 | 12.67 | 9.45 | 3.33 | 7.77 | 66.93 | 10.70 | 7.64 | 11.00 |
|  |  | **MR** | 308.73 | 112.41 | 192.38 | 190.56 | 204.06 | 192.63 | 130.31 | 281.06 | 219.38 | 166.94 | 213.06 | 289.47 | 130.19 | 258.33 | 202.31 | 249.38 | 244.09 | 286.47 | 321.88 | 280.23 | 279.13 | 266.48 |
|  |  | **Var** | 2293.48 | 0.45 | 0.69 | 1.04 | 0.62 | 0.89 | 0.29 | 2768.00 | 0.67 | 41.44 | 0.47 | 0.34 | 0.41 | 4.44 | 0.62 | 0.11 | 0.05 | 0.02 | 1052.30 | 2399.58 | 2065.41 | 5590.26 |
|  |  | **Min** | 17.00 | 5.71 | 18.96 | 18.97 | 9.32 | 11.22 | 6.62 | 0.00 | 0.00 | 97.93 | 11.27 | 10.02 | 3.20 | 0.00 | 11.15 | 9.08 | 3.06 | 7.45 | 0.00 | 0.00 | 0.00 | 0.00 |
|  |  | **Max** | 197.00 | 8.07 | 2.33 | 3.55 | 2.38 | 2.93 | 1.06 | 189.00 | 2.00 | 118.69 | 13.34 | 11.94 | 5.40 | 5.40 | 13.46 | 10.06 | 3.67 | 7.93 | 70.49 | 142.24 | 128.91 | 235.00 |
|  |  | **IR** | 83.25 | 1.08 | 0.68 | -0.31 | -0.63 | -0.03 | 0.09 | 92.75 | 1.80 | 11.18 | 1.33 | 0.93 | 0.85 | 4.40 | 1.36 | 0.59 | 0.45 | 0.14 | 68.43 | 90.82 | 86.02 | 125.50 |
|  | **12** | **N** | 24 | 24 | 24 | 24 | 24 | 24 | 24 | 24 | 24 | 24 | 24 | 24 | 24 | 24 | 24 | 24 | 24 | 24 | 24 | 24 | 24 | 24 |
|  |  | $\bar{\boldsymbol{x}}$ | 57.17 | 6.66 | 14.46 | 14.16 | 5.76 | 6.75 | 4.89 | 44.04 | 1.68 | 109.62 | 9.76 | 7.36 | 4.23 | 4.23 | 12.32 | 7.70 | 1.87 | 5.89 | 37.52 | 29.26 | 26.17 | 72.29 |
|  |  | **M** | 59.00 | 6.73 | 0.55 | 0.96 | 0.11 | 0.29 | 0.12 | 38.00 | 1.70 | 109.66 | 9.67 | 7.53 | 4.20 | 4.20 | 12.27 | 7.66 | 1.86 | 5.87 | 37.49 | 17.05 | 16.24 | 43.00 |
|  |  | **MR** | 306.08 | 106.65 | 117.35 | 106.19 | 60.38 | 56.98 | 32.69 | 364.81 | 266.38 | 169.54 | 99.60 | 69.83 | 124.83 | 338.17 | 193.48 | 133.79 | 21.46 | 163.96 | 324.08 | 325.73 | 327.06 | 343.42 |
|  |  | **Var** | 369.45 | 0.48 | 0.74 | 0.98 | 0.33 | 0.54 | 0.35 | 461.52 | 0.05 | 32.28 | 0.36 | 0.29 | 0.28 | 0.28 | 0.12 | 0.09 | 0.01 | 0.02 | 2.95 | 764.43 | 642.70 | 4857.52 |
|  |  | **Min** | 24.00 | 5.18 | 16.15 | 16.21 | 6.95 | 8.06 | 5.68 | 15.00 | 1.20 | 101.06 | 8.26 | 6.00 | 3.00 | 3.00 | 11.68 | 7.10 | 1.75 | 5.63 | 34.14 | 3.56 | 1.70 | 6.00 |
|  |  | **Max** | 102.00 | 7.70 | 3.00 | 4.10 | 1.46 | 1.93 | 1.20 | 91.00 | 2.00 | 120.01 | 10.95 | 8.24 | 5.20 | 5.20 | 12.95 | 8.33 | 2.00 | 6.14 | 41.31 | 126.80 | 115.09 | 306.00 |
|  |  | **IR** | 24.25 | 1.11 | 0.08 | -0.53 | 1.37 | 0.36 | 0.39 | 34.75 | 0.35 | 9.06 | 0.84 | 0.47 | 0.60 | 0.60 | 0.62 | 0.36 | 0.09 | 0.20 | 2.06 | 31.05 | 30.07 | 96.25 |
|  | **10** | **N** | 23 |  | 23 | 23 | 23 | 23 | 23 | 24 | 24 | 23 | 23 | 23 | 23 | 24 | 23 | 23 | 23 | 23 | 24 | 24 | 24 | 24 |
|  |  | $\bar{\boldsymbol{x}}$ | 87.78 |  | 17.02 | 17.66 | 15.43 | 12.28 | 10.69 | 68.83 | 1.57 | 172.32 | 8.97 | 6.87 | 10.23 | 9.79 | 9.39 | 6.11 | 3.96 | 5.21 | 19.47 | 55.46 | 51.92 | 258.54 |
|  |  | **M** | 95.00 |  | 8.00 | 6.92 | 0.90 | 4.55 | 1.09 | 71.50 | 1.60 | 171.19 | 8.90 | 6.87 | 10.00 | 9.90 | 9.33 | 6.06 | 4.05 | 5.19 | 19.83 | 41.75 | 38.12 | 163.00 |
|  |  | **MR** | 373.52 |  | 207.24 | 217.26 | 257.85 | 249.83 | 246.67 | 413.94 | 246.94 | 297.52 | 56.07 | 25.13 | 298.17 | 483.60 | 41.52 | 36.04 | 259.15 | 74.28 | 236.83 | 382.35 | 384.50 | 445.25 |
|  |  | **Var** | 826.36 |  | 2.83 | 2.63 | 0.95 | 2.13 | 1.04 | 990.15 | 0.17 | 148.07 | 0.35 | 0.04 | 2.10 | 6.35 | 0.40 | 0.08 | 0.22 | 0.02 | 21.58 | 2199.68 | 2076.02 | 57311.82 |
|  |  | **Min** | 36.00 |  | 21.88 | 21.71 | 17.06 | 18.08 | 12.75 | 0.00 | 0.00 | 152.02 | 7.92 | 6.36 | 8.60 | 0.00 | 8.09 | 5.54 | 2.40 | 4.91 | 0.00 | 0.00 | 0.00 | 0.00 |
|  |  | **Max** | 133.00 |  | 8.91 | 6.84 | 3.75 | 8.99 | 3.76 | 116.00 | 2.00 | 210.78 | 10.16 | 7.21 | 13.80 | 13.80 | 10.42 | 6.74 | 4.41 | 5.49 | 28.22 | 205.05 | 200.00 | 1020.00 |
|  |  | **IR** | 48.00 |  | -0.39 | -0.46 | -1.27 | 0.56 | -0.47 | 49.00 | 0.40 | 12.01 | 0.97 | 0.31 | 2.20 | 2.10 | 0.91 | 0.29 | 0.28 | 0.25 | 1.74 | 48.18 | 45.55 | 238.25 |
|  | **11** | **N** | 24 |  | 16 | 16 | 16 | 16 | 16 | 24 | 24 | 16 | 16 | 16 | 16 | 24 | 16 | 16 | 16 | 16 | 24 | 24 | 24 | 24 |
|  |  | $\bar{\boldsymbol{x}}$ | 13.79 |  | 19.77 | 19.70 | 14.53 | 12.24 | 13.39 | 9.50 | 1.15 | 174.37 | 9.99 | 7.89 | 10.85 | 7.22 | 8.74 | 5.78 | 2.73 | 4.94 | 10.95 | 8.12 | 7.32 | 44.96 |
|  |  | **M** | 14.50 |  | 0.77 | 0.62 | 0.40 | 0.51 | 0.61 | 9.00 | 1.40 | 180.59 | 9.83 | 7.84 | 10.20 | 10.00 | 8.76 | 5.76 | 2.73 | 4.99 | 15.96 | 9.61 | 8.12 | 49.00 |
|  |  | **MR** | 130.83 |  | 260.38 | 254.69 | 240.56 | 251.44 | 272.31 | 205.17 | 176.77 | 306.81 | 108.00 | 107.72 | 307.59 | 373.42 | 13.88 | 15.28 | 188.75 | 40.16 | 173.77 | 211.92 | 217.00 | 272.63 |
|  |  | **Var** | 91.22 |  | 0.88 | 0.79 | 0.63 | 0.71 | 0.78 | 56.70 | 0.50 | 206.87 | 0.91 | 0.10 | 1.50 | 28.15 | 0.13 | 0.03 | 0.02 | 0.02 | 62.83 | 49.49 | 41.46 | 1653.09 |
|  |  | **Min** | 1.00 |  | 21.43 | 21.21 | 16.07 | 14.04 | 15.11 | 0.00 | 0.00 | 143.88 | 8.49 | 7.25 | 9.40 | 0.00 | 8.12 | 5.46 | 2.47 | 4.72 | 0.00 | 0.00 | 0.00 | 0.00 |
|  |  | **Max** | 28.00 |  | 3.28 | 2.72 | 2.55 | 2.99 | 2.50 | 22.00 | 2.00 | 189.50 | 11.66 | 8.46 | 13.40 | 13.40 | 9.45 | 6.10 | 2.94 | 5.15 | 17.38 | 23.71 | 21.43 | 138.00 |
|  |  | **IR** | 20.00 |  | -0.86 | -0.82 | 0.05 | -0.12 | 0.10 | 16.50 | 1.35 | 13.40 | 1.62 | 0.42 | 1.95 | 11.10 | 0.48 | 0.15 | 0.09 | 0.27 | 16.64 | 11.49 | 10.92 | 66.50 |
|  | **13** | **N** | 24 | 24 | 24 | 24 | 24 | 24 | 24 | 24 | 24 | 24 | 24 | 24 | 24 | 24 | 24 | 24 | 24 | 24 | 24 | 24 | 24 | 24 |
|  |  | $\bar{\boldsymbol{x}}$ | 196.58 | 5.19 | 10.65 | 11.04 | 5.04 | 5.95 | 5.38 | 166.29 | 5.84 | 97.20 | 22.79 | 9.38 | 2.22 | 2.21 | 22.83 | 12.64 | 5.45 | 5.39 | 116.29 | 260.55 | 246.51 | 210.58 |
|  |  | **M** | 110.00 | 5.25 | 1.82 | 1.41 | 0.52 | 0.44 | 0.12 | 96.00 | 5.90 | 96.69 | 22.64 | 9.49 | 2.20 | 2.20 | 22.86 | 12.64 | 5.45 | 5.41 | 116.39 | 251.90 | 238.78 | 205.00 |
|  |  | **MR** | 428.50 | 29.54 | 29.79 | 33.25 | 31.42 | 26.96 | 74.48 | 487.00 | 438.00 | 49.58 | 312.46 | 196.67 | 23.75 | 227.56 | 313.81 | 313.15 | 314.42 | 101.23 | 511.02 | 515.96 | 516.00 | 484.08 |
|  |  | **Var** | 18701.04 | 0.27 | 1.35 | 1.19 | 0.72 | 0.67 | 0.34 | 12585.69 | 1.08 | 12.22 | 0.29 | 0.56 | 0.05 | 0.05 | 0.27 | 0.13 | 0.01 | 0.02 | 13.29 | 1870.97 | 1764.90 | 1150.86 |
|  |  | **Min** | 83.00 | 4.22 | 13.01 | 12.89 | 7.48 | 7.74 | 6.05 | 71.00 | 3.60 | 89.07 | 21.75 | 7.15 | 1.60 | 1.60 | 21.84 | 12.12 | 5.27 | 5.10 | 108.55 | 183.98 | 173.24 | 152.00 |
|  |  | **Max** | 415.00 | 6.07 | 4.83 | 4.12 | 2.98 | 2.59 | 1.17 | 339.00 | 7.40 | 104.98 | 23.86 | 10.63 | 2.60 | 2.60 | 23.81 | 13.30 | 5.63 | 5.55 | 123.70 | 364.96 | 346.60 | 282.00 |
|  |  | **IR** | 269.75 | 0.91 | -0.87 | -1.16 | 2.17 | 0.57 | -0.23 | 230.00 | 1.55 | 4.60 | 0.79 | 0.96 | 0.40 | 0.40 | 0.88 | 0.63 | 0.20 | 0.19 | 3.83 | 57.63 | 59.04 | 44.00 |

**Supporting Table S5. Statistical characterization of plant phenological stages.** Landraces 1-8 and 12 are *P. vulgaris* landraces; 9 is the commercial *P. vulgaris* cultivar; 10 and 11 the *V. unguiculata* landraces; and 13 the *P. lunatus* landrace. T: treatment; L: landrace; S: Statistic: F, open field; Gh: greenhouse. $\bar{x}$: mean; M: median; Var: variance; MR: Mean Rank after Kruskal-Wallis test; IR: interquartile range.

| **T** | **L** | **S** | **P8** | **P9** | **P10** | **P12** | **P13** | **P19** | **P21** | **P51** | **P55** | **P59** | **P61** | **P65** | **P67** | **P69** | **P81** | **P85** | **P89** |
| --- | --- | --- | --- | --- | --- | --- | --- | --- | --- | --- | --- | --- | --- | --- | --- | --- | --- | --- | --- |
| **F** | **1** | **N** | 8 | 8 | 8 | 8 | 8 | 8 | 8 | 8 | 8 | 7 | 7 | 7 | 7 | 4 | 4 | 4 | 4 |
|  |  | $\bar{\boldsymbol{x}}$ |  |  |  | 16.13 | 24.25 | 55.75 | 42.13 | 67.88 | 73.75 | 78.00 | 83.43 | 100.86 | 131.86 | 87.00 | 101.75 | 128.00 | 178.75 |
|  |  | **M** |  |  |  | 18.00 | 24.00 | 56.00 | 42.00 | 65.00 | 73.00 | 72.00 | 86.00 | 91.00 | 133.00 | 83.00 | 95.00 | 132.00 | 178.50 |
|  |  | **MR** | 87.00 | 144.50 | 229.00 | 287.06 | 176.25 | 303.88 | 345.25 | 302.31 | 310.81 | 297.07 | 285.00 | 237.36 | 238.29 | 195.63 | 195.13 | 225.13 | 248.25 |
|  |  | **Var** |  |  |  | 6.70 | 2.21 | 9.36 | 1.55 | 80.13 | 94.21 | 122.67 | 131.29 | 672.81 | 2345.81 | 330.67 | 673.58 | 876.00 | 1616.92 |
|  |  | **Min** |  |  |  | 13.00 | 23.00 | 50.00 | 41.00 | 61.00 | 65.00 | 68.00 | 71.00 | 71.00 | 76.00 | 71.00 | 79.00 | 95.00 | 142.00 |
|  |  | **Max** |  |  |  | 18.00 | 27.00 | 60.00 | 44.00 | 86.00 | 92.00 | 98.00 | 102.00 | 142.00 | 202.00 | 111.00 | 138.00 | 153.00 | 216.00 |
|  |  | **IR** |  |  |  | 5.00 | 2.00 | 4.00 | 2.00 | 12.00 | 13.75 | 14.00 | 20.00 | 46.00 | 99.00 | 34.00 | 47.75 | 54.00 | 71.75 |
|  | **2** | **N** | 24 | 24 | 24 | 24 | 24 | 24 | 24 | 24 | 24 | 24 | 19 | 19 | 19 | 19 | 19 | 19 | 19 |
|  |  | $\bar{\boldsymbol{x}}$ | 8.21 | 10.88 | 13.08 | 17.17 | 26.25 | 58.04 | 43.58 | 68.79 | 73.58 | 79.17 | 80.00 | 93.42 | 114.79 | 86.16 | 99.21 | 109.74 | 132.05 |
|  |  | **M** | 8.00 | 11.00 | 13.00 | 18.00 | 26.00 | 58.00 | 41.00 | 70.00 | 75.00 | 79.00 | 82.00 | 95.00 | 125.00 | 86.00 | 97.00 | 107.00 | 138.00 |
|  |  | **MR** | 318.31 | 365.31 | 362.25 | 325.77 | 260.58 | 346.40 | 356.06 | 307.33 | 305.31 | 303.04 | 253.47 | 210.97 | 183.18 | 209.11 | 195.08 | 149.29 | 113.66 |
|  |  | **Var** | 4.00 | 5.42 | 6.43 | 10.15 | 11.33 | 24.30 | 14.78 | 49.39 | 64.60 | 95.97 | 88.89 | 150.59 | 469.29 | 71.14 | 180.06 | 249.54 | 334.72 |
|  |  | **Min** | 6.00 | 7.00 | 9.00 | 13.00 | 23.00 | 53.00 | 39.00 | 55.00 | 58.00 | 62.00 | 68.00 | 75.00 | 86.00 | 70.00 | 82.00 | 90.00 | 107.00 |
|  |  | **Max** | 13.00 | 16.00 | 19.00 | 23.00 | 34.00 | 71.00 | 53.00 | 79.00 | 90.00 | 100.00 | 100.00 | 121.00 | 161.00 | 102.00 | 133.00 | 142.00 | 169.00 |
|  |  | **IR** | 0.75 | 2.00 | 2.00 | 5.00 | 6.00 | 7.50 | 6.00 | 13.00 | 14.00 | 15.75 | 16.00 | 18.00 | 39.00 | 9.00 | 12.00 | 30.00 | 30.00 |
|  | **3** | **N** | 24 | 24 | 24 | 24 | 24 | 24 | 24 | 24 | 24 | 24 | 24 | 24 | 24 | 14 | 14 | 14 | 14 |
|  |  | $\bar{\boldsymbol{x}}$ | 6.88 | 8.17 | 10.58 | 13.71 | 25.42 | 56.88 | 41.38 | 62.79 | 67.96 | 73.46 | 76.79 | 91.75 | 111.75 | 88.79 | 94.79 | 120.57 | 143.00 |
|  |  | **M** | 7.00 | 8.00 | 11.00 | 14.00 | 25.00 | 57.00 | 41.00 | 61.00 | 66.50 | 71.50 | 74.00 | 86.00 | 100.00 | 83.00 | 91.00 | 127.50 | 165.50 |
|  |  | **MR** | 196.10 | 166.23 | 193.81 | 198.46 | 218.29 | 321.81 | 300.58 | 246.06 | 260.58 | 272.71 | 235.69 | 191.44 | 181.06 | 218.39 | 172.04 | 189.18 | 158.11 |
|  |  | **Var** | 0.90 | 1.28 | 1.38 | 0.22 | 10.69 | 20.11 | 12.51 | 42.00 | 45.35 | 50.69 | 63.65 | 397.50 | 1066.54 | 150.18 | 127.57 | 528.73 | 1698.77 |
|  |  | **Min** | 6.00 | 7.00 | 9.00 | 13.00 | 23.00 | 53.00 | 36.00 | 58.00 | 61.00 | 67.00 | 69.00 | 70.00 | 74.00 | 75.00 | 84.00 | 84.00 | 84.00 |
|  |  | **Max** | 9.00 | 11.00 | 13.00 | 14.00 | 34.00 | 71.00 | 50.00 | 86.00 | 92.00 | 100.00 | 107.00 | 146.00 | 172.00 | 111.00 | 128.00 | 161.00 | 189.00 |
|  |  | **IR** | 1.00 | 2.00 | 2.00 | 1.00 | 4.00 | 5.00 | 1.00 | 5.50 | 4.00 | 4.50 | 6.00 | 26.75 | 54.25 | 20.50 | 8.75 | 38.50 | 77.50 |
|  | **4** | **N** | 18 | 18 | 18 | 18 | 18 | 18 | 18 | 18 | 18 | 17 | 17 | 17 | 17 | 15 | 15 | 15 | 15 |
|  |  | $\bar{\boldsymbol{x}}$ | 10.00 | 12.50 | 15.00 | 22.44 | 31.28 | 59.89 | 44.78 | 74.06 | 79.39 | 86.24 | 90.06 | 112.59 | 144.00 | 110.13 | 120.80 | 130.40 | 164.60 |
|  |  | **M** | 9.00 | 11.00 | 13.00 | 23.00 | 31.50 | 61.00 | 44.00 | 73.00 | 77.00 | 84.00 | 88.00 | 111.00 | 149.00 | 111.00 | 128.00 | 135.00 | 169.00 |
|  |  | **MR** | 441.50 | 436.42 | 445.17 | 477.56 | 433.47 | 390.94 | 398.42 | 349.14 | 349.06 | 347.06 | 320.41 | 290.53 | 284.71 | 299.27 | 273.37 | 230.27 | 212.30 |
|  |  | **Var** | 4.94 | 6.62 | 8.47 | 12.73 | 12.92 | 13.99 | 13.60 | 90.88 | 97.90 | 123.19 | 161.18 | 517.88 | 1077.50 | 212.84 | 345.31 | 588.97 | 1248.26 |
|  |  | **Min** | 8.00 | 10.00 | 13.00 | 18.00 | 23.00 | 53.00 | 41.00 | 60.00 | 65.00 | 68.00 | 69.00 | 75.00 | 88.00 | 82.00 | 82.00 | 88.00 | 107.00 |
|  |  | **Max** | 13.00 | 16.00 | 19.00 | 27.00 | 36.00 | 65.00 | 53.00 | 86.00 | 92.00 | 98.00 | 111.00 | 149.00 | 202.00 | 128.00 | 146.00 | 161.00 | 211.00 |
|  |  | **IR** | 5.00 | 5.00 | 6.00 | 7.50 | 5.00 | 6.00 | 6.25 | 18.75 | 21.00 | 19.50 | 19.50 | 30.00 | 55.00 | 28.00 | 28.00 | 46.00 | 76.00 |
|  | **5** | **N** | 24 | 24 | 24 | 24 | 24 | 24 | 24 | 22 | 22 | 22 | 21 | 21 | 21 | 19 | 19 | 19 | 19 |
|  |  | $\bar{\boldsymbol{x}}$ | 7.13 | 8.50 | 9.63 | 13.63 | 29.00 | 52.08 | 42.33 | 57.45 | 61.91 | 66.91 | 68.48 | 77.00 | 94.24 | 78.05 | 85.63 | 91.63 | 112.79 |
|  |  | **M** | 7.00 | 8.00 | 9.00 | 13.00 | 29.00 | 52.00 | 42.00 | 58.00 | 61.00 | 68.00 | 69.00 | 75.00 | 95.00 | 76.00 | 84.00 | 90.00 | 111.00 |
|  |  | **MR** | 197.94 | 175.06 | 111.75 | 123.81 | 392.17 | 206.42 | 331.33 | 166.43 | 176.73 | 191.43 | 124.21 | 113.67 | 121.45 | 159.24 | 117.08 | 73.47 | 66.29 |
|  |  | **Var** | 2.46 | 3.13 | 2.85 | 2.85 | 6.09 | 18.08 | 8.93 | 6.26 | 7.61 | 7.42 | 7.16 | 26.50 | 151.79 | 27.50 | 30.58 | 46.14 | 324.95 |
|  |  | **Min** | 6.00 | 7.00 | 9.00 | 13.00 | 27.00 | 47.00 | 40.00 | 54.00 | 57.00 | 62.00 | 63.00 | 69.00 | 69.00 | 69.00 | 74.00 | 82.00 | 90.00 |
|  |  | **Max** | 11.00 | 13.00 | 14.00 | 18.00 | 34.00 | 60.00 | 49.00 | 61.00 | 68.00 | 72.00 | 74.00 | 88.00 | 121.00 | 90.00 | 95.00 | 107.00 | 142.00 |
|  |  | **IR** | 1.00 | 0.00 | 0.00 | 0.00 | 2.00 | 8.25 | 2.00 | 5.00 | 3.25 | 4.00 | 2.00 | 8.00 | 16.00 | 7.00 | 8.00 | 7.00 | 33.00 |
|  | **6** | **N** | 16 | 16 | 16 | 16 | 16 | 16 | 16 | 15 | 15 | 15 | 15 | 15 | 15 | 11 | 11 | 11 | 11 |
|  |  | $\bar{\boldsymbol{x}}$ | 10.13 | 12.44 | 14.25 | 21.63 | 32.44 | 51.13 | 46.31 | 55.47 | 59.87 | 64.67 | 66.00 | 73.00 | 83.67 | 81.18 | 84.18 | 85.27 | 93.27 |
|  |  | **M** | 11.00 | 13.00 | 14.00 | 20.50 | 34.00 | 50.00 | 45.50 | 54.00 | 58.00 | 62.00 | 63.00 | 71.00 | 77.00 | 79.00 | 79.00 | 79.00 | 90.00 |
|  |  | **MR** | 442.19 | 452.97 | 463.56 | 462.44 | 438.78 | 181.56 | 422.91 | 118.90 | 128.73 | 137.97 | 90.57 | 74.27 | 75.07 | 165.55 | 104.82 | 47.14 | 28.32 |
|  |  | **Var** | 3.32 | 3.46 | 3.67 | 15.32 | 20.13 | 20.92 | 17.96 | 37.70 | 34.27 | 41.38 | 45.14 | 74.14 | 207.10 | 142.36 | 147.76 | 136.42 | 310.62 |
|  |  | **Min** | 8.00 | 10.00 | 13.00 | 18.00 | 23.00 | 47.00 | 41.00 | 49.00 | 55.00 | 60.00 | 61.00 | 64.00 | 70.00 | 68.00 | 74.00 | 75.00 | 75.00 |
|  |  | **Max** | 13.00 | 16.00 | 19.00 | 27.00 | 36.00 | 61.00 | 53.00 | 74.00 | 77.00 | 82.00 | 84.00 | 95.00 | 121.00 | 107.00 | 107.00 | 107.00 | 125.00 |
|  |  | **IR** | 3.00 | 2.00 | 1.00 | 7.00 | 7.00 | 6.00 | 7.50 | 7.00 | 5.00 | 7.00 | 6.00 | 6.00 | 14.00 | 19.00 | 14.00 | 14.00 | 28.00 |
|  | **7** | **N** | 8 | 8 | 8 | 8 | 8 | 8 | 8 | 8 | 8 | 8 | 7 | 7 | 7 | 7 | 7 | 7 | 7 |
|  |  | $\bar{\boldsymbol{x}}$ | 9.88 | 12.38 | 15.25 | 25.25 | 32.88 | 62.25 | 48.38 | 77.50 | 83.38 | 89.13 | 91.14 | 111.71 | 141.71 | 108.71 | 120.86 | 132.57 | 150.57 |
|  |  | **M** | 8.00 | 10.50 | 13.00 | 26.00 | 34.00 | 62.50 | 50.00 | 76.00 | 81.50 | 86.50 | 86.00 | 121.00 | 149.00 | 107.00 | 121.00 | 130.00 | 161.00 |
|  |  | **MR** | 416.81 | 420.94 | 449.94 | 514.94 | 474.69 | 432.31 | 460.81 | 380.19 | 375.88 | 369.00 | 343.14 | 291.14 | 271.29 | 299.00 | 273.71 | 237.43 | 165.93 |
|  |  | **Var** | 6.70 | 9.13 | 9.64 | 3.93 | 10.98 | 18.50 | 23.98 | 15.43 | 33.41 | 45.27 | 52.81 | 195.57 | 198.91 | 97.91 | 105.81 | 107.62 | 264.29 |
|  |  | **Min** | 8.00 | 10.00 | 13.00 | 23.00 | 29.00 | 56.00 | 42.00 | 74.00 | 77.00 | 82.00 | 84.00 | 97.00 | 121.00 | 97.00 | 107.00 | 121.00 | 130.00 |
|  |  | **Max** | 13.00 | 16.00 | 19.00 | 27.00 | 36.00 | 70.00 | 53.00 | 86.00 | 93.00 | 100.00 | 102.00 | 128.00 | 161.00 | 121.00 | 130.00 | 149.00 | 169.00 |
|  |  | **IR** | 5.00 | 6.00 | 6.00 | 4.00 | 7.00 | 5.25 | 10.00 | 4.50 | 9.00 | 11.00 | 11.00 | 24.00 | 21.00 | 24.00 | 23.00 | 21.00 | 31.00 |
|  | **8** | **N** |  |  |  |  |  |  |  |  |  |  |  |  |  |  |  |  |  |
|  |  | $\bar{\boldsymbol{x}}$ |  |  |  |  |  |  |  |  |  |  |  |  |  |  |  |  |  |
|  |  | **M** |  |  |  |  |  |  |  |  |  |  |  |  |  |  |  |  |  |
|  |  | **MR** |  |  |  |  |  |  |  |  |  |  |  |  |  |  |  |  |  |
|  |  | **Var** |  |  |  |  |  |  |  |  |  |  |  |  |  |  |  |  |  |
|  |  | **Min** |  |  |  |  |  |  |  |  |  |  |  |  |  |  |  |  |  |
|  |  | **Max** |  |  |  |  |  |  |  |  |  |  |  |  |  |  |  |  |  |
|  |  | **IR** |  |  |  |  |  |  |  |  |  |  |  |  |  |  |  |  |  |
|  | **9** | **N** | 24 | 24 | 24 | 24 | 24 | 24 | 24 | 23 | 23 | 23 | 23 | 23 | 23 | 9 | 9 | 9 | 9 |
|  |  | $\bar{\boldsymbol{x}}$ | 8.83 | 11.17 | 12.92 | 17.33 | 24.92 | 57.25 | 45.25 | 60.96 | 64.91 | 69.65 | 72.61 | 86.30 | 106.13 | 81.78 | 89.67 | 126.22 | 166.78 |
|  |  | **M** | 8.00 | 11.00 | 13.00 | 18.00 | 25.00 | 57.00 | 42.50 | 61.00 | 64.00 | 68.00 | 71.00 | 76.00 | 86.00 | 84.00 | 91.00 | 135.00 | 169.00 |
|  |  | **MR** | 393.44 | 409.21 | 398.46 | 382.25 | 211.58 | 335.35 | 381.96 | 234.80 | 230.02 | 230.67 | 184.52 | 151.70 | 152.22 | 188.28 | 154.78 | 215.06 | 218.83 |
|  |  | **Var** | 1.80 | 1.71 | 0.95 | 2.32 | 3.30 | 12.28 | 49.24 | 5.86 | 9.27 | 15.24 | 23.98 | 524.68 | 1921.21 | 16.69 | 22.75 | 652.94 | 1846.69 |
|  |  | **Min** | 7.00 | 9.00 | 11.00 | 14.00 | 23.00 | 53.00 | 40.00 | 58.00 | 61.00 | 65.00 | 68.00 | 68.00 | 68.00 | 74.00 | 77.00 | 91.00 | 91.00 |
|  |  | **Max** | 11.00 | 13.00 | 14.00 | 18.00 | 27.00 | 68.00 | 73.00 | 70.00 | 77.00 | 84.00 | 86.00 | 135.00 | 202.00 | 86.00 | 92.00 | 161.00 | 231.00 |
|  |  | **IR** | 2.50 | 1.50 | 0.75 | 0.00 | 4.00 | 2.75 | 9.00 | 2.00 | 2.00 | 2.00 | 6.00 | 26.00 | 73.00 | 6.00 | 0.50 | 50.50 | 67.50 |
|  | **12** | **N** | 16 | 16 | 16 | 16 | 16 | 8 | 8 | 8 | 8 | 8 | 8 | 8 | 8 | 8 | 8 | 8 | 8 |
|  |  | $\bar{\boldsymbol{x}}$ | 7.31 | 8.69 |  | 13.38 | 24.75 | 56.75 | 40.13 | 60.63 | 64.50 | 69.88 | 75.63 | 93.63 | 135.50 | 89.00 | 102.13 | 119.13 | 156.00 |
|  |  | **M** | 8.00 | 9.00 |  | 13.00 | 23.00 | 57.00 | 40.00 | 61.00 | 65.00 | 70.00 | 75.00 | 91.50 | 134.00 | 88.50 | 101.00 | 116.00 | 156.00 |
|  |  | **MR** | 261.47 | 242.13 | 229.00 | 144.13 | 195.44 | 330.50 | 264.19 | 235.06 | 230.00 | 234.81 | 233.06 | 217.44 | 244.81 | 231.50 | 218.31 | 184.75 | 181.06 |
|  |  | **Var** | 0.90 | 0.23 |  | 0.25 | 5.80 | 7.64 | 5.27 | 1.70 | 4.00 | 12.98 | 14.27 | 43.13 | 75.43 | 21.14 | 34.41 | 60.41 | 101.14 |
|  |  | **Min** | 6.00 | 8.00 |  | 13.00 | 23.00 | 53.00 | 37.00 | 58.00 | 61.00 | 65.00 | 71.00 | 88.00 | 125.00 | 84.00 | 97.00 | 111.00 | 142.00 |
|  |  | **Max** | 8.00 | 9.00 |  | 14.00 | 29.00 | 60.00 | 43.00 | 62.00 | 68.00 | 77.00 | 84.00 | 107.00 | 153.00 | 95.00 | 111.00 | 133.00 | 169.00 |
|  |  | **IR** | 2.00 | 1.00 |  | 1.00 | 3.50 | 5.75 | 4.75 | 1.75 | 1.75 | 4.25 | 2.00 | 9.25 | 11.00 | 9.50 | 11.75 | 12.75 | 20.25 |
|  | **10** | **N** | 24 | 24 | 24 | 24 | 24 | 24 | 24 | 16 | 16 | 16 | 16 | 16 | 16 | 16 | 16 | 16 | 16 |
|  |  | $\bar{\boldsymbol{x}}$ | 6.17 | 7.33 | 10.33 | 13.67 | 29.29 | 61.08 | 45.29 | 76.50 | 89.31 | 98.38 | 100.50 | 117.19 | 132.75 | 119.38 | 131.69 | 135.38 | 147.63 |
|  |  | **M** | 7.00 | 8.00 | 11.00 | 14.00 | 29.00 | 60.50 | 42.00 | 76.50 | 89.50 | 98.00 | 99.50 | 121.00 | 135.00 | 118.50 | 130.00 | 135.00 | 149.00 |
|  |  | **MR** | 145.58 | 100.50 | 171.83 | 191.67 | 380.85 | 413.65 | 378.67 | 373.47 | 408.13 | 412.03 | 383.00 | 316.19 | 235.81 | 327.22 | 309.19 | 250.41 | 155.00 |
|  |  | **Var** | 1.54 | 0.93 | 0.93 | 0.23 | 12.74 | 14.43 | 42.91 | 25.07 | 18.76 | 29.18 | 36.67 | 115.76 | 98.33 | 36.65 | 25.56 | 45.72 | 69.05 |
|  |  | **Min** | 4.00 | 6.00 | 9.00 | 13.00 | 23.00 | 56.00 | 39.00 | 70.00 | 84.00 | 91.00 | 92.00 | 97.00 | 116.00 | 111.00 | 125.00 | 125.00 | 128.00 |
|  |  | **Max** | 7.00 | 8.00 | 11.00 | 14.00 | 34.00 | 71.00 | 68.00 | 86.00 | 97.00 | 110.00 | 111.00 | 130.00 | 146.00 | 128.00 | 146.00 | 149.00 | 161.00 |
|  |  | **IR** | 2.00 | 2.00 | 2.00 | 1.00 | 7.00 | 5.75 | 8.25 | 7.25 | 7.50 | 5.50 | 8.75 | 17.00 | 20.50 | 10.00 | 6.50 | 8.00 | 12.25 |
|  | **11** | **N** | 24 | 24 | 24 | 24 | 24 | 24 | 24 | 8 | 8 | 8 | 8 | 8 | 8 | 1 | 1 | 1 | 1 |
|  |  | $\bar{\boldsymbol{x}}$ | 6.71 | 7.88 | 10.42 | 13.67 | 28.08 | 61.54 | 43.21 | 146.25 | 159.25 | 167.50 | 171.25 | 173.38 | 184.63 |  |  |  |  |
|  |  | **M** | 7.00 | 8.00 | 11.00 | 14.00 | 28.00 | 61.50 | 42.00 | 147.00 | 160.00 | 169.00 | 172.00 | 172.00 | 180.50 |  |  |  |  |
|  |  | **MR** | 209.06 | 157.02 | 178.98 | 191.67 | 343.67 | 417.40 | 360.46 | 482.50 | 481.50 | 477.50 | 468.50 | 464.31 | 422.13 | 378.00 | 378.00 | 374.50 | 296.00 |
|  |  | **Var** | 1.96 | 1.16 | 0.86 | 0.23 | 11.30 | 18.52 | 9.82 | 8.21 | 4.21 | 7.71 | 1.93 | 41.70 | 247.41 |  |  |  |  |
|  |  | **Min** | 4.00 | 6.00 | 9.00 | 13.00 | 23.00 | 54.00 | 40.00 | 142.00 | 156.00 | 163.00 | 169.00 | 169.00 | 169.00 |  |  |  |  |
|  |  | **Max** | 8.00 | 9.00 | 11.00 | 14.00 | 34.00 | 71.00 | 50.00 | 149.00 | 161.00 | 169.00 | 172.00 | 189.00 | 202.00 |  |  |  |  |
|  |  | **IR** | 2.00 | 2.00 | 2.00 | 1.00 | 3.50 | 6.00 | 3.00 | 5.75 | 3.75 | 4.50 | 2.25 | 2.25 | 32.25 |  |  |  |  |
|  | **13** | **N** | 24 | 24 | 24 | 24 | 24 | 24 | 24 | 16 | 16 | 16 | 15 | 15 | 15 | 15 | 15 | 15 | 15 |
|  |  | $\bar{\boldsymbol{x}}$ | 8.88 | 10.46 | 12.29 | 16.46 | 27.83 | 60.92 | 44.38 | 78.56 | 86.31 | 95.00 | 100.27 | 123.80 | 153.60 | 120.00 | 128.67 | 139.80 | 175.53 |
|  |  | **M** | 8.00 | 10.00 | 12.00 | 14.00 | 27.00 | 60.00 | 42.50 | 79.00 | 88.00 | 98.00 | 100.00 | 128.00 | 153.00 | 128.00 | 133.00 | 138.00 | 169.00 |
|  |  | **MR** | 367.69 | 358.67 | 326.29 | 326.75 | 332.50 | 409.81 | 382.71 | 388.50 | 392.59 | 399.13 | 380.13 | 344.40 | 322.57 | 329.90 | 305.20 | 271.13 | 246.03 |
|  |  | **Var** | 3.59 | 3.74 | 3.17 | 12.61 | 11.62 | 16.25 | 14.07 | 35.33 | 24.50 | 37.60 | 52.50 | 136.74 | 271.11 | 139.14 | 109.67 | 91.74 | 483.55 |
|  |  | **Min** | 6.00 | 8.00 | 11.00 | 14.00 | 23.00 | 56.00 | 40.00 | 68.00 | 77.00 | 85.00 | 86.00 | 100.00 | 121.00 | 92.00 | 102.00 | 121.00 | 138.00 |
|  |  | **Max** | 13.00 | 16.00 | 19.00 | 25.00 | 34.00 | 71.00 | 50.00 | 86.00 | 93.00 | 102.00 | 107.00 | 142.00 | 176.00 | 130.00 | 138.00 | 153.00 | 211.00 |
|  |  | **IR** | 3.00 | 3.00 | 2.00 | 4.00 | 4.00 | 5.00 | 7.00 | 6.00 | 5.00 | 9.00 | 12.00 | 14.00 | 30.00 | 17.00 | 12.00 | 19.00 | 33.00 |
| **Gh** | **1** | **N** | 24 | 24 | 24 | 24 | 24 | 24 | 24 | 24 | 24 | 24 | 24 | 24 | 24 |  |  |  |  |
|  |  | $\bar{\boldsymbol{x}}$ | 7.50 | 9.46 | 11.71 | 17.38 | 23.42 | 49.00 | 33.08 | 54.29 | 58.17 | 62.08 | 64.25 | 66.71 | 73.00 |  |  |  |  |
|  |  | **M** | 7.00 | 9.00 | 11.00 | 18.00 | 23.00 | 48.00 | 33.00 | 53.00 | 58.00 | 63.00 | 64.00 | 68.00 | 77.00 |  |  |  |  |
|  |  | **MR** | 250.40 | 279.29 | 291.48 | 368.69 | 119.08 | 120.52 | 78.35 | 108.06 | 110.92 | 109.00 | 71.42 | 32.88 | 39.19 |  |  |  |  |
|  |  | **Var** | 2.70 | 2.96 | 1.35 | 2.85 | 1.38 | 13.91 | 14.78 | 21.00 | 24.58 | 23.99 | 20.72 | 17.96 | 27.48 |  |  |  |  |
|  |  | **Min** | 6.00 | 8.00 | 11.00 | 13.00 | 23.00 | 46.00 | 29.00 | 48.00 | 51.00 | 54.00 | 58.00 | 58.00 | 64.00 |  |  |  |  |
|  |  | **Max** | 11.00 | 13.00 | 14.00 | 18.00 | 27.00 | 58.00 | 41.00 | 62.00 | 67.00 | 72.00 | 74.00 | 74.00 | 77.00 |  |  |  |  |
|  |  | **IR** | 2.00 | 3.00 | 2.00 | 0.00 | 0.00 | 5.00 | 6.75 | 7.00 | 7.50 | 8.00 | 7.00 | 5.75 | 9.00 |  |  |  |  |
|  | **2** | **N** | 24 | 24 | 24 | 24 | 24 | 24 | 24 | 24 | 24 | 23 | 23 | 23 | 23 | 23 | 23 | 23 | 23 |
|  |  | $\bar{\boldsymbol{x}}$ | 7.42 | 10.00 | 12.17 | 16.21 | 25.63 | 52.88 | 39.83 | 64.79 | 68.63 | 72.87 | 83.17 | 128.39 | 170.65 | 80.43 | 105.43 | 140.91 | 195.04 |
|  |  | **M** | 8.00 | 11.00 | 13.00 | 18.00 | 24.00 | 53.00 | 39.00 | 62.50 | 67.00 | 71.00 | 79.00 | 125.00 | 172.00 | 77.00 | 102.00 | 135.00 | 189.00 |
|  |  | **MR** | 274.13 | 334.29 | 332.83 | 277.85 | 218.69 | 230.98 | 241.21 | 256.40 | 246.52 | 240.24 | 284.76 | 349.13 | 379.80 | 167.13 | 217.52 | 259.50 | 301.67 |
|  |  | **Var** | 0.69 | 1.57 | 1.01 | 9.65 | 14.33 | 17.33 | 36.15 | 62.69 | 73.46 | 83.03 | 114.70 | 679.43 | 470.69 | 77.98 | 354.71 | 710.27 | 504.95 |
|  |  | **Min** | 6.00 | 8.00 | 11.00 | 13.00 | 23.00 | 49.00 | 29.00 | 55.00 | 56.00 | 60.00 | 69.00 | 86.00 | 130.00 | 68.00 | 77.00 | 100.00 | 161.00 |
|  |  | **Max** | 8.00 | 11.00 | 13.00 | 25.00 | 34.00 | 63.00 | 51.00 | 76.00 | 82.00 | 85.00 | 111.00 | 172.00 | 202.00 | 92.00 | 146.00 | 189.00 | 222.00 |
|  |  | **IR** | 1.00 | 2.00 | 2.00 | 5.00 | 5.00 | 6.00 | 7.25 | 16.00 | 17.00 | 20.00 | 14.00 | 49.00 | 33.00 | 18.00 | 24.00 | 53.00 | 44.00 |
|  | **3** | **N** | 24 | 24 | 24 | 24 | 24 | 24 | 24 | 24 | 24 | 24 | 24 | 24 | 24 | 24 | 24 | 24 | 24 |
|  |  | $\bar{\boldsymbol{x}}$ | 6.88 | 8.17 | 10.58 | 13.71 | 23.25 | 47.63 | 38.38 | 53.50 | 57.25 | 60.92 | 68.33 | 86.54 | 115.00 | 69.21 | 79.33 | 96.79 | 129.79 |
|  |  | **M** | 7.00 | 8.00 | 11.00 | 14.00 | 23.00 | 48.00 | 37.50 | 54.00 | 58.00 | 61.00 | 66.50 | 84.00 | 107.00 | 69.50 | 77.00 | 92.00 | 121.00 |
|  |  | **MR** | 196.10 | 166.23 | 193.81 | 198.46 | 111.25 | 83.21 | 194.54 | 90.46 | 90.67 | 85.44 | 126.27 | 171.79 | 191.83 | 69.10 | 78.63 | 95.54 | 111.27 |
|  |  | **Var** | 0.90 | 1.28 | 1.38 | 0.22 | 0.46 | 1.55 | 8.85 | 6.52 | 6.54 | 7.91 | 30.15 | 193.82 | 852.87 | 11.30 | 45.36 | 242.17 | 779.82 |
|  |  | **Min** | 6.00 | 7.00 | 9.00 | 13.00 | 23.00 | 46.00 | 36.00 | 48.00 | 51.00 | 56.00 | 63.00 | 70.00 | 77.00 | 64.00 | 69.00 | 77.00 | 90.00 |
|  |  | **Max** | 9.00 | 11.00 | 13.00 | 14.00 | 25.00 | 49.00 | 48.00 | 58.00 | 61.00 | 65.00 | 79.00 | 125.00 | 172.00 | 74.00 | 92.00 | 133.00 | 189.00 |
|  |  | **IR** | 1.00 | 2.00 | 2.00 | 1.00 | 0.00 | 3.00 | 5.00 | 3.50 | 2.75 | 3.75 | 9.50 | 15.00 | 54.00 | 6.00 | 10.00 | 21.25 | 52.00 |
|  | **4** | **N** | 24 | 24 | 24 | 24 | 24 | 24 | 24 | 24 | 23 | 23 | 22 | 22 | 22 | 22 | 22 | 22 | 22 |
|  |  | $\bar{\boldsymbol{x}}$ | 7.8333 | 9.5 | 11.3333 | 15.9167 | 27.5417 | 56.4167 | 38 | 65.7917 | 69.2174 | 74.0435 | 82.4545 | 109.5455 | 150.4091 | 79.5 | 97.1818 | 122 | 170.1364 |
|  |  | **M** | 8.5 | 11 | 13 | 18 | 27 | 55 | 37.5 | 64 | 68 | 75 | 86 | 101 | 153 | 82 | 96 | 116 | 169 |
|  |  | **MR** | 310.50 | 273.17 | 261.38 | 273.46 | 329.31 | 316.67 | 185.88 | 285.08 | 270.20 | 267.96 | 278.93 | 285.16 | 307.55 | 161.20 | 180.89 | 198.70 | 229.02 |
|  |  | **Var** | 1.71 | 3.391 | 4.058 | 6.341 | 9.911 | 16.949 | 18.348 | 32.868 | 40.905 | 48.407 | 81.974 | 395.212 | 307.968 | 68.643 | 176.918 | 356.952 | 469.742 |
|  |  | **Min** | 6 | 7 | 9 | 13 | 23 | 51 | 30 | 58 | 60 | 62 | 68 | 86 | 111 | 65 | 74 | 100 | 135 |
|  |  | **Max** | 9 | 11 | 13 | 18 | 34 | 65 | 48 | 74 | 81 | 88 | 102 | 156 | 172 | 97 | 121 | 165 | 222 |
|  |  | **IR** | 2.75 | 3.75 | 4 | 5 | 4 | 5 | 4.5 | 11.5 | 8 | 10 | 15.25 | 33 | 34 | 12.75 | 25 | 26.25 | 27.25 |
|  | **5** | **N** | 24 | 24 | 24 | 24 | 24 | 24 | 24 | 24 | 24 | 24 | 24 | 24 | 24 | 24 | 24 | 24 | 24 |
|  |  | $\bar{\boldsymbol{x}}$ | 7.13 | 8.50 | 9.63 | 13.63 | 28.29 | 48.13 | 40.54 | 54.04 | 57.71 | 62.00 | 70.96 | 94.00 | 121.75 | 68.63 | 83.13 | 103.67 | 143.29 |
|  |  | **M** | 7.00 | 8.00 | 9.00 | 13.00 | 27.00 | 48.00 | 39.00 | 53.00 | 56.00 | 62.00 | 70.00 | 85.00 | 125.00 | 69.00 | 76.00 | 93.50 | 146.00 |
|  |  | **MR** | 197.94 | 175.06 | 111.75 | 123.81 | 348.81 | 94.96 | 252.85 | 96.04 | 97.75 | 102.27 | 162.23 | 202.04 | 211.81 | 64.00 | 97.71 | 122.63 | 154.88 |
|  |  | **Var** | 2.46 | 3.13 | 2.85 | 2.85 | 12.91 | 9.25 | 22.09 | 31.35 | 36.04 | 32.52 | 44.39 | 462.17 | 1229.94 | 30.51 | 156.90 | 518.67 | 1660.13 |
|  |  | **Min** | 6.00 | 7.00 | 9.00 | 13.00 | 23.00 | 46.00 | 34.00 | 48.00 | 50.00 | 54.00 | 61.00 | 70.00 | 74.00 | 61.00 | 69.00 | 74.00 | 88.00 |
|  |  | **Max** | 11.00 | 13.00 | 14.00 | 18.00 | 36.00 | 58.00 | 49.00 | 75.00 | 79.00 | 82.00 | 86.00 | 133.00 | 172.00 | 88.00 | 111.00 | 146.00 | 216.00 |
|  |  | **IR** | 1.00 | 0.00 | 0.00 | 0.00 | 4.00 | 3.00 | 5.00 | 4.00 | 5.75 | 4.00 | 9.00 | 44.50 | 63.25 | 6.00 | 17.00 | 43.50 | 68.00 |
|  | **6** | **N** | 24 | 24 | 24 | 24 | 24 | 24 | 24 | 24 | 24 | 24 | 24 | 24 | 24 | 24 | 24 | 24 | 24 |
|  |  | $\bar{\boldsymbol{x}}$ | 8.25 | 10.00 | 11.50 | 14.96 | 26.33 | 49.04 | 39.33 | 52.46 | 55.38 | 58.29 | 62.63 | 76.00 | 99.29 | 63.83 | 71.42 | 85.54 | 116.17 |
|  |  | **M** | 7.50 | 9.00 | 11.00 | 14.00 | 25.00 | 49.00 | 36.00 | 51.00 | 55.00 | 57.00 | 62.00 | 74.50 | 93.00 | 63.00 | 71.50 | 82.00 | 113.50 |
|  |  | **MR** | 295.71 | 310.79 | 273.63 | 239.38 | 268.50 | 128.21 | 215.10 | 66.58 | 58.69 | 54.40 | 56.85 | 104.13 | 134.10 | 29.50 | 28.19 | 50.75 | 79.42 |
|  |  | **Var** | 4.37 | 5.04 | 3.91 | 5.00 | 10.58 | 6.39 | 46.41 | 8.52 | 13.90 | 19.78 | 21.29 | 85.74 | 624.82 | 19.80 | 30.08 | 135.48 | 733.62 |
|  |  | **Min** | 6.00 | 7.00 | 9.00 | 13.00 | 23.00 | 46.00 | 30.00 | 49.00 | 50.00 | 54.00 | 55.00 | 61.00 | 64.00 | 58.00 | 62.00 | 64.00 | 64.00 |
|  |  | **Max** | 11.00 | 13.00 | 14.00 | 18.00 | 34.00 | 55.00 | 49.00 | 60.00 | 63.00 | 67.00 | 71.00 | 97.00 | 142.00 | 74.00 | 86.00 | 111.00 | 156.00 |
|  |  | **IR** | 4.75 | 4.75 | 4.50 | 5.00 | 6.00 | 5.00 | 14.00 | 2.75 | 4.50 | 6.75 | 6.75 | 13.50 | 39.50 | 4.75 | 5.75 | 15.00 | 48.50 |
|  | **7** | **N** | 16 | 16 | 16 | 16 | 16 | 16 | 16 | 16 | 16 | 16 | 16 | 16 | 16 | 16 | 16 | 16 | 16 |
|  |  | $\bar{\boldsymbol{x}}$ | 7.31 | 10.88 | 12.88 | 18.00 | 25.00 | 49.75 | 37.06 | 54.44 | 57.94 | 62.13 | 71.13 | 99.81 | 142.00 | 71.44 | 85.81 | 114.31 | 163.31 |
|  |  | **M** | 7.00 | 11.00 | 13.00 | 18.00 | 25.00 | 49.00 | 36.00 | 54.00 | 56.00 | 61.00 | 69.50 | 95.00 | 147.50 | 68.00 | 82.00 | 109.00 | 165.00 |
|  |  | **MR** | 199.97 | 349.88 | 318.44 | 365.53 | 220.06 | 149.03 | 162.34 | 104.31 | 99.69 | 102.53 | 164.38 | 236.03 | 288.59 | 82.09 | 118.13 | 168.34 | 208.09 |
|  |  | **Var** | 9.16 | 7.85 | 11.45 | 10.00 | 3.20 | 3.40 | 11.80 | 8.00 | 9.13 | 8.25 | 32.78 | 410.03 | 1080.80 | 80.53 | 143.10 | 392.63 | 1445.70 |
|  |  | **Min** | 4.00 | 8.00 | 9.00 | 13.00 | 23.00 | 48.00 | 30.00 | 51.00 | 55.00 | 58.00 | 64.00 | 72.00 | 91.00 | 65.00 | 75.00 | 82.00 | 107.00 |
|  |  | **Max** | 13.00 | 16.00 | 19.00 | 23.00 | 29.00 | 53.00 | 41.00 | 58.00 | 63.00 | 67.00 | 82.00 | 142.00 | 189.00 | 102.00 | 111.00 | 153.00 | 222.00 |
|  |  | **IR** | 1.00 | 2.00 | 2.00 | 0.00 | 3.50 | 2.75 | 7.00 | 6.50 | 5.75 | 5.00 | 11.25 | 32.50 | 69.00 | 5.50 | 18.75 | 29.75 | 54.00 |
|  | **8** | **N** | 16 | 16 | 16 | 16 | 16 | 16 | 16 | 16 | 16 | 16 | 16 | 16 | 16 | 2 | 2 | 2 | 2 |
|  |  | $\bar{\boldsymbol{x}}$ | 7.06 | 10.00 | 11.25 | 15.50 | 24.38 | 50.69 | 39.63 | 58.13 | 61.69 | 65.31 | 67.13 | 67.88 | 71.00 |  |  |  |  |
|  |  | **M** | 7.00 | 9.50 | 11.00 | 15.50 | 23.00 | 51.00 | 39.00 | 56.50 | 60.50 | 64.00 | 66.00 | 69.00 | 73.00 |  |  |  |  |
|  |  | **MR** | 206.59 | 297.38 | 253.38 | 246.25 | 175.69 | 173.75 | 218.66 | 175.31 | 165.50 | 155.69 | 115.44 | 47.88 | 28.38 | 117.50 | 33.00 | 4.50 | 9.50 |
|  |  | **Var** | 6.86 | 4.80 | 5.53 | 6.67 | 4.12 | 5.43 | 28.25 | 33.32 | 37.56 | 40.90 | 41.05 | 35.45 | 32.67 |  |  |  |  |
|  |  | **Min** | 4.00 | 8.00 | 9.00 | 13.00 | 23.00 | 48.00 | 33.00 | 51.00 | 55.00 | 58.00 | 60.00 | 60.00 | 60.00 |  |  |  |  |
|  |  | **Max** | 11.00 | 13.00 | 14.00 | 18.00 | 29.00 | 55.00 | 48.00 | 67.00 | 71.00 | 75.00 | 77.00 | 77.00 | 77.00 |  |  |  |  |
|  |  | **IR** | 5.50 | 4.50 | 4.75 | 5.00 | 3.50 | 3.50 | 9.75 | 9.75 | 11.00 | 11.50 | 12.25 | 11.50 | 6.00 |  |  |  |  |
|  | **9** | **N** | 24 | 24 | 24 | 24 | 24 | 24 | 24 | 24 | 24 | 24 | 24 | 24 | 24 | 16 | 16 | 16 | 16 |
|  |  | $\bar{\boldsymbol{x}}$ | 8.88 | 11.17 | 12.92 | 17.33 | 23.75 | 49.08 | 36.58 | 54.58 | 58.58 | 62.63 | 71.46 | 105.38 | 137.21 | 79.38 | 99.63 | 117.50 | 182.44 |
|  |  | **M** | 8.00 | 11.00 | 13.00 | 18.00 | 23.00 | 49.00 | 36.00 | 53.00 | 56.00 | 61.00 | 71.00 | 104.50 | 135.00 | 75.00 | 90.00 | 125.00 | 169.00 |
|  |  | **MR** | 399.50 | 409.21 | 398.46 | 382.25 | 140.46 | 127.48 | 146.85 | 105.94 | 108.54 | 109.46 | 167.81 | 258.90 | 261.58 | 155.91 | 178.00 | 180.78 | 215.41 |
|  |  | **Var** | 1.68 | 1.71 | 0.95 | 2.32 | 2.37 | 8.95 | 21.47 | 22.95 | 22.95 | 24.85 | 65.91 | 310.25 | 750.96 | 113.85 | 483.32 | 409.87 | 8108.80 |
|  |  | **Min** | 8.00 | 9.00 | 11.00 | 14.00 | 23.00 | 46.00 | 26.00 | 48.00 | 55.00 | 58.00 | 60.00 | 74.00 | 91.00 | 69.00 | 74.00 | 86.00 | 107.00 |
|  |  | **Max** | 11.00 | 13.00 | 14.00 | 18.00 | 29.00 | 58.00 | 48.00 | 67.00 | 71.00 | 75.00 | 91.00 | 128.00 | 189.00 | 102.00 | 130.00 | 142.00 | 495.00 |
|  |  | **IR** | 2.50 | 1.50 | 0.75 | 0.00 | 1.50 | 5.00 | 2.00 | 4.00 | 5.00 | 3.75 | 12.75 | 30.00 | 32.00 | 6.50 | 47.50 | 38.00 | 62.00 |
|  | **12** | **N** | 24 | 24 | 24 | 24 | 24 | 24 | 24 | 24 | 24 | 24 | 24 | 24 | 24 | 24 | 24 | 24 | 24 |
|  |  | $\bar{\boldsymbol{x}}$ | 6.88 | 8.46 |  | 13.42 | 23.25 | 49.42 | 40.50 | 64.71 | 68.63 | 72.13 | 78.63 | 101.46 | 140.04 | 78.88 | 92.63 | 112.04 | 155.67 |
|  |  | **M** | 6.00 | 8.00 |  | 13.00 | 23.00 | 48.00 | 41.00 | 60.00 | 64.00 | 67.00 | 74.50 | 98.50 | 146.00 | 74.50 | 89.50 | 109.00 | 158.50 |
|  |  | **MR** | 203.31 | 209.58 | 229.00 | 150.92 | 111.25 | 127.73 | 263.96 | 235.94 | 230.02 | 216.21 | 231.63 | 242.77 | 272.17 | 144.90 | 150.75 | 158.29 | 184.96 |
|  |  | **Var** | 0.98 | 0.26 |  | 0.25 | 0.46 | 23.38 | 16.70 | 162.82 | 175.29 | 189.59 | 208.16 | 442.09 | 637.87 | 187.33 | 297.81 | 480.39 | 741.62 |
|  |  | **Min** | 6.00 | 8.00 |  | 13.00 | 23.00 | 46.00 | 34.00 | 51.00 | 55.00 | 58.00 | 61.00 | 70.00 | 97.00 | 63.00 | 71.00 | 75.00 | 116.00 |
|  |  | **Max** | 8.00 | 9.00 |  | 14.00 | 25.00 | 63.00 | 49.00 | 100.00 | 106.00 | 111.00 | 125.00 | 146.00 | 189.00 | 121.00 | 135.00 | 156.00 | 211.00 |
|  |  | **IR** | 2.00 | 1.00 |  | 1.00 | 0.00 | 5.00 | 4.25 | 14.50 | 14.75 | 16.00 | 10.75 | 36.50 | 45.00 | 12.00 | 18.75 | 41.75 | 49.25 |
|  | **10** | **N** | 24 | 24 | 24 | 24 | 24 | 24 | 24 | 24 | 24 | 23 | 23 | 23 | 23 | 23 | 23 | 23 | 23 |
|  |  | $\bar{\boldsymbol{x}}$ | 6.17 | 7.33 | 10.33 | 13.67 | 26.92 | 63.08 | 35.25 | 77.08 | 81.67 | 85.83 | 100.13 | 132.17 | 172.22 | 95.74 | 117.78 | 142.30 | 186.65 |
|  |  | **M** | 7.00 | 8.00 | 11.00 | 14.00 | 27.00 | 63.00 | 34.00 | 77.00 | 82.00 | 86.00 | 100.00 | 142.00 | 172.00 | 97.00 | 121.00 | 153.00 | 189.00 |
|  |  | **MR** | 145.58 | 100.50 | 171.83 | 191.67 | 282.88 | 401.60 | 109.58 | 363.58 | 355.38 | 343.80 | 358.48 | 371.46 | 392.74 | 246.17 | 260.67 | 278.33 | 283.28 |
|  |  | **Var** | 1.54 | 0.93 | 0.93 | 0.23 | 13.91 | 71.73 | 19.50 | 220.51 | 218.32 | 225.24 | 368.76 | 432.42 | 249.45 | 277.20 | 420.36 | 410.77 | 292.24 |
|  |  | **Min** | 4.00 | 6.00 | 9.00 | 13.00 | 23.00 | 51.00 | 29.00 | 61.00 | 65.00 | 70.00 | 72.00 | 88.00 | 146.00 | 75.00 | 79.00 | 95.00 | 156.00 |
|  |  | **Max** | 7.00 | 8.00 | 11.00 | 14.00 | 34.00 | 75.00 | 49.00 | 128.00 | 132.00 | 137.00 | 146.00 | 156.00 | 189.00 | 146.00 | 153.00 | 172.00 | 211.00 |
|  |  | **IR** | 2.00 | 2.00 | 2.00 | 1.00 | 4.00 | 15.00 | 2.00 | 17.00 | 18.50 | 19.00 | 27.00 | 35.00 | 33.00 | 20.00 | 38.00 | 34.00 | 33.00 |
|  | **11** | **N** | 24 | 24 | 24 | 24 | 24 | 24 | 24 | 24 | 24 | 24 | 24 | 24 | 24 | 18 | 18 | 18 | 18 |
|  |  | $\bar{\boldsymbol{x}}$ | 6.71 | 7.88 | 10.42 | 13.67 | 26.71 | 63.33 | 36.83 | 123.50 | 127.29 | 134.08 | 137.67 | 149.17 | 160.13 | 141.78 | 146.78 | 161.78 | 179.56 |
|  |  | **M** | 7.00 | 8.00 | 11.00 | 14.00 | 25.00 | 63.00 | 35.00 | 125.00 | 130.00 | 135.00 | 138.00 | 153.00 | 169.00 | 142.00 | 149.00 | 169.00 | 189.00 |
|  |  | **MR** | 209.06 | 157.02 | 178.98 | 191.67 | 287.90 | 428.19 | 141.81 | 465.65 | 464.65 | 460.79 | 451.08 | 427.33 | 347.50 | 367.67 | 359.06 | 337.83 | 266.75 |
|  |  | **Var** | 1.96 | 1.16 | 0.86 | 0.23 | 10.48 | 41.19 | 19.10 | 19.04 | 24.04 | 19.38 | 22.84 | 85.88 | 197.42 | 35.01 | 56.54 | 159.71 | 337.09 |
|  |  | **Min** | 4.00 | 6.00 | 9.00 | 13.00 | 23.00 | 55.00 | 33.00 | 116.00 | 119.00 | 126.00 | 128.00 | 128.00 | 138.00 | 130.00 | 130.00 | 130.00 | 130.00 |
|  |  | **Max** | 8.00 | 9.00 | 11.00 | 14.00 | 34.00 | 75.00 | 48.00 | 128.00 | 132.00 | 138.00 | 142.00 | 161.00 | 172.00 | 149.00 | 153.00 | 172.00 | 202.00 |
|  |  | **IR** | 2.00 | 2.00 | 2.00 | 1.00 | 2.00 | 10.00 | 5.00 | 5.00 | 6.00 | 2.00 | 4.00 | 17.00 | 29.00 | 8.75 | 7.00 | 9.25 | 14.00 |
|  | **13** | **N** | 24 | 24 | 24 | 24 | 24 | 24 | 24 | 24 | 24 | 24 | 24 | 24 | 24 | 24 | 24 | 24 | 24 |
|  |  | $\bar{\boldsymbol{x}}$ | 7.71 | 9.42 | 11.50 | 15.00 | 26.75 | 51.88 | 37.67 | 92.25 | 96.79 | 102.33 | 113.46 | 145.58 | 184.67 | 108.92 | 130.33 | 158.13 | 202.50 |
|  |  | **M** | 8.00 | 9.00 | 11.00 | 14.00 | 25.00 | 51.00 | 36.00 | 88.00 | 93.00 | 97.50 | 111.00 | 138.50 | 169.00 | 104.50 | 135.00 | 151.00 | 189.00 |
|  |  | **MR** | 313.56 | 305.29 | 273.50 | 286.88 | 276.63 | 192.25 | 174.21 | 428.92 | 421.31 | 411.63 | 409.79 | 407.17 | 402.83 | 299.48 | 313.65 | 320.13 | 318.42 |
|  |  | **Var** | 0.39 | 0.95 | 0.78 | 3.13 | 15.07 | 40.81 | 27.19 | 135.94 | 136.26 | 150.67 | 172.17 | 374.43 | 512.23 | 147.65 | 173.10 | 413.59 | 548.78 |
|  |  | **Min** | 6.00 | 8.00 | 11.00 | 14.00 | 23.00 | 44.00 | 29.00 | 76.00 | 81.00 | 85.00 | 92.00 | 116.00 | 169.00 | 88.00 | 107.00 | 135.00 | 176.00 |
|  |  | **Max** | 8.00 | 11.00 | 13.00 | 18.00 | 36.00 | 68.00 | 48.00 | 110.00 | 114.00 | 121.00 | 133.00 | 176.00 | 216.00 | 128.00 | 146.00 | 189.00 | 237.00 |
|  |  | **IR** | 0.00 | 1.50 | 1.50 | 3.00 | 2.00 | 5.00 | 2.25 | 23.00 | 24.00 | 26.00 | 24.50 | 38.25 | 47.00 | 24.00 | 25.75 | 34.00 | 42.00 |
